# Unveiling the trophic dynamics and ecological roles of demersal fish in Hong Kong: A metabarcoding and isotope analysis approach

**DOI:** 10.1371/journal.pone.0335343

**Published:** 2025-11-13

**Authors:** Hei-Ching Wong, Chun-Ming How, Kelly Su, Leyi Xi, Chien-Hsiang Lin, Ming-Tsung Chung, Jian-Wen Qiu, Chris Kong-Chu Wong, Jill Man-Ying Chiu

**Affiliations:** 1 Department of Biology, Hong Kong Baptist University, Hong Kong SAR, China; 2 Department of Humanities and Sciences, Stanford University, Stanford, California, United States of America; 3 Biodiversity Research Center, Academia Sinica, Taipei, Taiwan; 4 Institute of Oceanography, National Taiwan University, Taipei, Taiwan; Central Marine Fisheries Research Institute, INDIA

## Abstract

This study provides a comprehensive examination of the trophic ecology and feeding dynamics of 16 demersal fish species inhabiting the southern and southwestern waters of Hong Kong, utilizing advanced 12S and COI gut content metabarcoding alongside stable isotope analysis. Dietary dissimilarities, primarily driven by Decapoda and fish, are significant among species. Network plots further highlight unique predator-prey interactions. Eight species, including horn dragonet *Callionymus curvicornis* and Japanese butterflyray *Gymnura japonica*, are identified as piscivores, primarily preying on demersal fish, while six species such as rough flathead *Grammoplites scaber* and Japanese flathead *Inegocia japonica* are classified as crustacean feeders, focusing on Decapoda. Notably, spotted sicklefish *Drepane punctata* and goatee croaker *Dendrophysa russelii* exhibit unique feeding behaviors, relying on brittle star and Bivalvia, respectively, and demonstrate non-selective feeding patterns that do not prioritize dominant environmental species. This diverse range of prey consumption highlights the critical roles these fish play in regulating demersal fish and benthic invertebrate communities. The study also reveals clear trophic niche partitioning with low isotope niche overlap, predominantly below 55.30%, except for a notable overlap of 72.91% between bartail flathead *Platycephalus indicus* and goatee croaker *D. russelii*. Our results established essential baseline data on trophic niche diversification and resource partitioning through varied dietary preferences, facilitating coexistence and resilience within the ecosystem. This research serves as a foundational assessment of the trophic dynamics and ecological stability in Hong Kong’s marine ecosystems, offering valuable insights into anthropogenic pressures and guiding the development of specific conservation strategies aimed at preserving fish biodiversity and sustaining global fisheries.

## Introduction

In trophic ecology, dietary overlap (or diversification) is a key indicator of competition among organisms, reflecting the extent to which species utilize similar resources, with prey community data offering insights into resource availability [1,2]. Marine fish often mitigate dietary competition by adopting diverse foraging strategies and prey preferences, supported by unique morphological adaptations, such as flattened bodies for sit-and-wait predation or barbel-equipped inferior mouths for foraging on infaunal prey, enhancing their survival and success. Meanwhile, trophic niche partitioning is a crucial ecological process that reduces competition by allowing species to share niches in their environments or habitats, preventing extinction due to competitive exclusion [3]. By making trade-offs in their realized niches, such as selecting different habitats (spatial partitioning), varying activity periods (temporal partitioning), or choosing distinct prey (resource partitioning), species can coexist within the same community, thereby promoting species diversity and sustaining complex food chains and webs [4–8]. However, global overfishing poses a significant threat by altering the trophic structure of food webs and narrowing the trophic niches of fish species, as it reduces the diversity of available nutrient sources [9]. This underscores the importance of understanding dietary diversification and niche partitioning to ensure ecosystem stability and resilience.

In the marine ecosystems, demersal fish play an essential role in facilitating energy transfer between the bottom seafloor and the water column, serving as prey for larger predators, and supporting global fisheries. They can be classified into two main groups, including epibenthic (inhabit the seabed with direct physical contact) and hyperbenthic (inhabit the water column just above the bottom) [10,11]. They face high fishing pressure and the loss of benthic habitats caused by the continuously increasing number of coastal developments.

Hong Kong’s water can be divided into a western estuarine zone influenced by the Pearl River Estuary and an eastern oceanic zone shaped by ocean currents, hosting a rich diversity of 1,260 recorded fish species [12,13]. Bottom trawling was historically the dominant fishing practice in Hong Kong, accounting for 50% of the total fisheries landings from local waters, with an annual catch of around 12,000 tonnes in 2006, and accounted for 80% of the total fishing efforts in 2010 [14–16]. However, its non-selective nature and destructive impact on the seabed made it the major driver of the severe decline of fishery landings during the late 1970s, both in absolute terms and catch per unit effort [17,18]. Depletion of large predatory fish, such as groupers (Serranidae) and seabreams (Sparidae), was observed, causing the phenomenon of ‘fishing down marine food webs’ [19,20]. Although the Hong Kong SAR Government implemented a trawl ban in 2012 to restore the damaged seabed and depleted fish stocks, intensive fishing efforts by commercial and recreational fishing activities persist, as well as large-scale coastal infrastructures, including Hong Kong-Zhuhai-Macao Bridge and the Third-Runway System at Hong Kong International Airport, have continued to destroy the benthic habitats, leading to habitat loss and fragmentation [21]. Leung [22] has also documented that reclamation and dredging disrupt the benthic fish community in the southeastern waters by impacting species diversity and abundance. Consequently, some commercially important fish species, such as the Chinese bahaba (*Bahaba taipingensis*) and large yellow croaker (*Larimichthys crocea*), are now critically endangered [23,24].

To protect and conserve the local marine environment and resources, marine protected areas (MPAs), including eight marine parks and one marine reserve, have been established in Hong Kong since 1996, covering around 5.2% of the sea area, with commercial fishing prohibited in four of these designated marine parks. That same year, restoration actions, including the deployment of artificial reef projects and fish restocking exercises, were launched in marine parks, important fish spawning and nursery grounds, and fish culture zones. The reefs enhance benthic habitat quality by increasing the complexity and promoting biodiversity, while restocking restores native and commercially important species and conserves fisheries resources.

Only one study has examined the feeding ecology of marine fish in Hong Kong, focusing specifically on the larvae and juveniles of black seabream (*Acanthopagrus schlegeli*) and Japanese seaperch (*Lateolabrax japonicus*) [25]. Due to the scarcity of dietary information, understanding the trophic interactions within the food web and the impacts of anthropogenic activities on fish communities has been challenging. This study aims to investigate the trophic dynamics of demersal fish species in the southern and southwestern waters of Hong Kong, taking advantage of the region’s unique ecological environment and significant anthropogenic pressures. Addressing the knowledge gap on fish feeding habits in Hong Kong waters, we hypothesize that morphologically similar species in this highly urbanized environment would exhibit high competition and hence, dietary overlap due to limited resources [26].

Exploring dietary overlaps (or diversification) and niche partitioning is essential, as these processes can significantly influence the stability and resilience of ecosystems. To investigate this, we conducted trawling in Hong Kong’s southern and southwestern waters, aiming to sample demersal fish species and their potential prey, including smaller fish and marine invertebrates.

By utilizing both gut content metabarcoding and stable isotope analysis, we identified the food composition and trophic positions of 16 demersal fish species.

Stable isotopes provide insights into energy flow within food webs by analyzing isotopic compositions in consumers and their prey, offering a window into ecological community dynamics [27]. Carbon stable isotopes (^13^C/^12^C) reflect the photosynthetic pathways of primary producers, distinguishing between benthic and pelagic energy sources, while nitrogen stable isotopes (^15^N/^14^N) indicate trophic levels, enriched progressively in organisms at higher trophic positions [28,29]. These isotopic signals are integrated into consumer tissues, making them effective for tracing energy flow and identifying food sources in aquatic ecosystems [30–32]. Stable carbon isotopes distinguish between pelagic production (depleted values) and benthic production (enriched values), identifying major energy sources and habitats. Meanwhile, nitrogen isotopes offer a proxy for trophic level, enabling the detection of resource choices and trophic interactions within food webs.

Stable isotope analysis provides several benefits compared to traditional gut content analysis. It offers a long-term dietary perspective, as isotope signatures reflect assimilated energy sources over time rather than the short-term prey ingestion seen in gut content analysis. Additionally, it avoids observer bias associated with prey identification and serves as a quantitative tool for assessing niche overlap and trophic niche dimensions [33,34]. DNA metabarcoding complements stable isotope analysis by capturing short-term dietary habits with high taxonomic resolution, even identifying degraded prey DNA, which traditional methods may overlook [35,36].

Integrating stable isotope and DNA metabarcoding techniques offers a comprehensive framework for studying trophic interactions by linking short-term prey consumption with long-term dietary assimilation. Stable isotope analysis provides insights into trophic positions and energy pathways over time, while DNA metabarcoding resolves taxonomic details of recently consumed prey with high precision. Together, these methods enable a deeper understanding of resource partitioning, dietary specialization, and habitat use in fish communities. This integrated approach is particularly valuable for assessing ecological dynamics in urbanized and biodiversity-rich marine ecosystems, such as those in Hong Kong, where anthropogenic pressures and habitat changes can drastically alter food web structures.

## Materials and methods

### Survey and specimen collection for fish and potential prey items

**Fig 1:**
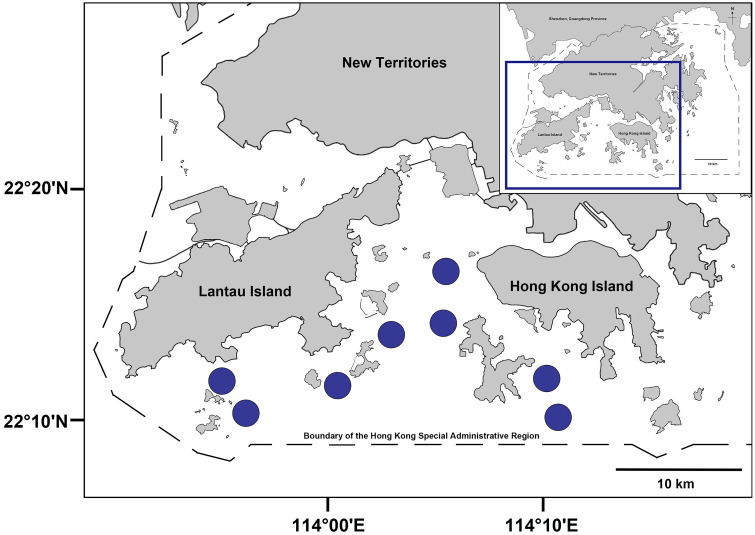
Map of Hong Kong showing the sampling location of fish specimens and potential prey items.

Fish species and their potential prey items, including smaller fish and marine invertebrates, were sampled in Hong Kong’s southern and southwestern waters during the onset of the wet season (April to May 2022, total rainfall: 440 mm, as reported by the Hong Kong Observatory). Sampling was conducted via daytime trawling under a scientific permit (R1710058) (Fig 1). Daytime sampling during the wet season was strategically chosen, as most local fish species are diurnal, and rising temperatures combined with an increased abundance of mesozooplankton prey, such as decapods, stimulate heightened feeding activity. This period of intense feeding behavior enhances the detectability of fish diet patterns, allowing for a more accurate assessment of trophic interactions [37]. The sampling area covered sites between latitudes 22° 18′ 96ʺ and 22° 16′ 66ʺ and longitudes 114° 08′ 16ʺ to 114° 18′ 41ʺ.

A commercial shrimp trawler with a 15 m outrigger was used to tow 4–8 nets (net width: 2 m; stretched mesh size: 2 cm) along the sea floor. For each of the eight sites, two transects were trawled during daylight at a speed of 6 km/h for 10 minutes, creating a total swept area of 0.22 km² (ranging from 0.008 to 0.016 km²). Water depths across the sites ranged from 6 to 39 m (S1 Table). To assess water quality, temperature and salinity data were retrieved from the nearest monitoring stations for the sampling period (April to May 2022) and averaged to represent mean conditions. During this time, water temperature ranged from 22 to 26 °C and salinity from 29 to 34 psu.

All marine organisms (fish and marine invertebrates) upon removal from the water immediately underwent rapid sorting, were placed into labelled zip-closure plastic bags, and stored on ice on board. No physical stunning was applied prior to placement on ice. The time from removal from water to placement on ice was under ten minutes to minimize potential suffering. Euthanasia was achieved by freezing on ice. Death was confirmed on the vessel by the absence of gill movement, absence of ocular reflex, and absence of any body movement. They were then transported to the laboratory and frozen at −20°C. Identification and measurement of all organisms were completed within 48 hours to avoid altering stable isotope signatures [38].

Potential fish prey species were selected based on their co-occurrence with predators, abundance in the catch, and suitability within the expected prey size range. Benthic autotrophs, such as macroalgae and microalgae, were excluded as potential prey items due to the limitations of the bottom trawl net’s mesh size, which was unsuitable for sampling them.

### Morphometric measurements for fish specimens and potential prey items

Fish specimens and their potential prey items were identified to the lowest possible taxonomic level based on morphological characteristics, using reference materials such as field guides, scientific literature, and online databases. These included “Fishes of Southern Taiwan”, “Marine fish fauna in Hong Kong Waters” [39], “The Brachyuran Crabs (Crustacea: Decapoda) of Hong Kong: a Historical Review and Catalogue” [40], the Hong Kong Register of Marine Species (https://www.marinespecies.org/hkrms/), and the Hong Kong Fish Net (https://www.hk-fish.net/). The geographic distribution of taxa was reviewed using FishBase (https://www.fishbase.se) and SeaLifeBase (https://www.sealifebase.ca/). Scientific names were verified through WoRMS (https://www.marinespecies.org/), and conservation status was assessed based on the IUCN Red List (https://www.iucnredlist.org/).

Each specimen was measured to the nearest 0.1 cm and weighed to the nearest 0.1 g with an electronic balance. Fish specimens were measured for total length (TL) and standard length (SL), while ray specimens were measured for total length (TL) and disc width (DW). For marine invertebrates, including stomatopod, bivalve, and gastropod, total length (TL) was recorded, while decapod was measured for carapace length (CL) and cephalopod for mantle length (ML). Any observable wounds, such as tail or claw loss, were recorded.

### Stable isotope analysis

**Fig 2:**
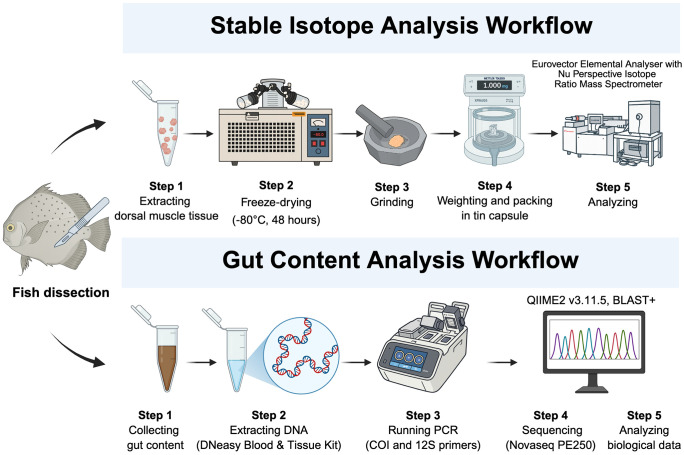
Workflows of stable isotope analysis and gut content analysis. Created with BioRender.com, under a CC BY license, with permission from BioRender, original copyright 2025.

Stable isotope analysis and gut content analysis workflows are shown in Fig 2. Stable carbon and nitrogen isotopes are commonly used to estimate predators’ long‐term dietary patterns [29]. Stable carbon isotopes identify major food sources, while stable nitrogen isotopes indicate the trophic level of consumers within the food web. These isotopes are expressed in delta notation (‰) relative to international standards. Approximately 0.5 g of dorsal muscle tissue above the lateral line from ﬁsh, abdominal muscle tissue from crustaceans, and foot muscle tissues from gastropods and bivalves were extracted and stored at ‐20°C. Fish (except the dissected specimens) and marine invertebrates were grouped into broad taxonomic categories at the order level.

All tissue samples were freeze‐dried at ‐80°C for at least 48 hours, then ground into homogeneous powder using a mortar and pestle before analysis. Samples were weighed to approximately 1 mg and placed in tin capsules for stable carbon (δ^13^C) and nitrogen (δ^15^N) isotope analysis using a Eurovector Elemental Analyser (Isomass Scientific Inc., Alberta, Canada) coupled with a Nu Perspective Isotope Ratio Mass Spectrometer (Nu Instruments Ltd., Wrexham, U.K.). According to Post et al. [41], a mathematical normalization technique was applied to avoid errors introduced by more negative stable carbon isotope values caused by lipid content accumulation in muscle tissue when the C:N ratio was equal to or larger than 3.5:


$$\delta^{\text{13}}{\text{C}}_{\text{normalized}}=\;\delta^{\text{13}}{\text{C}}_{\text{untreated}}-\;\text{3}.\text{32 }+\;\text{0}.\text{99 }\times \text{ C}:\text{N}$$

To estimate the isotopic niche sizes and overlaps of fish specimens, corrected standard ellipse areas (SEAc) and Bayesian standard ellipse areas (SEAb) were calculated using the package ‘SIBER’ in R *via* RStudio [42].

### Gut content analysis

Predators were dissected, and the gastrointestinal tract was removed from the fish’s body with sterile forceps and a scalpel. Gut content samples were transferred to sterile centrifuge tubes and immediately frozen at −80°C to avoid contamination and degradation until further processing. Prior to DNA extraction, frozen samples were thawed on ice, mixed manually, and large fragments of partially digested tissue were broken apart with a sterile hand-held homogenizer and vortexed to ensure representative subsampling. Approximately 100 mg of each homogenate was subsampled for DNA extraction.

DNA extraction on gut content samples was carried out using the DNeasy Blood & Tissue Kit (Qiagen, Germany) following the manufacturer’s protocol. PCR was be conducted using two primers sets, including 313 bp fragment of the mitochondrial cytochrome c oxidase subunit I (COI) region with primer pairs of m1COIintF (5′‐GGWACWGGWTGAACWGTWTAYCCYCC-3’) and jgHCO2198 (5′‐ TAIACYTCIGGRTGICCRAARAAYCA‐3’), and ~172 bp fragment of the mitochondrial 12S rRNA gene with MiFish-U-F (5′‐GTCGGTAAAACTCGTGCCAGC-3’) and MiFish-U-R (5′‐ CATAGTGGGGTATCTAATCCCAGTTTG‐3’). The DNA and PCR quality and quantity were examined using agarose gel electrophoresis. The successful reactions were then adjusted to the same concentration, pooled (20 samples per run), and sent to a sequencing service provider (Novogene HK Company Limited, Wan Chai, Hong Kong) for library preparation and Novaseq PE250 sequencing.

Raw reads were demultiplexed and trimmed of specific primers using Cutadapt v4.9, generating clean reads. These were further trimmed using the q2-dada2 plugin in QIIME2 v3.11.5 for quality filtering and removing low-quality or chimeric sequences. The remaining fragments were grouped into amplicon sequence variants (ASVs) using q2-dada2 for taxonomical classification. ASVs classified as class Actinopterygii were further identified to higher taxonomic levels using BLAST + . The CRABS software package was used to construct primer-specific reference databases. The COI rRNA sequences for groups of vertebrates and invertebrates, 12S rRNA sequences for phylum Chordata, and taxonomic information for both genes were downloaded from the NCBI GenBank database.

Primer-specific sequences were extracted using in silico PCR analysis and pairwise global alignment in CRABS. Extracted reads were dereplicated, assigned taxonomy, and used to train the Qiime2 naïve-Bayes classifier. Non-dietary taxa and those with low taxonomic resolution were discarded from the dataset. Sequences matching the DNA of the predator from which gut content was collected were also discarded. The geographic distribution of taxa was checked using FishBase (https://www.fishbase.se), SeaLifeBase (https://www.sealifebase.ca/), the Hong Kong Biodiversity Information Hub (https://bih.gov.hk/), and the Hong Kong Register of Marine Species (https://www.marinespecies.org/hkrms/). Scientific names were verified using WoRMS (https://www.marinespecies.org/). Conservation status was determined according to the IUCN Red List (https://www.iucnredlist.org/).

### Multivariate analysis

A dendrogram was used to visualize the diet dissimilarity of 16 fish species. A bipartite network plot was used to illustrate the trophic interactions between 16 fish species and their prey items using relative read abundance. A bubble plot was used to summarize the relative abundance of each prey species found in the gut contents of 16 fish species. A two-dimensional non-metric multi-dimensional scaling (NMDS) ordination plot visualized the Bray-Curtis distances among prey items in the gut contents of 16 fish species. This technique is robust and insensitive to outliers. A one-way analysis of similarity (ANOSIM) was used to test the null hypothesis that there were no differences in the diet composition between 16 fish species. Global *R* and *p* values statistically determined the similarity of prey assemblages of 16 fish species. Similarity percentage (SIMPER) analysis determined the percentage contribution of each food item to the dietary dissimilarity of 16 fish species. Overall average dissimilarity, average abundance, average dissimilarity, and cumulative contribution were calculated to identify typifying and discriminating prey of 16 fish species. The dissimilarity matrix, dendrogram, NMDS, ANOSIM, and SIMPER analyses were constructed and performed using the community ecology package ‘VEGAN’ in R *via* RStudio. All analyses were run with 9999 permutations based on the Bray-Curtis dissimilarity matrix. The bipartite network plot was constructed using the *plotweb* function in the package ‘BIPARTITE’ in R *via* RStudio.

## Results

### Characterization of fish specimens and morphometric analysis

Sixteen fish species were sampled from the southern and southwestern waters of Hong Kong (S1 Fig). For each species, we examined dietary habits using gut content metabarcoding to determine prey composition and assessed trophic positions through stable carbon and nitrogen isotope analysis of muscle tissue (Table 1). These fish species were abundant in our trawling survey and collectively span distinct vertical zones of the demersal community, including epibenthic and hyperbenthic. The epibenthic group included ten species: *Callionymus curvicornis*, *Gymnura japonica*, *Telatrygon zugei*, *Grammoplites scaber*, *Inegocia japonica*, *Lepidotrigla alata*, *Platycephalus indicus*, *Trachicephalus uranoscopus*, *Cynoglossus oligolepis*, and *Pseudorhombus cinnamoneus*. Meanwhile, the hyperbenthic group included six species: *Drepane punctata*, *Photopectoralis bindus*, *Siganus fuscescens*, *Dendrophysa russelii*, *Gerres japonicus*, and *Takifugu bimaculatus*. The mean total length (TL) (± S.D.) for the species ranged from 9.23 ± 1.47 cm to 48.38 ± 10.06 cm (Table 2). Mean standard length (SL) (± S.D.) ranged from 7.33 ± 1.26 cm to 26.63 ± 4.58 cm, while mean weight (± S.D.) ranged from 8.57 ± 4.70 g to 768.09 ± 621.60 g. Also, two species, the Japanese butterfly ray (*G. japonica*) and pale-edged stingray (*T. zugei*), were classified as vulnerable on the International Union for Conservation of Nature (IUCN) Red List [43,44].

**Table 1:** Sample size (number) of fish specimens.

Order	Species	Number	Number*
Acanthuriformes	*Drepane punctata*	5	4
	*Photopectoralis bindus*	5	7
	*Siganus fuscescens*	5	0
Callionymiformes	*Callionymus curvicornis*	5	11
Eupercaria *incertae sedis*	*Dendrophysa russelii*	5	16
	*Gerres japonicus*	5	1
Gobiiformes	*Acentrogobius caninus*	2	0
Myliobatiformes	*Gymnura japonica*	5	0
	*Telatrygon zugei*	5	7
Perciformes	*Grammoplites scaber*	4	2
	*Inegocia japonica*	5	25
	*Lepidotrigla alata*	3	2
	*Platycephalus indicus*	5	5
	*Trachicephalus uranoscopus*	5	13
Pleuronectiformes	*Cynoglossus oligolepis*	3	17
	*Pseudorhombus cinnamoneus*	5	25
Tetraodontiformes	*Takifugu bimaculatus*	5	2

^a^Numbers marked with an asterisk (*) were exclusively used for isotope analysis.

**Table 2:** The total length (TL), standard length (SL) or disc width (DW), and weight (mean ± S.D. [maximum, minimum]) of 16 fish species.

	*C.* *curvicornis*	*G.* *japonica*	*T.* *zugei*	*G.* *scaber*	*I.* *japonica*	*L.* *alata*	*P.* *indicus*	*T.* *uranoscopus*	*C.* *oligolepis*	*P.* *cinnamoneus*	*D.* *punctata*	*P.* *bindus*	*S.* *fuscescens*	*D.* *russelii*	*G.* *japonicus*	*T.* *bimaculatus*
TL (cm)	10.76 ± 2.30 [15.50, 6.80]	35.22 ± 10.92 [49.20, 21.40]	48.38 ± 10.06 [62.80, 31.00]	21.60 ± 8.53 [38.50, 15.00]	18.80 ± 3.20 [25.50, 9.40]	11.22 ± 3.20 [15.50, 7.60]	29.73 ± 5.01 [37.00, 20.50]	9.58 ± 1.05 [11.80, 8.00]	21.24 ± 6.00 [39.00, 11.80]	13.28 ± 5.61 [25.20, 5.50]	21.74 ± 2.11 [25.60, 18.90]	9.23 ± 1.47 [11.00, 7.00]	13.98 ± 1.09 [14.90, 12.40]	14.48 ± 1.42 [17.00, 10.60]	12.35 ± 0.82 [13.90, 11.70]	25.59 ± 5.15 [29.00, 14.30]
SL or DW (cm)	8.52 ± 1.71 [11.80, 5.50]	22.42 ± 6.59 [30.80, 13.60]	20.60 ± 4.20 [26.20, 14.00]	19.05 ± 8.15 [35.00, 12.00]	16.09 ± 2.72 [22.50, 8.00]	9.24 ± 2.65 [12.70, 6.20]	26.63 ± 4.58 [33.00, 17.90]	7.76 ± 0.86 [9.30, 6.60]	19.79 ± 5.56 [36.50, 11.00]	11.56 ± 4.88 [21.50, 6.50]	17.74 ± 1.73 [21.20, 15.50]	7.33 ± 1.26 [8.90, 5.50]	11.98 ± 0.53 [12.50, 11.10]	11.96 ± 1.33 [14.20, 8.50]	9.93 ± 0.68 [11.00, 9.10]	21.43 ± 4.64 [24.70, 11.30]
Weight (g)	8.57 ± 4.70 [17.45, 1.69]	768.09 ± 621.60 [1702.50, 132.43]	188.55 ± 104.09 [355.19, 65.17]	111.12 ± 179.86 [477.53, 24.96]	49.00 ± 23.83 [114.02, 6.03]	19.97 ± 14.53 [42.21, 6.03]	174.88 ± 84.56 [304.84, 45.38]	16.40 ± 6.69 [31.72, 8.74]	63.93 ± 71.62 [341.29, 6.86]	38.49 ± 46.12 [134.66, 4.00]	304.48 ± 65.38 [398.76, 193.72]	11.27 ± 6.16 [21.80, 3.75]	46.12 ± 10.55 [56.00, 29.70]	37.49 ± 11.53 [63.39, 12.62]	36.31 ± 7.48 [49.59, 30.01]	406.39 ± 168.27 [562.20, 76.43]

### Analysis of trophic niches and niche overlap via stable isotopes

**Fig 3:**
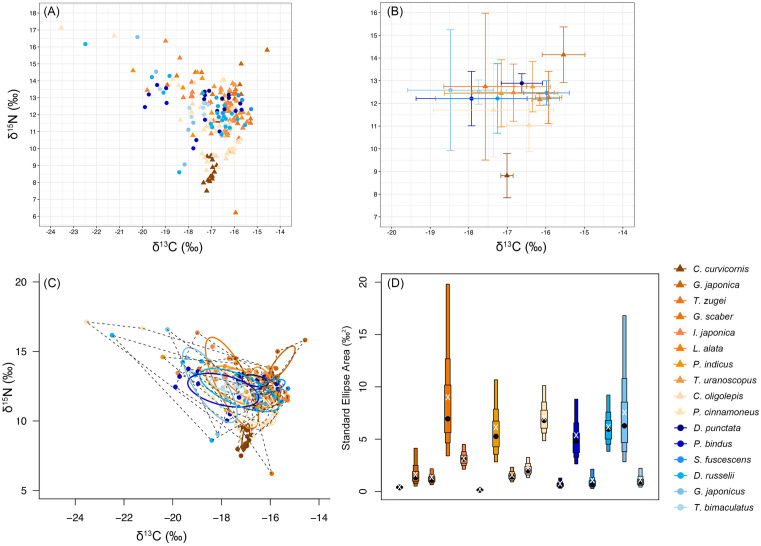
Bivariate isotope plots for 16 fish species illustrating (A) δ¹³C and δ¹^5^N values, and (B) the mean and standard deviation (± S.D.) of these values. (C) Biplot of corrected standard ellipse area (SEAc) and (D) density box plot of Bayesian standard ellipse area (SEAb) depicting the isotopic niche sizes for different fish species. Solid lines show standard ellipse area (40% of the data) and dashed lines show total convex hull area (100% of the data). Coloured boxes represent 50%, 75%, and 95% credible intervals, while central black dots and white ‘X’s represent modes and the SEAc point, respectively.

The mean stable carbon isotope values (± S.D.) for the 16 fish species were: −17.01 ± 0.16 ‰ to −17.74 ± 0.73 ‰ (Tables 3 and S2). The mean stable nitrogen isotope values (± S.D.) ranged from 8.82 ± 0.98 ‰ to 12.50 ± 0.54 ‰. Based on the bivariate isotope plots, the stable carbon isotope values were most enriched in *G. japonica*, but most depleted in *G. japonicus* (Fig 3A and 3B). The stable nitrogen isotope values were most enriched in *G. japonica*, but most depleted in *C. curvicornis*. Furthermore, the mean C:N ratios ranged from 2.70 to 4.05.

**Table 3:** Stable isotope values (Mean ± S.D. [Max, Min]) and trophic niche metrics of 16 fish species.

	*C.* *curvicornis*	*G.* *japonica*	*T.* *zugei*	*G.* *scaber*	*I.* *japonica*	*L.* *alata*	*P.* *indicus*	*T.* *uranoscopus*	*C.* *oligolepis*	*P.* *cinnamoneus*	*D.* *punctata*	*P.* *bindus*	*S.* *fuscescens*	*D.* *russelii*	*G.* *japonicus*	*T.* *bimaculatus*
δ^13^C (‰)	−17.01 ± 0.16 [−16.72, −17.34]	−15.54 ± 0.56 [−14.57, −15.98]	−15.93 ± 0.35 [−15.29, −16.36]	−17.57 ± 1.07 [−15.94, −18.92]	−16.84 ± 1.06 [−15.45, −19.79]	−16.15 ± 0.21 [−15.91, −16.47]	−17.16 ± 1.47 [−15.67, −20.41]	−16.34 ± 0.46 [−15.39, −17.60]	−16.44 ± 0.58 [−15.70, −17.82]	−17.36 ± 1.56 [−15.79, −23.54]	−16.62 ± 0.53 [−15.74, −17.31]	−17.93 ± 1.44 [−15.68, −19.89]	−15.98 ± 0.59 [−15.27, −16.67]	−17.26 ± 1.61 [−15.53, −22.49]	−18.47 ± 1.11 [−17.28, −20.22]	−17.74 ± 0.73 [−17.14, −19.24]
δ^15^N (‰)	8.82 ± 0.98 [11.63, 7.50]	14.15 ± 1.23 [15.82, 12.93]	12.27 ± 1.15 [13.48, 10.60]	12.74 ± 3.24 [14.50, 6.21]	12.48 ± 1.26 [16.35, 10.76]	12.17 ± 0.25 [12.44, 11.87]	12.45 ± 1.48 [14.60, 9.84]	12.74 ± 1.11 [14.49, 10.76]	11.04 ± 1.17 [12.79, 9.40]	11.71 ± 2.06 [17.13, 8.71]	12.89 ± 0.42 [13.41, 12.22]	12.21 ± 1.20 [13.75, 10.02]	12.46 ± 0.53 [13.14, 11.86]	12.22 ± 1.53 [16.17, 8.60]	12.58 ± 2.66 [16.58, 9.06]	12.50 ± 0.54 [13.51, 11.87]
Carbon (%)	44.04 ± 3.08 [55.06, 41.11]	43.39 ± 2.55 [46.03, 40.09]	42.96 ± 1.18 [44.53, 40.46]	45.05 ± 2.21 [47.89, 41.38]	45.65 ± 0.85 [46.64, 42.25]	43.28 ± 1.20 [44.61, 42.10]	45.48 ± 0.91 [46.65, 43.74]	43.97 ± 0.64 [45.29, 42.90]	44.63 ± 3.50 [56.01, 35.34]	43.80 ± 2.35 [46.25, 35.46]	45.69 ± 1.59 [48.85, 43.70]	44.86 ± 2.60 [49.86, 41.71]	45.40 ± 1.99 [48.01, 43.39]	45.68 ± 1.51 [48.19, 43.11]	48.75 ± 2.16 [51.52, 46.20]	44.88 ± 0.50 [45.74, 44.14]
Nitrogen (%)	13.87 ± 0.99 [17.42, 12.92]	15.68 ± 0.35 [16.12, 15.30]	15.93 ± 0.49 [16.53, 14.93]	13.57 ± 1.56 [14.56, 10.42]	14.17 ± 0.34 [14.77, 13.13]	13.52 ± 0.27 [13.85, 13.23]	14.10 ± 0.25 [14.48, 13.71]	13.58 ± 0.18 [13.90, 13.23]	13.82 ± 1.01 [16.89, 10.80]	13.41 ± 0.69 [14.18, 11.01]	13.22 ± 0.59 [13.68, 11.77]	12.92 ± 2.60 [49.86, 41.71]	13.92 ± 1.03 [14.99, 12.57]	13.26 ± 0.28 [13.71, 12.74]	12.14 ± 0.93 [13.30, 11.12]	13.87 ± 0.12 [14.06, 13.72]
C:N value	3.18 ± 0.04 [3.24, 3.12]	2.77 ± 0.17 [3.00, 2.62]	2.70 ± 0.04 [2.76, 2.60]	3.35 ± 0.31 [3.97, 3.13]	3.22 ± 0.05 [3.30, 3.12]	3.20 ± 0.03 [3.24, 3.18]	3.23 ± 0.04 [3.28, 3.18]	3.24 ± 0.04 [3.35, 3.18]	3.23 ± 0.06 [3.32, 3.12]	3.26 ± 0.05 [3.36, 3.17]	3.47 ± 0.28 [4.15, 3.28]	3.51 ± 0.48 [4.43, 3.07]	3.27 ± 0.12 [3.45, 3.18]	3.45 ± 0.16 [3.78, 3.25]	4.05 ± 0.46 [4.59, 3.47]	3.23 ± 0.03 [3.28, 3.20]
TA (‰^2^)	1.17	1.27	1.97	8.04	11.58	0.15	10.71	3.81	5.78	25.52	1.11	8.48	0.70	19.65	7.04	1.25
SEAb (‰^2^)	0.39	1.21	1.22	7.20	3.04	0.16	5.44	1.48	2.14	6.79	0.65	4.89	0.79	5.86	6.03	0.93
SEAc (‰^2^)	0.42	1.61	1.35	9.00	3.15	0.21	6.12	1.57	2.26	7.04	0.75	5.38	1.05	6.17	7.54	1.12

The trophic niche metrics for *P. cinnamoneus* and *D. russelii*—total area (TA), Bayesian standard ellipse area (SEAb), and corrected standard ellipse area (SEAc)—were notably higher than those of other species, with values of 25.52‰², 6.79‰², and 7.04‰² for *P. cinnamoneus*, and 19.65‰², 5.86‰², and 6.17‰² for *D. russelii*. This suggests that these species have highly varied diets and broader dietary niches (Fig 3C and 3D).

The isotopic niche overlap ranged from 0.00% to 72.91%, with the highest overlap occurring between *P. indicus* and *D. russelii* (Table 4). Following this, *P. cinnamoneus* and *D. russelii* showed 55.30% overlap, while *P. bindus* showed 53.04% overlap with the same species. Among all the comparisons, over one-third (42 out of 120) of them exhibited no overlap. The majority of zero-overlap involved *C. curvicornis*, which only overlapped with *G. scaber* (1.00%). Moreover, *G. japonica* had no overlap with nine other species, *L. alata* with five, *T. zugei* with four, and *C. oligolepis* with three. *Trachicephalus uranoscopus*, *D. punctata*, and *S. fuscescens* also showed no overlap with two other species, and between *G. scaber* and *L. alata*.

**Table 4:** Percentage of isotope niche overlap of 16 fish species.

	*C.* *curvicornis*	*G.* *japonica*	*T.* *zugei*	*G.* *scaber*	*I.* *japonica*	*L.* *alata*	*P.* *indicus*	*T.* *uranoscopus*	*C.* *oligolepis*	*P.* *cinnamoneus*	*D.* *punctata*	*P.* *bindus*	*S.* *fuscescens*	*D.* *russelii*	*G.* *japonicus*	*T.* *bimaculatus*
*C.* *curvicornis*	‒															
*G.* *japonica*	0.00	‒														
*T.* *zugei*	0.00	9.58	‒													
*G.* *scaber*	1.00	0.00	0.00	‒												
*I.* *japonica*	0.00	0.00	12.91	17.84	‒											
*L.* *alata*	0.00	0.00	12.07	0.00	6.37	‒										
*P.* *indicus*	0.00	0.03	11.71	35.97	51.57	3.43	‒									
*T.* *uranoscopus*	0.00	4.08	24.44	0.08	23.48	13.37	15.34	‒								
*C.* *oligolepis*	0.00	0.00	7.69	10.61	17.03	3.47	17.63	7.65	‒							
*P.* *cinnamoneus*	0.00	0.00	0.06	42.98	17.43	0.00	40.56	0.70	25.41	‒						
*D.* *punctata*	0.00	1.10	5.20	2.22	18.27	0.00	10.87	28.64	0.00	0.19	‒					
*P.* *bindus*	0.00	0.00	0.00	29.37	18.16	0.00	37.20	0.78	7.26	53.04	0.50	‒				
*S.* *fuscescens*	0.00	2.61	34.84	0.11	12.83	19.98	8.58	29.47	5.97	0.75	6.77	1.00	‒			
*D.* *russelii*	0.00	0.00	7.86	35.23	41.07	2.80	**72.91**	9.65	21.94	55.30	6.11	48.31	5.87	‒		
*G.* *japonicus*	0.00	0.00	0.00	17.40	0.36	0.00	13.81	0.00	0.00	27.02	0.00	35.90	0.00	18.95	‒	
*T.* *bimaculatus*	0.00	0.00	0.00	11.64	10.96	0.00	18.24	0.00	0.00	15.85	0.00	20.75	0.00	18.07	8.26	‒

Note: Dash (‒) means not applicable.

### Survey results: Community composition and marine food web dynamics

During the trawling survey, alongside specimens from the 16 fish species, a variety of potential prey items were collected, including smaller fish and invertebrates. Dissection and gut content metabarcoding were not performed on these smaller fish due to technical constraints. Decapoda and fish were the predominant groups, constituting 41.93% and 35.53% of the total catch by number of individuals, respectively. The Decapoda catch included at least 85 species from 46 genera and 23 families, while the fish catch comprised 109 species from 68 genera and 39 families, none of which were migratory. Other catches included barnacles (0.09%), bivalves (1.59%), brittle stars (0.65%), cephalopods (0.53%), feather stars (0.01%), gastropods (6.36%), polychaetes (0.14%), sea anemones (0.34%), sea cucumbers (1.06%), sea pens (7.73%), sea urchins (0.08%), corals (0.19%), starfish (0.01%), stomatopods (3.74%), and tunicates (0.03%).

Thirty invertebrate and 42 fish samples were selected and analyzed for stable isotope analysis (S1 Fig; S3 Table). These samples were classified into various groups, including ten species of Crustaceans (nine Decapoda species and one Stomatopoda species), at least 16 fish species, and at least eight species of mollusks (at least four Bivalvia species, one Cephalopoda species, and at least three Gastropoda species) (Table 5).

**Table 5:** Stable isotope values (Mean ± S.D., [Max, Min]) of potential prey items.

	Crustacean (Decapoda, Stomatopoda)	Small fish	Mollusk (Bivalvia, Cephalopoda, Gastropoda)
Sample size	16	42	14
δ^13^C (‰)	−17.09 ± 2.28 [-14.98, -24.14]	−17.14 ± 1.83 [-15.40, -24.84]	−15.49 ± 0.49 [-14.49, -16.26]
δ^15^N (‰)	10.40 ± 2.26 [14.01, 6.80]	12.48 ± 1.95 [18.88, 5.98]	8.59 ± 2.42 [11.80, 3.90]
Carbon (%)	40.97 ± 3.24 [45.63, 35.86]	43.08 ± 2.11 [47.34, 36.35]	41.05 ± 2.08 [43.99, 36.86]
Nitrogen (%)	12.61 ± 1.62 [14.85, 9.81]	13.19 ± 0.68 [14.86, 11.07]	11.29 ± 2.15 [13.89, 7.21]
C:N value	3.27 ± 0.19 [3.65, 3.00]	3.27 ± 0.14 [3.95, 3.03]	3.75 ± 0.68 [5.29, 3.17]

Among the potential food items analyzed, fish exhibited the lowest mean stable carbon isotope value (δ¹³C) at −17.14‰, whereas mollusks had the highest mean stable carbon isotope value at −15.49‰ (Tables 5 and S4). In terms of stable nitrogen isotope values (δ¹⁵N), mollusks recorded the lowest mean value at 8.59‰, while fish showed the highest mean value at 12.48‰ (Table 5).

**Fig 4:**
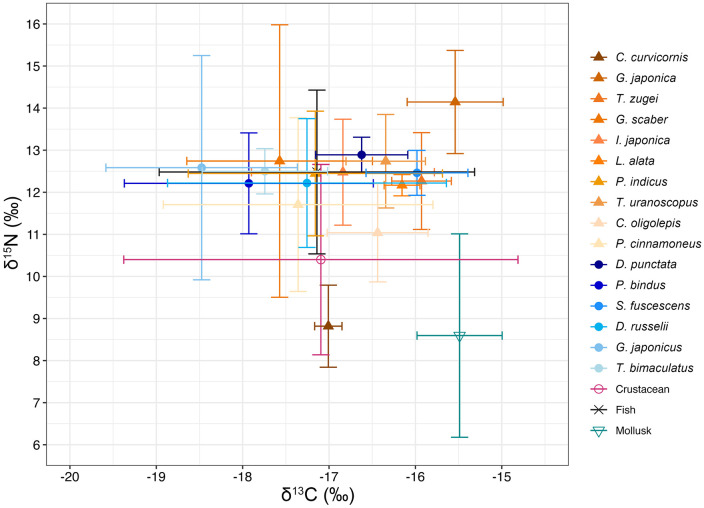
Bivariate isotope plot of δ¹³C and δ¹^5^N (mean ± S.D.) values for 16 fish species and other smaller fish and invertebrates as potential prey items.

The bivariate isotope plot illustrates the trophic structure of the local benthic food web, with most fish species (including the 16 fish species and smaller fish species) occupying higher positions than the marine invertebrates, including Crustacean and mollusk (Fig 4). This reflects their higher trophic level, driven by the enriched stable nitrogen isotope values. Notably, the fish species *C. curvicornis* occupies a significantly lower trophic position compared to all other fish species and crustaceans, with its level only slightly higher than that of mollusks.

The 16 fish species also exhibit horizontal partitioning in stable carbon isotope values, reflecting their dietary reliance on pelagic or benthic food sources. Seven species (including *G. scaber*, *P. indicus*, *P. cinnamoneus*, *P. bindus*, *D. russelii*, *G. japonicus*, and *T. bimaculatus*) are grouped on the left side of the plot, with lower mean stable carbon isotope values than that of the Crustacean (−17.09‰), suggesting their dietary reliance on more pelagic prey. The remaining nine species (including *C. curvicornis*, *G. japonica*, *T. zugei*, *I. japonica*, *L. alata*, *T. uranoscopus*, *C. oligolepis*, *D. punctata*, and *S. fuscescens*) are grouped on the right side, with higher mean stable carbon isotope values than that of the Crustacean but still lower than that of the mollusk (−15.49‰), indicating their dietary preference for more demersal prey.

### Dietary analysis using COI and 12S metabarcoding

**Fig 5:**
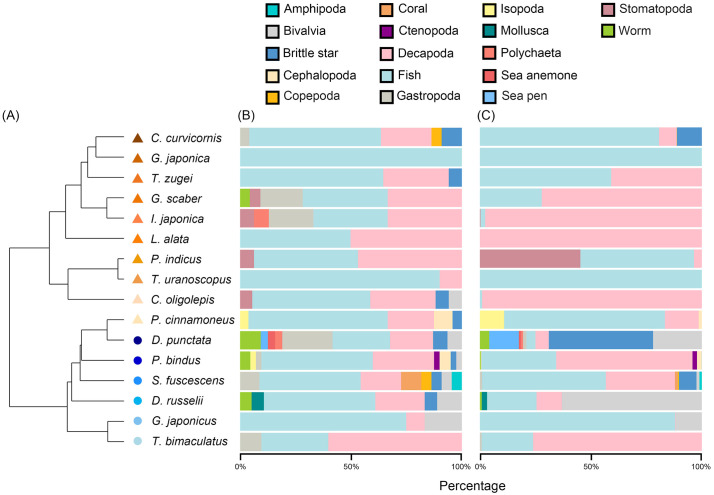
Cluster dendrogram of diet dissimilarity (Bray-Curtis index) (A) and stacked bar charts showing dietary compositions based on prey frequency of occurrence (B) and relative read abundance (C). Prey frequency of occurrence is calculated as (Number of samples with a specific prey group detected ÷ Total number of samples with prey detected) × 100. Relative read abundance is calculated as (Number of reads of a specific prey group ÷ Total number of reads of all prey groups) × 100.

**Fig 6:**
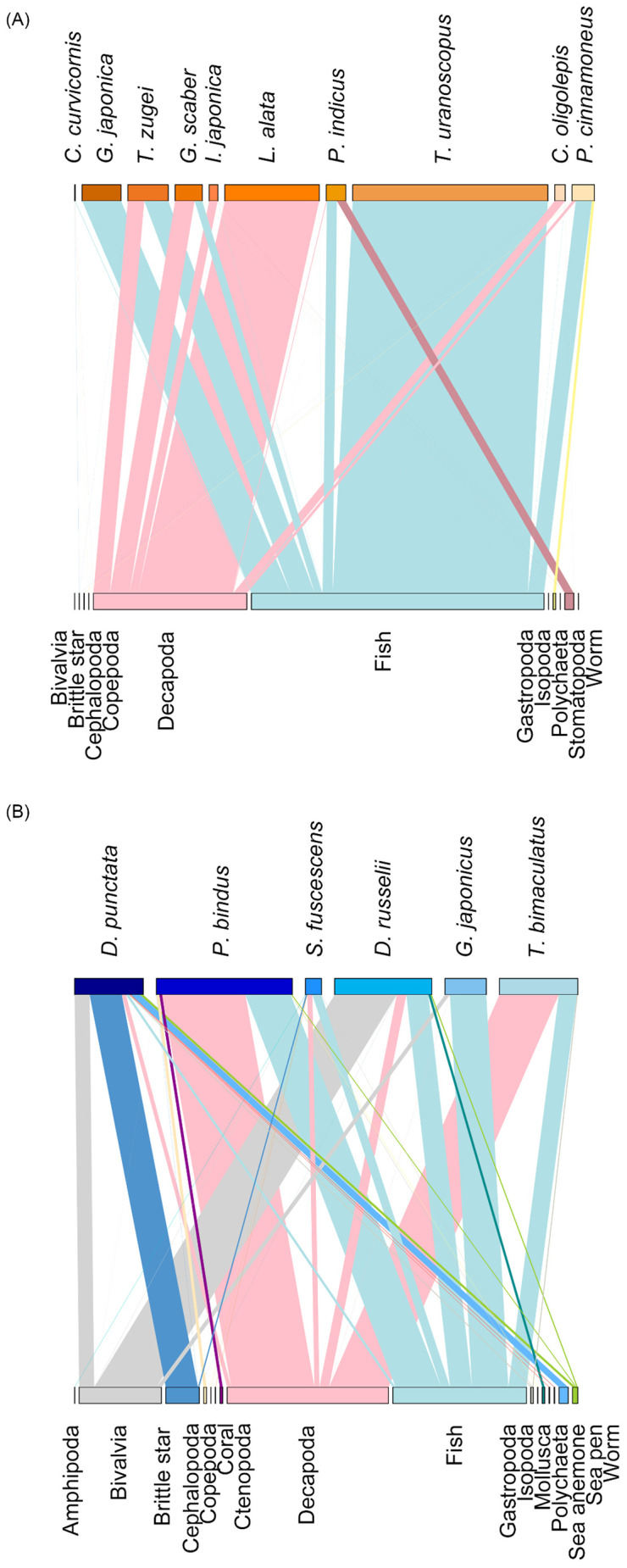
Bipartite network plots showing the trophic interactions between the (A) epibenthic fish community, (B) hyperbenthic fish community, and their prey items. The upper boxes represent the epibenthic and hyperbenthic fish species, and the lower boxes represent the prey items. The line thickness connecting prey to fish species represents the relative read abundance.

In the dendrogram, the fish species can be classified into two main clusters (Fig 5A). The first cluster includes all but one epibenthic fish, including *C. curvicornis*, *G. japonica*, *T. zugei*, *G. scaber*, *I. japonica*, *L. alata*, *P. indicus*, *T. uranoscopus*, and *C. oligolepis*. The second cluster includes most hyperbenthic fish, including *D. punctata*, *P. bindus*, *S. fuscescens*, *D. russelii*, *G. japonicus*, and *T. bimaculatus*, as well as one epibenthic fish *P. cinnamoneus*. Furthermore, the bipartite network plots visualize the predator-prey interactions based on the gut content analysis, illustrating a diverse diet for the fish species and their unique foraging preferences (Fig 6).

The COI and 12S barcoding diet analysis revealed that the 16 fish species have a diverse diet, with 17 prey groups identified (Fig 5). Particularly for fish and Decapoda, they can be found in all gut contents. Notably, *D. punctata* and *P. bindus* have the most highly diverse diet, in which nine groups were identified from their gut contents. By prey frequency of occurrence, the diet of *D. punctata* mainly consists of fish (25.81%), Gastropoda (22.58%), and Decapoda (19.35%), while that of *P. bindus* mainly consists of fish (50.00%) and Decapoda (27.50%) (Fig 5B). *Siganus fuscescens* has eight groups of prey, dominated by fish (45.45%) and Decapoda (18.18%), while *D. russelii* has six groups of prey, dominated by fish (50.00%) and Decapoda (22.22%). Five species (*C. curvicornis*, *G. scaber*, *I. japonica*, *C. oligolepis*, and *P. cinnamoneus*) show diversity of five groups of prey, with fish (ranging from 33.33% to 62.50%) and Decapoda (ranging from 20.83% to 29.41%) as major prey. Four species (*T. zugei*, *P. indicus*, *G. japonicus*, and *T. bimaculatus*) have three groups of prey, dominated by fish (ranging from 30.00% to 75.00%) and Decapoda (8.30% to 60.00%). *Lepidotrigla alata* and *T. uranoscopus* have a relatively less diverse diet, with only two groups of prey identified from their gut content, including Decapoda (50.00%, 10.00%) and fish (50.00%, 90.00%). *Gymnura japonica* has the least diverse diet as it consumed fish (100.00%) exclusively. With the exception of *T. bimaculatus*, which relied heavily on Decapoda as the major prey (60.00%), all other species predominantly relied on fish as their major prey, ranging from 25.81% in *D. punctata* to 90.00% in *T. uranoscopus*.

*P. bindus* has the highest prey richness (40 prey species), mainly consumed Decapoda (61.20%) and fish (33.45%) in terms of relative read abundance (Fig 5C). This was followed by *D. punctata* (31 prey species), which consumed brittle star (46.83%) and Bivalvia (21.88%), and *P. cinnamoneus* (24 prey species), which consumed fish (72.25%), Decapoda (15.16%), and Isopoda (11.19%). *Siganus fuscescens* and *C. curvicornis* have 22 prey species, in which the former consumed fish (55.56%), Decapoda (31.06%), and brittle star (7.89%), while the latter’s diet was dominated by fish (80.60%). *Grammoplites scaber* (20 prey species) consumed Decapoda (71.89%) and fish (28.04%), whereas *D. russelii* (18 prey species) consumed Bivalvia (62.76%) and fish (22.17%). *Telatrygon zugei* (17 prey species) consumed fish (59.31%) and Decapoda (40.65%), and *C. oligolepis* (16 prey species) was dominated by Decapoda (98.69%), with a minority of fish (1.17%). *Platycephalus indicus* and *I. japonica* have 15 prey species, in which the former consumed fish (51.03%) and Stomatopoda (45.45%), and the latter mainly consumed Decapoda (97.31%). *Gymnura japonica* (14 prey species) consumed fish (100%) exclusively. *Lepidotrigla alata* and *G. japonicus* have 12 prey species, in which the former primarily consumed Decapoda (99.92%) and fish (0.08%), while the latter primarily consumed fish (88.02%) and Bivalvia (11.75%). *Trachicephalus uranoscopus* and *T. bimaculatus* have ten prey species, in which the former was dominated by fish (almost 100.00%), with a minority of Decapoda (less than 0.01%), and the latter consumed Decapoda (75.77%) and fish (23.08%).

**Fig 7:**
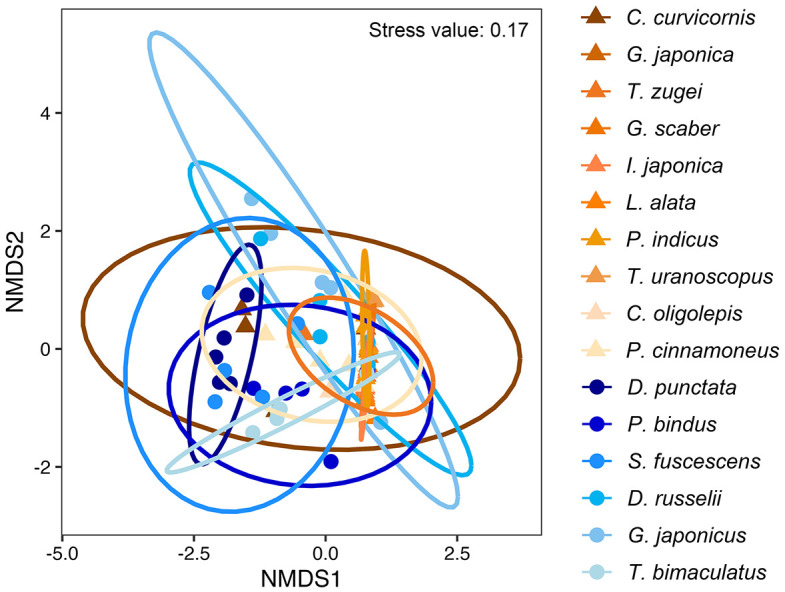
Two-dimensional non-metric multi-dimensional scaling (NMDS) ordination plots representing Bray-Curtis distances among prey items.

The NMDS plot showing minimal overlapping area between species was supported by SIMPER analysis, which identified an overall dietary dissimilarity ranging from 57.84% (between *P. bindus* and *T. bimaculatus*) to 99.98% (between *T. uranoscopus* and *D. punctata*) (Fig 7; Tables 6 and S5). The dissimilarities among most fish species were driven by Decapoda, which contributed 34.60% (between *D. punctata* and *T. bimaculatus*) to 99.80% (between *L. alata* and *S. fuscescens*). Fish is the secondary driver, which contributed 40.60% (between *C. curvicornis* and *D. punctata*) to 100.00% (between *T. uranoscopus* with *G. japonica*, *S. fuscescens*, and *G. japonicus*). Apart from Decapoda and fish, the dissimilarities between *D. punctata* with *S. fuscescens*, *D. russelii*, and *G. japonicus* were driven by brittle star (33.40%), Bivalvia (30.30%), and brittle star (31.60%), respectively.

**Table 6:** SIMPER analysis showing the dissimilarity percentage between 16 fish species.

	*C.* *curvicornis*	*G.* *japonica*	*T.* *zugei*	*G.* *scaber*	*I.* *japonica*	*L.* *alata*	*P.* *indicus*	*T.* *uranoscopus*	*C.* *oligolepis*	*P.* *cinnamoneus*	*D.* *punctata*	*P.* *bindus*	*S.* *fuscescens*	*D.* *russelii*	*G.* *japonicus*	*T.* *bimaculatus*
*C.* *curvicornis*	‒															
*G.* *japonica*	75.84	‒														
*T.* *zugei*	86.96	83.20	‒													
*G.* *scaber*	83.78	90.23	75.75	‒												
*I.* *japonica*	86.57	95.01	75.47	71.47	‒											
*L.* *alata*	99.31	99.53	79.34	72.64	77.43	‒										
*P.* *indicus*	92.77	80.41	80.00	89.29	90.10	97.21	‒									
*T.* *uranoscopus*	99.49	78.40	82.86	93.41	99.85	99.89	90.36	‒								
*C.* *oligolepis*	83.28	87.81	71.58	72.28	68.34	72.20	89.89	99.78	‒							
*P.* *cinnamoneus*	81.48	79.66	79.03	79.98	82.13	90.62	82.58	87.58	82.16	‒						
*D.* *punctata*	83.53	98.40	96.53	93.48	93.61	99.75	98.48	99.98	95.44	94.97	‒					
*P.* *bindus*	68.08	76.65	73.68	71.86	74.54	96.45	84.64	99.52	71.04	74.46	91.24	‒				
*S.* *fuscescens*	69.30	90.26	94.42	82.42	84.25	99.52	96.86	99.91	87.60	86.21	82.17	78.15	‒			
*D.* *russelii*	66.30	83.95	90.33	79.80	82.34	99.18	94.40	99.78	82.44	83.22	84.10	69.21	64.35	‒		
*G.* *japonicus*	82.23	83.77	92.01	95.34	96.75	99.76	95.78	99.63	92.06	92.08	93.32	83.82	91.90	87.80	‒	
*T.* *bimaculatus*	70.60	87.07	82.77	70.41	73.60	97.57	89.85	99.80	75.99	80.14	86.17	57.84	66.62	63.52	91.30	‒

Note: Dash (‒) means not applicable.

The NMDS plot yielded a stress value of 0.17, which is slightly elevated. This is likely influenced by the constraints of field sampling, which resulted in small and uneven sample sizes across species. While most species were represented by five individuals, sample sizes for *A. caninus*, *G. scaber*, *L. alata*, and *C. oligolepis* were smaller (n = 2–4). Such imbalances can reduce the stability of the ordination and increase stress independently of the true ecological dissimilarities. However, the overarching pattern of significant dietary segregation among the 16 species was robustly supported by ANOSIM, which revealed strong and significant global differences (Global R = 0.428, p < 0.001). This confirms that the observed dietary differences between species were substantially greater than any variation within species.

### Prey diversity and specific dietary patterns among fish species

**Fig 8:**
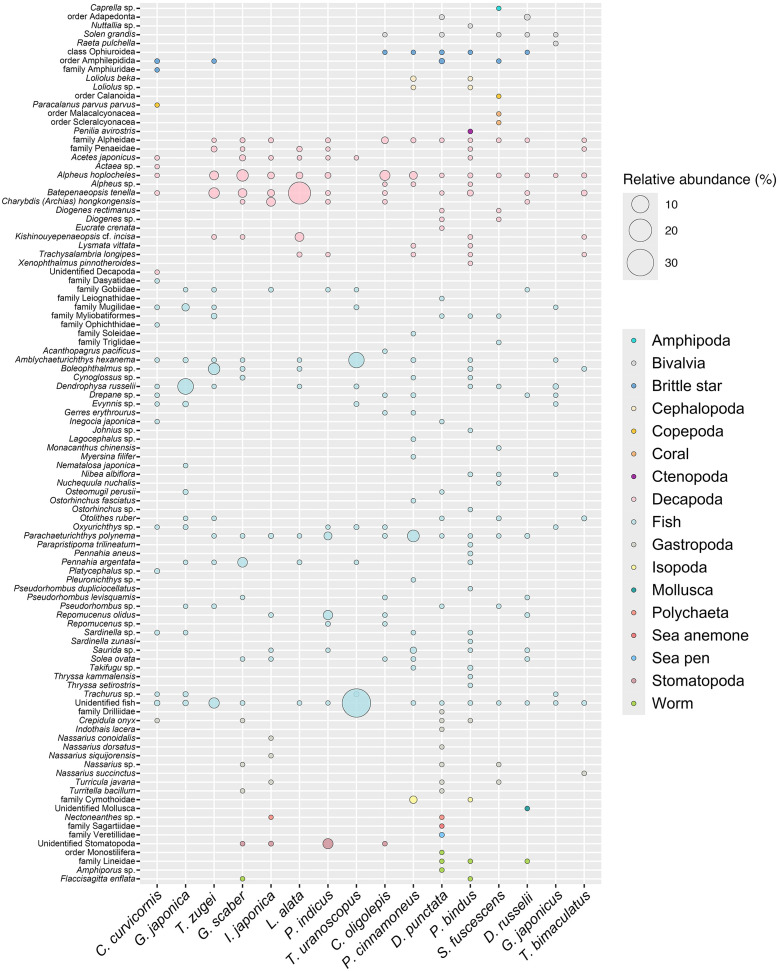
Bubble plot showing the relative abundance of prey items in gut content DNA.

Among those 17 prey groups, at least 100 species were found from the gut contents in the 16 fish species (one from Amphipoda, four from Bivalvia, three from brittle star, two from Cephalopoda, two from Copepoda, two from coral, one from Ctenopoda, at least 32 from Decapoda, at least 98 from fish, 20 from Gastropoda, one from Isopoda, at least one from Mollusca, one from polychaeta, one from sea anemone, one from sea pen, at least one from Stomatopoda, four from worm) (Fig 8). Except for the Hong Kong non-native gastropod *Crepidula onyx*, all of the prey items were native species in the South China Sea (including Hong Kong) marine area.

While fish constitute the primary component of the diet across the studied species, notable variations in prey preferences were observed, as reflected by differences in relative read abundance. For instance, *Platycephalus* sp. and *Takifugu* sp. serve as primary prey for *C. curvicornis* and *P. bindus*, respectively. *Dendrophysa russelii* is a critical food source for both *G. japonica* and *G. japonicus*, whereas *Boleophthalmus* sp. is particularly important for *T. zugei*, *L. alata*, and other species. *Pennahia argentata* and *Parachaeturichthys polynema* are also prominently featured in various diets (Table 7). Differences in Decapoda prey were also noted, with *Batepenaeopsis tenella* and *Acetes japonicus* being vital for several species (Table 8). *Alpheus hoplocheles* and other shrimp species also play important roles across multiple diets. Notably, *G. japonica* is exclusively piscivorous.

**Table 7:** Percentage of the three most abundant fish prey items in the diets of 16 fish species by relative read abundance.

	Fish prey species	Percentage
*C. curvicornis*	*Platycephalus* sp.	22.15
	*Sardinella* sp.	9.37
	family Mugilidae	3.84
*G. japonica*	*Dendrophysa russelii*	90.95
	family Mugilidae	5.93
	*Evynnis* sp.	1.01
*T. zugei*	*Boleophthalmus* sp.	57.16
	family Myliobatiformes	1.58
	*Amblychaeturichthys hexanema*	0.19
*G. scaber*	*Pennahia argentata*	98.11
	*Cynoglossus* sp.	0.83
	*Boleophthalmus* sp.	0.58
*I. japonica*	*Parachaeturichthys polynema*	64.79
	*Repomucenus olidus*	18.11
	family Gobiidae	14.69
*L. alata*	*Boleophthalmus* sp.	41.44
	*Pennahia argentata*	37.27
	*Parachaeturichthys polynema*	4.17
*P. indicus*	*Repomucenus olidus*	65.88
	*Parachaeturichthys polynema*	33.56
	*Repomucenus* sp.	0.51
*T. uranoscopus*	*Amblychaeturichthys hexanema*	17.31
	*Trachurus* sp.	0.08
	*Oxyurichthys* sp.	0.05
*C. oligolepis*	*Parachaeturichthys polynema*	25.76
	*Pseudorhombus levisquamis*	14.87
	*Repomucenus olidus*	12.88
*P. cinnamoneus*	*Parachaeturichthys polynema*	92.64
	*Saurida* sp.	6.06
	*Amblychaeturichthys hexanema*	0.34
*D. punctata*	*Otolithes ruber*	28.77
	family Leiognathidae	15.75
	family Myliobatiformes	13.01
*P. bindus*	*Takifugu* sp.	30.79
	*Boleophthalmus* sp.	17.67
	*Pennahia aneus*	9.74
*S. fuscescens*	*Otolithes ruber*	50.75
	family Triglidae	15.27
	*Monacanthus chinensis*	12.69
*D. russelii*	*Parachaeturichthys polynema*	32.60
	*Repomucenus olidus*	24.56
	*Solea ovata*	17.05
*G. japonicus*	*Dendrophysa russelii*	82.70
	*Oxyurichthys* sp.	4.43
	*Trachurus* sp.	4.22
*T. bimaculatus*	*Otolithes ruber*	50.90
	*Boleophthalmus* sp.	25.03

Note: The percentage is calculated within the fish prey group.

**Table 8:** Percentage of the three most abundant Decapoda prey items in the diets of 16 fish species by relative read abundance.

	Decapoda prey species	Percentage
*C. curvicornis*	*Batepenaeopsis tenella*	69.44
	*Acetes japonicus*	25.79
	*Alpheus hoplocheles*	1.59
*T. zugei*	*Batepenaeopsis tenella*	62.53
	*Alpheus hoplocheles*	33.63
	family Penaeidae	3.57
*G. scaber*	*Alpheus hoplocheles*	70.22
	*Batepenaeopsis tenella*	25.21
	*Acetes japonicus*	3.87
*I. japonica*	*Charybdis (Archias) hongkongensis*	62.97
	*Batepenaeopsis tenella*	19.41
	*Alpheus hoplocheles*	16.47
*L. alata*	*Batepenaeopsis tenella*	92.49
	*Kishinouyepenaeopsis* cf. *incisa*	5.66
	*Alpheus hoplocheles*	1.44
*P. indicus*	*Alpheus hoplocheles*	78.23
	family Alpheidae	9.68
	*Batepenaeopsis tenella*	8.00
*T. uranoscopus*	*Acetes japonicus*	100.00
*C. oligolepis*	*Alpheus hoplocheles*	84.41
	family Alpheidae	15.00
	*Batepenaeopsis tenella*	0.40
*P. cinnamoneus*	*Alpheus hoplocheles*	94.75
	family Alpheidae	3.62
	*Lysmata vittata*	0.94
*D. punctata*	family Alpheidae	28.84
	*Diogenes* sp.	27.91
	*Alpheus hoplocheles*	14.88
*P. bindus*	*Batepenaeopsis tenella*	82.30
	*Alpheus hoplocheles*	11.87
	*Kishinouyepenaeopsis* cf. *incisa*	3.25
*S. fuscescens*	family Alpheidae	55.38
	*Alpheus hoplocheles*	38.46
	*Diogenes* sp.	5.00
*D. russelii*	*Batepenaeopsis tenella*	40.76
	*Alpheus hoplocheles*	29.32
	*Charybdis (Archias) hongkongensis*	21.29
*G. japonicus*	*Alpheus hoplocheles*	100.00
*T. bimaculatus*	*Batepenaeopsis tenella*	80.56
	*Alpheus hoplocheles*	16.02
	*Kishinouyepenaeopsis* cf. *incisa*	1.55

Note: The percentage is calculated within the Decapoda prey group.

## Discussion

This study delves into the trophic ecology of 16 fish species co-occurring in the southern and southwestern waters of Hong Kong, a region characterized by its unique ecological dynamics and significant anthropogenic pressures. Through a comprehensive analysis of gut contents and stable carbon and nitrogen isotope values, our research highlights the critical role of dietary diversification in enabling the coexistence of demersal fish species. The findings demonstrate a variety of prey species and low trophic niche overlap among these species, underscoring the adaptive strategies that support ecological stability and resilience in this urbanized marine environment.

### Trophic structure and species composition in Hong Kong waters

The trawling survey reflected that the benthic marine ecosystem in the southern and southwestern waters of Hong Kong was dominated by high trophic level Decapoda and fish in the community, occupying over 77% of the total catch. The remaining catch includes diverse marine invertebrates, categorized into higher trophic level species, including cephalopods and stomatopods, and lower trophic level species, including barnacles, bivalves, brittle stars, corals, feather stars, gastropods, polychaetes, sea anemones, sea cucumbers, sea pens, sea urchins, starfish, and tunicates.

The 16 demersal fish species were from seven orders, including spotted sicklefish *Drepane punctata*, orangefin ponyfish *Photopectoralis bindus*, and mottled spinefoot *Siganus fuscescens* from order Acanthuriformes, horn dragonet *Callionymus curvicornis* from order Callionymiformes, goatee croaker *Dendrophysa russelii* and Japanese silver-biddy *Gerres japonicus* from order Eupercaria *incertae sedis*, Japanese butterflyray *Gymnura japonica* and pale-edged stingray *Telatrygon zugei* from order Myliobatiformes, rough flathead *Grammoplites scaber*, Japanese flathead *Inegocia japonica*, gurnard *Lepidotrigla alata*, bartail flathead *Platycephalus indicus*, and stargazing stonefish *Trachicephalus uranoscopus* from order Perciformes, tonguesole *Cynoglossus oligolepis* and cinnamon flounder *Pseudorhombus cinnamoneus* from order Pleuronectiformes, and pufferfish *Takifugu bimaculatus* from order Tetraodontiformes. They were classified into epibenthic and hyperbenthic fish communities, identified by morphological characteristics. Epibenthic fish have a flattened body, a mouth positioned upwards, and eyes on the same side or top of the head for contact with the seabed. Hyperbenthic fish feature a streamlined or compressed body with eyes on opposite sides, aiding their swimming against currents in the water column.

Therefore, ten fish species (*C. curvicornis*, *G. japonica*, *T. zugei*, *G. scaber*, *I. japonica*, *L. alata*, *P. indicus*, *T. uranoscopus*, *C. oligolepis*, and *P. cinnamoneus*) from the orders Callionymiformes, Myliobatiformes, Perciformes, and Pleuronectiformes were grouped as the epibenthic fish, in which their diet consist of mostly benthic prey species, with fish and Decapoda as the dominant prey (Figs 5 and 7). Six fish species (*D. punctata*, *P. bindus*, *S. fuscescens*, *D. russelii*, *G. japonicus*, and pufferfish *T. bimaculatus*) from the orders Acanthuriformes, Eupercaria *incertae sedis*, and Tetraodontiformes were grouped as the hyperbenthic fish, in which they consume a mixture of planktonic and benthic prey species, with Decapoda, fish, and Bivalvia as their major prey.

### Trophic niche partitioning and dietary strategies

In this study, resource partitioning through clear trophic niche segregation and distinct dietary compositions was observed among the 16 fish species, serving as a possible strategy to minimize interspecific competition. Stable isotope analysis, reflecting their long-term dietary pattern, showed no isotopic niche overlap over one-third of the species pairs (Table 4). Particularly for Japanese butterflyray (*G. japonica*), which exhibited a particularly high trophic position with high stable nitrogen isotope values, reflecting their dietary preference for higher trophic level organisms. This is corroborated by the gut content analysis, which was dominated by high trophic level fish prey, particularly goatee croaker (*Dendrophysa russelii*). While horn dragonet (*C. curvicornis*) exhibited a particularly low trophic position with low stable nitrogen isotope values, reflecting their dietary preference for lower trophic level prey, and its gut content consisted mainly of lower trophic level prey such as sardinella fish (*Sardinella* sp.), smoothshell shrimp (*Batepenaeopsis tenella*), and brittle star in the order Amphilepidida. The divergent trophic positions facilitate occupation of shared habitats, enabling niche divergence and resource partitioning among species. These findings were supported by the gut content analysis. It revealed that the 16 fish species are bottom-feeding carnivores due to the consumption of a large proportion of demersal fish and benthic invertebrates (mainly Decapoda). However, their primary prey sources differed among species, as supported by the high dietary dissimilarity ranging from over 57% to nearly 100% (Table 6). For example, flathead *Platycephalus* sp., silver croaker *Pennahia argentata*, Chinese darter dragonet *Repomucenus olidus*, and pufferfish *Takifugu* sp. are the sole primary fish prey for *C. curvicornis*, *G. scaber*, *P. indicus*, and *P. bindus*, respectively. The crab *Charybdis (Archias) hongkongensis* and Akiami paste shrimp *Acetes japonicus* are the sole primary Decapoda prey for *I. japonica* and *T. uranoscopus*, respectively.

Nonetheless, bartail flathead (*P. indicus*) and goatee croaker (*D. russelii*) showed a high isotope niche overlap of 72.91%, indicating similar long-term dietary habits, which is greater than other species comparisons (all below 55.30%). Gut content metabarcoding revealed they share Decapoda and fish prey, co-occurring in the same habitat. Shared Decapoda prey includes snapping shrimp (*Alpheus hoplocheles*), smoothshell shrimp (*Batepenaeopsis tenella*), and crab (*C. hongkongensis*). Shared fish prey includes goby (*Parachaeturichthys polynema*), Chinese darter dragonet (*R. olidus*), and lizardfish (*Saurida* sp.).

The integration of stable isotope and DNA metabarcoding techniques enables a robust analysis of trophic dynamics by bridging the temporal resolution gap between these methodologies. Stable isotope analysis quantifies long-term dietary assimilation through trophic niche metrics (e.g., SEAc and isotopic patterns), while DNA metabarcoding captures short-term prey composition at high taxonomic resolution. The isotopic data revealed clear resource partitioning and specialization among fish species, with minimal niche overlap (all below 55.30%, except for *P. indicus* and *D. russelii*). DNA metabarcoding further highlighted high dietary dissimilarity among species (57%–100%, Table 6), with *Platycephalus* sp., *P. argentata*, *R. olidus*, and *Takifugu* sp. identified as the dominant fish prey for *C. curvicornis*, *G. scaber*, *P. indicus*, and *P. bindus*, respectively. Similarly, *C. hongkongensis* and *A. japonicus* were the primary Decapoda prey for *I. japonica* and *T. uranoscopus*. These findings demonstrate how resource partitioning and dietary specialization reduce interspecific competition, revealing the ecological roles of prey taxa in structuring marine food webs and maintaining ecosystem stability.

### Dietary habits and predator roles in the ecosystem

Horn dragonet (*C. curvicornis*), Japanese butterflyray (*G. japonica*), pale-edged stingray (*T. zugei*), bartail flathead (*P. indicus*), stargazing stonefish (*T. uranoscopus*), cinnamon flounder (*P. cinnamoneus*), mottled spinefoot (*S. fuscescens*), and Japanese silver-biddy (*G. japonicus*) were considered as piscivores preying on fish (ranging from 51.03% to 100.00%) as their major target. Notably, Japanese butterflyray (*G. japonica*) showed an exclusively piscivorous diet, while stargazing stonefish (*T. uranoscopus*) showed nearly complete reliance on fish prey, with a small amount of Decapoda prey. Among all the fish prey species, the majority were classified as demersal, with a smaller proportion classified as fast-swimming pelagic fish, such as sardinella *Sardinella* sp. and mackerel *Trachurus* sp. Rough flathead (*G. scaber*), Japanese flathead (*I. japonica*), gurnard (*L. alata*), tonguesole (*C. oligolepis*), orangefin ponyfish (*P. bindus*), and pufferfish (*T. bimaculatus*) were considered as crustacean feeders as their diets include mostly Decapoda (ranging from 61.20% to 99.92%) rather than other organisms, including fish. Not only did these fish rely heavily on the shrimp, such as Akiami paste shrimp *A. japonicus* and snapping shrimp *A. hoplocheles*, they also relied on crab *C. hongkongensis* and hermit crab *Diogenes* sp. Besides, rather than relying on fish and Decapoda as prey, spotted sicklefish (*D. punctata*) and goatee croaker (*D. russelii*) relied on brittle stars from the order Amphilepidida and Bivalvia in the order Adapedonta as their major prey, respectively. Due to their slow-moving behaviour, these marine invertebrate prey required less foraging effort compared to other active prey. All fish species generally prefer consuming high-energy prey for growth and survival rather than low-quality gelatinous or planktonic organisms, which require less energetic effort for catching and are thus a common food source in marine organisms [45,46]. Planktonic prey species, including Amphipoda, Copepoda, and Ctenopoda, accounted for less than 3% of the diets of horn dragonet (*C. curvicornis*), orangefin ponyfish (*P. bindus*), and mottled spinefoot (*S. fuscescens*).

Therefore, the dietary preference of the 16 fish species underscores their role in controlling the benthic invertebrates and demersal fish communities, positioning them as medium-sized predators within the marine ecosystem. Meanwhile, changes in the abundance of these prey species could directly impact the dietary habits and survival of the fish species, indicating the significance of maintaining species diversity.

### Conservation concerns for vulnerable ray species

Among the 16 fish species, Japanese butterfly ray (*G. japonica*) and pale-edged stingray (*T. zugei*) were classified as vulnerable on the International Union for Conservation of Nature (IUCN) Red List [43,44], indicating higher extinction risks. For butterfly rays, their diets heavily rely on goatee croaker *D. russelii*, an economically significant species in the family Sciaenidae that faces overexploitation from intensive fishing in the local region. The high stable nitrogen isotope values (averaging 14.15‰) reflect their long-term consumption of high trophic level prey such as the adult goatee croaker, thus occupying the highest trophic position in the local food web. For stingrays, their diets heavily rely on the mudskipper *Boleophalmus* sp. and the smoothshell shrimp *B. tenella*, suggesting their possible vertical movement between coastal mudflat and deeper water for foraging, highlighting the importance of habitat connectivity. The relatively low stable nitrogen isotope values (averaging 12.27‰) compared to butterfly rays reflect their slightly reduced trophic level, due to the long-term consumption of lower trophic level prey such as Decapoda.

Although goatee croaker *D. russelii* was listed as least concern in the IUCN Red List, it still faces threats from overfishing, driven by the global reliance on Sciaenidae fish for livelihood. Global croaker capture increased steadily from the 1950s to the 2010s, peaking at 17,406 thousand tonnes in the 2010s [47]. In the Northwest Pacific—covering China, Taiwan, and Hong Kong—croaker capture rose from 1,178–1,758 thousand tonnes during the 1950s–1970s with the use of bottom trawlers. However, it dropped to 948 thousand tonnes in the 1980s, the lowest point, before surging dramatically to 9,170 thousand tonnes in the 2010s. China consistently dominates the harvest, contributing 69–99% of the catch by weight, primarily to supply domestic markets. In contrast, Taiwan and Hong Kong account for less than 24% and 9% of the catch, respectively. Mudskipper *Boleophalmus* sp. is increasingly threatened by illegal hunting and habitat loss due to coastal development, particularly impacting mudflat ecosystems. The Mai Po Marshes, the most important mudflat habitat in the region, have reported instances of illegal hunting despite being a restricted area (entry and fishing are prohibited without a permit to protect its rich biodiversity in restricted areas) [48]. The abundance of these prey species could severely impact the survival rate of the vulnerable ray species, further driving them toward extinction. Thus, urgent conservation measures such as establishing sustainable fishing practices through regulating minimum catch on Sciaenidae fish, strengthening the enforcement against illegal fishing, and preserving habitat connectivity and diversity in coastal zones are needed to protect the vulnerable ray species.

### Ecological stability and resilience through dietary diversity

The bipartite network plots reveal a diverse diet among the demersal fish species, highlighting their unique foraging preferences and adaptive feeding strategies (Fig 6). The web of interactions underscores the generalist feeding behaviors of these fish, which consume a wide variety of prey species rather than specializing in a single prey type. This dietary diversity contributes significantly to high ecological stability within the food web, allowing for a more balanced energy transfer between the bottom seafloor and the water column.

Such generalist behaviors are particularly advantageous in maintaining ecological equilibrium, as they mitigate the potential impacts on fish energy intake caused by fluctuations in prey availability. For instance, overfishing or environmental changes, such as habitat loss and fragmentation due to coastal developments, can drastically alter the abundance of particular prey species. However, the ability of these fish to shift to alternative food sources enhances their resilience, ensuring their survival amidst these pressures [49,50]. This adaptability is crucial, especially in regions like Hong Kong, where intensive fishing efforts and large-scale coastal infrastructure projects have significantly impacted benthic habitats.

By broadening their diet, these fish species not only secure their own survival but also support the overall sustainability of local fisheries. Such resilience is essential for coping with the challenges posed by anthropogenic activities, and it emphasizes the importance of understanding trophic interactions within the food web. The insights gained from these analyses are invaluable for developing effective conservation strategies aimed at preserving both the fish species and the rich biodiversity of their marine environments.

### Limitations of current studies

Our study has several limitations that constrain the inference of prey selection and trophic dynamics among demersal fish in Hong Kong’s southern and southwestern waters. One major limitation is the restricted temporal and seasonal coverage. Sampling was confined to daylight hours, potentially biasing observations of community composition and feeding behavior by excluding nocturnal activity and diel feeding shifts. Additionally, the two-month sampling period limits our findings to the early wet season. Previous studies, such as Lo et al. [51], have demonstrated significant seasonal dietary differences in marine fish communities in Hong Kong’s western and eastern waters. As a result, our short sampling duration may fail to capture diet variations associated with seasonal fluctuations in prey abundance and availability, limiting the scope and comprehensiveness of the food web models.

The stable isotope technique, while valuable for understanding trophic level differences and overall food web structure, also has inherent limitations. Fish, being highly mobile organisms, often move among habitats with differing isotopic baselines, which can introduce spatial and temporal variability in isotopic signatures. Moreover, stable isotope analysis lacks the resolution to identify specific prey species, providing only broad-level insights into predator-prey relationships.

Molecular methods, though powerful, face challenges that may affect their accuracy. Contamination risks and the use of a single primer pair can introduce primer bias, potentially leading to false positives, false negatives, or misidentification of prey species. Additionally, digestive enzymes in predators’ gut contents can degrade prey DNA, hindering its extraction and amplification. This degradation reduces the reliability of prey detection during molecular analysis, as highlighted by Boyer et al. [52].

### Future perspectives

To address these limitations and advance our understanding of trophic ecology, future studies should expand to include a broader range of fish species and geographic areas in Hong Kong’s waters. Long-term monitoring of fish populations and their diets is essential to evaluate the impacts of coastal developments and climate change. Sampling efforts should be conducted across both hydrological seasons (wet and dry) and during both daytime and nighttime to capture seasonal and diel variations in fish diets. Collecting dietary data that span all life stages is also critical for understanding trophic dynamics throughout a species’ life cycle.

Standardization of life stages across species would further improve the reliability of trophic studies. Maturity should be measured and included as a covariate in statistical models, or dietary data should be stratified by life stage for more accurate analysis. Increasing stage-specific sample sizes, recording gonad maturity for calculating the Gonadosomatic Index (GSI), and applying stage-structured niche and network models would provide deeper insights into trophic dynamics and ecosystem resilience.

The integration of advanced techniques such as metabarcoding and remote sensing offers great potential for enhancing our understanding of prey selection and habitat use. Remote sensing, for example, allows detailed mapping of benthic habitats, such as seagrass beds and muddy substrates, using high-resolution imagery, bathymetric data, and multibeam backscatter analysis. Satellite-derived parameters, including chlorophyll-a concentrations and sediment distribution, help identify productivity hotspots and physical gradients influencing prey availability. When combined with dietary data from metabarcoding, these spatial datasets provide a holistic understanding of prey selection and help predict how environmental changes may alter trophic interactions. This integrated approach offers a powerful framework for linking habitat conditions to food web dynamics.

Collaboration among researchers, policymakers, and local communities is vital for developing sustainable fishing and conservation strategies. Reliable fisheries and ecological data provided by researchers can support evidence-based management measures, such as spatial protections for key habitats. Additionally, funding for knowledge-exchange platforms, such as open-data portals, is necessary to make scientific information accessible to the general public and increase awareness of marine and fisheries conservation.

By improving our understanding of trophic interactions and ecological resilience, we can better determine the ecological roles of species and the pathways of energy flow within food webs. This knowledge will enable species-specific management strategies that safeguard marine biodiversity in Hong Kong and beyond.

## Conclusions

This study underscores the importance of dietary diversification in maintaining ecological stability and resilience among demersal fish species in Hong Kong’s marine ecosystems. By analyzing the gut contents and stable isotope values, we have demonstrated how these fish species adaptively manage their trophic niches and feeding strategies to coexist in a highly urbanized environment. The generalist feeding behaviors observed contribute to a balanced energy transfer within the food web, allowing these species to mitigate the impacts of environmental fluctuations and anthropogenic pressures, such as intensive fishing and habitat loss. These findings highlight the critical role of trophic dynamics in supporting biodiversity and the sustainability of global fisheries, offering valuable insights for conservation efforts aimed at preserving both fish populations and the rich ecological diversity of their habitats.

## Supporting information

S1 Table Sampling location, water depths, and collection method of fish specimens and the potential prey items. (PDF)

S1 Fig (a, b) The 16 dissected fish species, (c) small fish and marine invertebrate samples used for stable isotope analysis. Scale bars: a, c = 1 cm; b = 5 cm.
(PDF)

S2 Table Stable isotope values and elemental composition for fish specimens. (PDF)

S3 Table List of fish and invertebrate species (potential prey items) from the trawling survey. (PDF)

S4 Table Stable isotope values and elemental composition for small fish and invertebrate species (potential prey items) from the trawling survey. (PDF)

S5 Table Similarity percentage (SIMPER) analysis of food items contributing to diet dissimilarity. (PDF)
