# Supplementary material for: Unveiling the trophic dynamics and ecological roles of demersal fish in Hong Kong: A metabarcoding and isotope analysis approach
Source: PLoS One. 2025 Nov 13;20(11):e0335343. doi: 10.1371/journal.pone.0335343 (PMC12614624; doi:10.1371/journal.pone.0335343)
Supplement: S1 Table — (PDF) [file pone.0335343.s001.pdf]

S1 Table. Sampling location, water depths, and collection method of fish specimens and the potential prey items.

Site	Replicate	Start point		End point		Depth	Temperature	Salinity	Gear	Number of nets
		N. Latitude	E. Longitude	N. Latitude	E. Longitude	(m)[a]	(°C)[b]	(psu)[b]		
1	1	22° 16′ 18″	114° 04′ 44″	22° 16′ 00″	114° 04′ 25″	6 - 9	23	31	Trawling	8
	2	22° 15′ 58″	114° 04′ 50″	22° 17′ 19″	114° 04′ 38″					8
2	1	22° 14′ 09″	114° 05′ 58″	22° 13′ 53″	114° 05′ 24″	9 - 32	23	33	Trawling	8
	2	22° 14′ 05″	114° 04′ 36″	22° 14′ 07″	114° 05′ 32″					5
3	1	22° 11′ 16″	113° 55′ 20″	22° 11′ 29″	113° 54′ 48″	9 - 11	23	31	Trawling	6
	2	22° 11′ 57″	113° 54′ 37″	22° 11′ 30″	113° 55′ 11″					6
4	1	22° 10′ 17″	113° 56′ 29″	22° 10′ 09″	113° 55′ 53″	11 - 16	22	33	Trawling	8
	2	22° 10′ 27″	113° 55′ 49″	22° 10′ 22″	113° 56′ 42″					7
5	1	22° 13′ 37″	114° 02′ 20″	22° 13′ 52″	114° 02′ 59″	7 - 9	26	29	Trawling	8
	2	22° 14′ 09″	114° 03′ 09″	22° 13′ 45″	114° 02′ 51″					8
6	1	22° 11′ 41″	114° 00′ 13″	22° 11′ 32″	114° 00′ 56″	12 - 14	23	32	Trawling	6
	2	22° 11′ 44″	114° 00′ 57″	22° 11′ 55″	114° 00′ 33″					4

7	1	22° 11′ 53″ 114° 10′ 04″ 22° 11′ 21″ 114° 10′ 08″	20 - 39	23	33	Trawling	8
	2	22° 11′ 21″ 114° 10′ 05″ 22° 11′ 32″ 114° 10′ 05″					7
8	1	22° 10′ 01″ 114° 10′ 17″ 22° 10′ 03″ 114° 10′ 46″	20 - 23	23	34	Trawling	7
	2	22° 10′ 06″ 114° 10′ 55″ 22° 09′ 55″ 114° 10′ 29″					6

[a]Water depths (m) for Hong Kong sites are based on data from the Hong Kong Hydrographic Office, Marine Department (https://www.hydro.gov.hk/eng/ENCWeb/www/aboutenc.php)

[b]Temperature (°C) and salinity (psu) for Hong Kong sites are based on data from the Hong Kong Environmental Protection Department (https://www.epd.gov.hk/)
