# Supplementary material for: Unveiling the trophic dynamics and ecological roles of demersal fish in Hong Kong: A metabarcoding and isotope analysis approach
Source: PLoS One. 2025 Nov 13;20(11):e0335343. doi: 10.1371/journal.pone.0335343 (PMC12614624; doi:10.1371/journal.pone.0335343)
Supplement: S1 Fig — Scale bars: a, c = 1 cm; b = 5 cm. (PDF) [file pone.0335343.s002.pdf]

S1 Fig. (a, b) The 16 dissected fish species, (c) small fish and marine invertebrate samples used for stable isotope analysis. Scale bars: a, c = 1 cm; b = 5 cm.

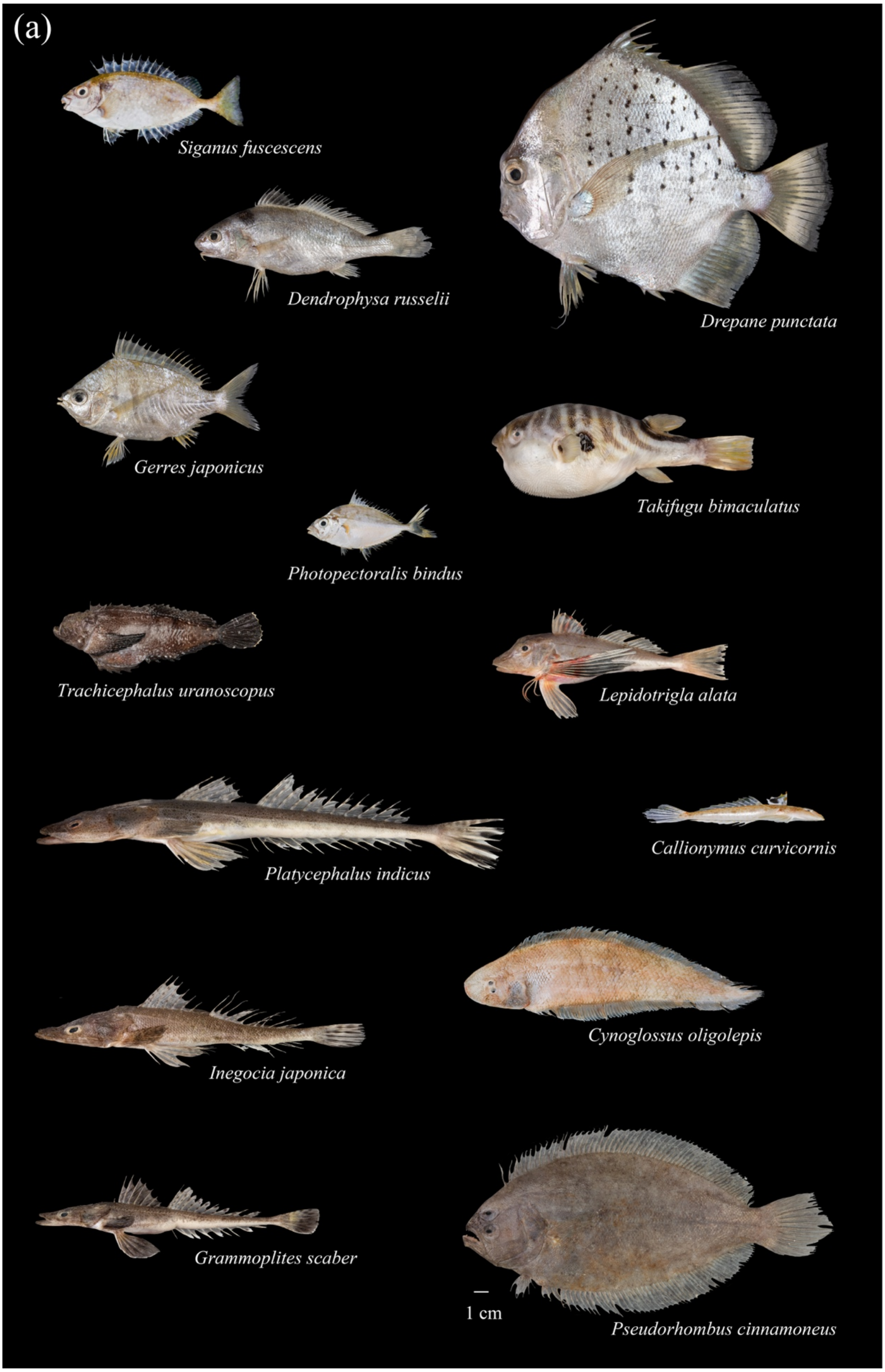

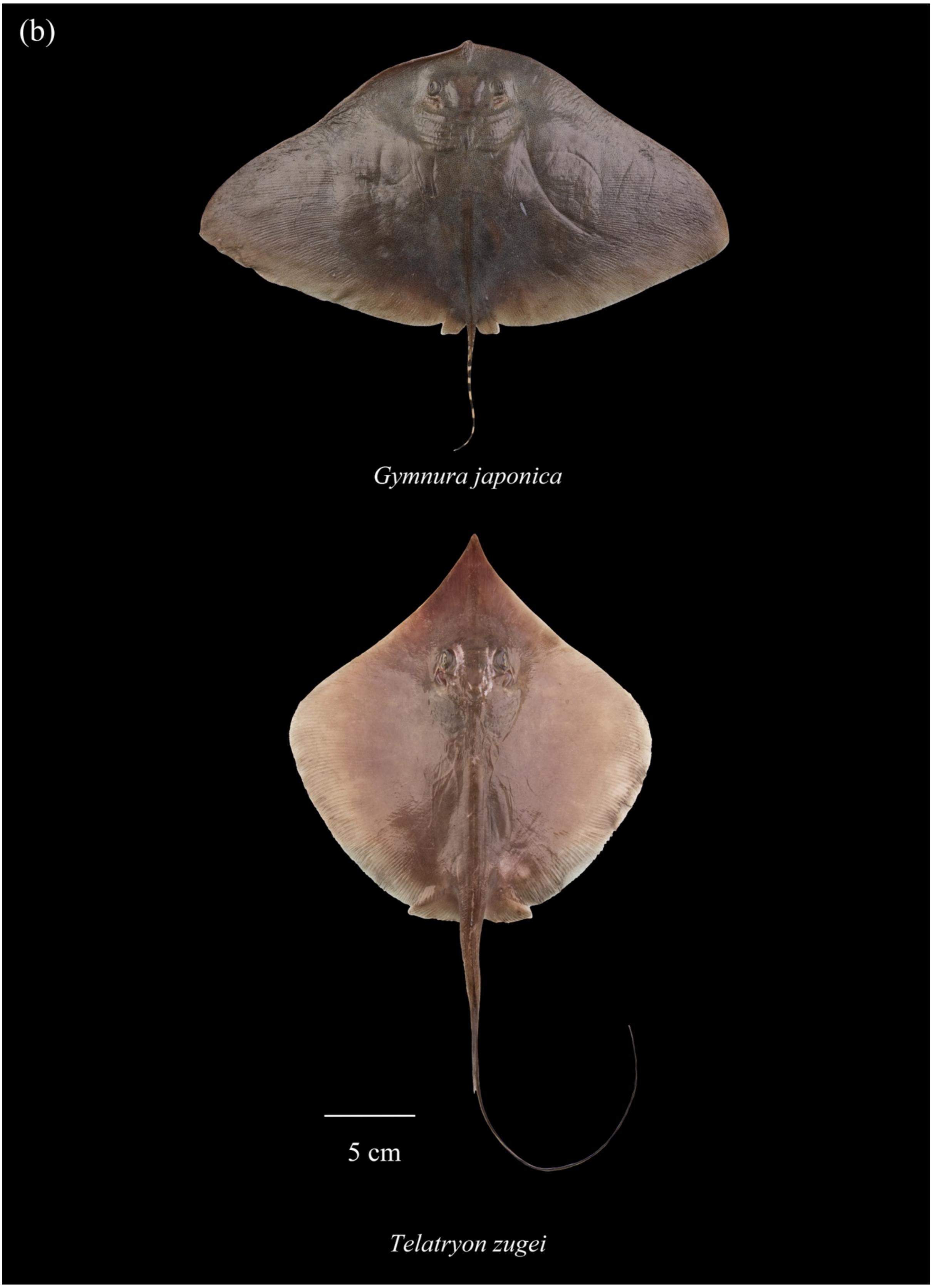
(b)
Gymnura japonica
5 cm
Telatryon zugei

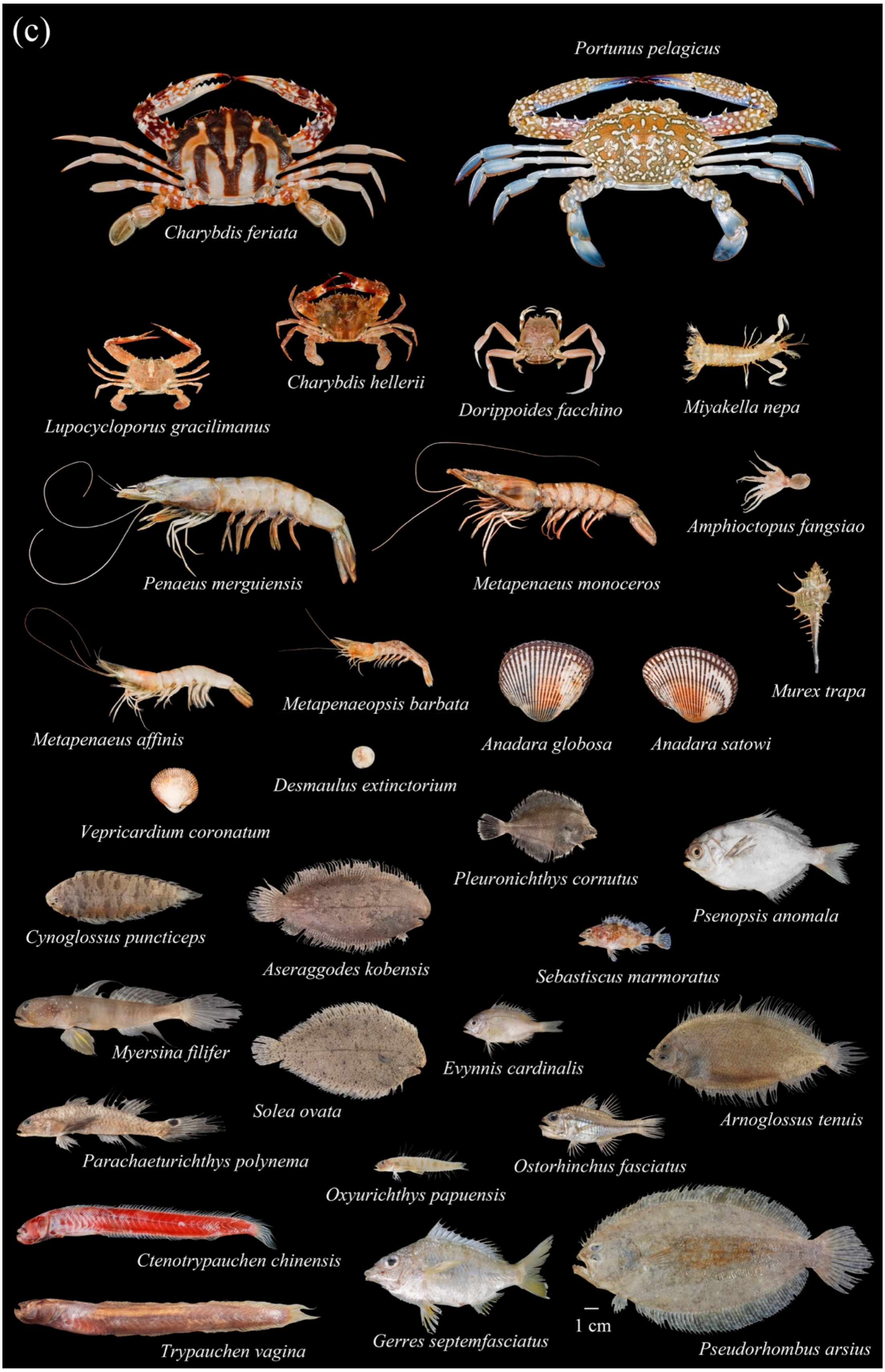
(c)
Portunus pelagicus
Charybdis feriata
Charybdis hellerii
Lupocycloporus gracilimanus
Dorippoides facchino
Miyakella nepa
Amphioctopus fangsiao
Penaeus merguiensis
Metapenaeus monoceros
Murex trapa
Metapenaeopsis barbata
Metapenaeus affinis
Anadara globosa
Anadara satowi
Desmaulus extinctorium
Vepricardium coronatum
Pleuronichthys cornutus
Psenopsis anomala
Cynoglossus puncticeps
Aseraggodes kobensis
Sebastiscus marmoratus
Myersina filifer
Solea ovata
Evynnis cardinalis
Arnoglossus tenuis
Parachaeturichthys polynema
Ostorhinchus fasciatus
Oxyurichthys papuensis
Ctenotrypauchen chinensis
Gerres septemfasciatus
1 cm
Trypauchen vagina
Pseudorhombus arsius
