# Supplementary material for: Unveiling the trophic dynamics and ecological roles of demersal fish in Hong Kong: A metabarcoding and isotope analysis approach
Source: PLoS One. 2025 Nov 13;20(11):e0335343. doi: 10.1371/journal.pone.0335343 (PMC12614624; doi:10.1371/journal.pone.0335343)
Supplement: S2 Table — (PDF) [file pone.0335343.s003.pdf]

S2 Table. Morphological parameters, stable isotope values, and elemental composition for fish specimens.

Order	Species	TL (cm)	SL or DW (cm)	Weight (g)	Life stage	$\delta^{13}C_{untreated}$	$\delta^{15}N$	Carbon (%)	Nitrogen (%)	C:N ratio	$\delta^{13}C_{normalized}$
Acanthuriformes	*Drepane punctata*	26	21	399	Adult	-17.53	12.22	48.85	11.77	4.15	-16.74
		23	18	344	Adult	-17.09	13.41	43.70	13.34	3.28	-
		21	17	276	Adult	-17.31	12.92	43.88	13.30	3.30	-
		23	19	355	Adult	-16.24	13.17	46.10	13.68	3.37	-
		23	19	368	Adult	-16.43	12.32	45.88	13.40	3.42	-
		21	18	263	Adult	-16.24	12.99	45.76	13.62	3.36	-
		19	16	194	Adult	-17.29	13.36	44.43	13.46	3.30	-
		19	16	274	Adult	-16.53	12.95	45.80	13.53	3.38	-
		21	17	268	Adult	-16.00	12.66	46.78	12.91	3.62	-15.74
	Photopectoralis bindus	11	9	18	Adult	-17.71	11.01	49.86	11.25	4.43	-16.64
		10	8	14	Adult	-18.03	10.02	46.00	12.76	3.60	-17.78
		10	8	13	Adult	-17.65	10.50	43.87	12.69	3.46	-
		9	7	10	Adult	-19.36	13.75	43.60	13.01	3.35	-
		8	7	5	Adult	-19.72	13.20	42.67	13.20	3.23	-
		11	9	19	Adult	-17.72	11.70	46.27	12.05	3.84	-17.28
		11	9	22	Adult	-18.29	13.15	48.90	11.18	4.37	-17.28
		7	6	4	Adult	-19.89	12.45	41.71	13.21	3.16	-
		7	6	5	Adult	-18.97	13.56	42.36	12.70	3.33	-
		7	6	4	Adult	-18.95	12.69	42.44	13.78	3.08	-

		9	7	9	Adult	-15.94	12.22	45.49	14.51	3.13	-
		10	8	13	Adult	-15.68	12.29	45.17	14.74	3.07	-
	Siganus fuscescens	15	12	52	Adult	-16.67	11.86	43.39	13.11	3.31	-
		15	13	51	Adult	-16.50	12.10	43.40	12.57	3.45	-
		12	12	30	Adult	-15.27	12.33	48.01	14.99	3.20	-
		13	11	42	Adult	-15.82	13.14	45.95	14.43	3.18	-
		15	12	56	Adult	-15.66	12.87	46.25	14.50	3.19	-
Callionymiformes	*Callionymus curvicornis*	13	10	13	Adult	-17.03	8.35	44.71	14.10	3.17	-
		14	11	14	Adult	-17.14	8.13	55.06	17.42	3.16	-
		13	10	12	Adult	-17.07	8.06	44.92	13.96	3.22	-
		14	11	13	Adult	-17.18	9.53	43.07	13.79	3.12	-
		16	12	17	Adult	-16.72	11.63	43.39	13.52	3.21	-
		11	9	13	Adult	-17.34	7.98	43.06	13.28	3.24	-
		10	8	11	Adult	-17.20	7.50	42.88	13.62	3.15	-
		7	6	2	Adult	-17.00	9.58	41.11	12.92	3.18	-
		10	8	6	Adult	-17.01	9.51	42.83	13.74	3.12	-
		12	9	10	Adult	-16.95	9.34	43.15	13.76	3.14	-
		9	7	4	Adult	-16.94	8.89	42.37	13.37	3.17	-
		9	7	4	Adult	-16.76	9.00	43.07	13.66	3.15	-
		10	8	6	Adult	-16.97	8.36	44.25	13.95	3.17	-
		11	8	7	Adult	-16.96	8.21	44.00	13.90	3.17	-
		9	7	4	Adult	-16.97	8.40	43.46	13.48	3.22	-
		9	7	4	Adult	-16.87	8.62	43.24	13.39	3.23	-
	Dendrophysa russelii	17	14	53	Adult	-15.86	11.74	45.52	13.28	3.43	-

		16	13	41	Adult	-15.53	11.39	43.60	13.43	3.25	-
		16	13	44	Adult	-16.93	12.51	47.19	13.22	3.57	-16.71
		17	14	63	Adult	-17.02	13.01	47.16	13.11	3.60	-16.78
		15	13	39	Adult	-16.61	11.90	45.99	13.09	3.51	-16.45
		13	11	49	Adult	-18.40	8.60	44.03	13.12	3.36	-
		15	13	43	Adult	-16.88	10.84	47.24	12.84	3.68	-16.56
		15	12	40	Adult	-16.38	11.29	46.09	13.39	3.44	-
		15	13	42	Adult	-16.10	12.06	46.55	13.43	3.47	-
		15	12	40	Adult	-16.59	11.75	46.46	12.97	3.58	-16.37
		11	9	13	Adult	-16.96	11.27	43.87	13.39	3.28	-
		13	10	18	Adult	-22.49	16.17	44.84	13.29	3.37	-
Eupercaria *incertae sedis*		15	12	37	Adult	-16.77	12.55	47.34	12.79	3.70	-16.43
		15	13	37	Adult	-16.19	11.23	44.39	13.41	3.31	-
		15	12	38	Adult	-18.82	14.29	43.55	13.24	3.29	-
		15	12	40	Adult	-17.38	13.27	43.11	13.21	3.26	-
		13	11	24	Adult	-19.08	13.30	45.63	13.54	3.37	-
		15	12	34	Adult	-17.17	12.10	48.19	12.74	3.78	-16.74
		15	12	34	Adult	-17.02	11.46	45.39	13.66	3.32	-
		13	10	22	Adult	-19.59	14.22	45.43	13.68	3.32	-
		15	12	36	Adult	-16.53	11.67	47.67	13.71	3.48	-
		12	10	37	Adult	-20.64	14.54	51.52	11.21	4.59	-19.41
		14	11	50	Adult	-18.81	11.53	48.76	11.12	4.39	-17.79
	Gerres japonicus	12	10	30	Adult	-18.39	9.06	46.46	12.98	3.58	-18.16
		12	10	32	Adult	-18.54	11.11	48.86	12.54	3.90	-18.00
		13	10	39	Adult	-18.26	12.68	50.71	11.66	4.35	-17.28

		12	9	30	Adult	-20.22	16.58	46.20	13.30	3.47	-
Myliobatiformes	*Gymnura japonica*	35	22	557	Adult	-15.70	13.20	41.76	15.83	2.64	-
		29	20	393	Adult	-15.70	15.00	40.09	15.30	2.62	-
		21	14	132	Adult	-14.57	15.82	43.37	16.12	2.69	-
		49	31	1703	Adult	-15.76	13.78	46.03	15.36	3.00	-
		42	27	1056	Adult	-15.98	12.93	45.72	15.81	2.89	-
	Telatrygon zugei	52	26	99	Adult	-15.29	11.75	40.46	14.93	2.71	-
		55	23	266	Adult	-15.94	13.27	44.09	16.31	2.70	-
		63	25	319	Adult	-15.96	13.25	44.13	16.53	2.67	-
		62	24	355	Adult	-15.94	13.48	44.53	16.49	2.70	-
		52	23	244	Adult	-16.17	13.36	43.31	15.96	2.71	-
		31	15	68	Adult	-15.85	11.48	41.42	15.22	2.72	-
		38	14	65	Adult	-16.36	10.99	43.90	15.92	2.76	-
		50	23	252	Adult	-15.80	10.60	42.82	15.79	2.71	-
		54	23	253	Adult	-16.30	10.90	42.35	15.87	2.67	-
		35	16	75	Adult	-15.29	11.50	43.00	15.61	2.75	-
		44	17	119	Adult	-16.19	13.40	43.09	16.18	2.66	-
		45	19	147	Adult	-16.04	13.21	42.48	16.34	2.60	-
Perciformes	*Grammoplites scaber*	18	16	28	Adult	-17.69	14.50	47.89	13.91	3.44	-
		21	19	57	Adult	-18.51	14.29	44.18	14.10	3.13	-
		19	17	40	Adult	-18.92	13.75	44.84	14.18	3.16	-
		18	16	25	Adult	-16.95	13.20	45.62	13.88	3.29	-
		15	12	39	Juvenile	-16.55	6.21	41.38	10.42	3.97	-15.94
		39	35	478	Adult	-17.42	14.50	46.38	14.27	3.25	-

	22	19	65	Adult	-16.22	11.18	45.40	14.19	3.20	-
	22	19	69	Adult	-16.34	12.44	46.05	14.68	3.14	-
	23	20	85	Adult	-16.24	12.69	46.64	14.77	3.16	-
	26	23	114	Adult	-16.02	11.76	45.84	14.51	3.16	-
	25	15	104	Adult	-15.90	12.81	46.08	14.77	3.12	-
	9	8	6	Juvenile	-16.90	11.63	45.22	13.69	3.30	-
	16	14	26	Adult	-18.22	13.01	45.85	13.93	3.29	-
	17	15	31	Adult	-16.40	12.62	46.19	14.23	3.25	-
	16	14	31	Adult	-16.30	10.78	45.74	14.02	3.26	-
	17	14	30	Adult	-17.83	13.34	45.47	13.98	3.25	-
	15	13	21	Adult	-16.29	12.38	45.60	13.98	3.26	-
	17	15	35	Adult	-19.79	13.45	44.54	13.85	3.22	-
Inegocia japonica	15	13	22	Adult	-17.84	13.09	45.92	14.17	3.24	-
	21	19	68	Adult	-17.01	10.87	46.01	14.17	3.25	-
	19	17	42	Adult	-16.42	12.21	45.74	14.30	3.20	-
	21	18	67	Adult	-16.13	12.52	46.18	14.19	3.25	-
	20	17	49	Adult	-16.47	10.77	45.95	14.25	3.23	-
	20	18	54	Adult	-15.62	12.14	45.26	14.19	3.19	-
	21	18	66	Adult	-16.00	11.62	45.61	14.15	3.22	-
	20	17	53	Adult	-17.86	13.93	45.42	14.18	3.20	-
	19	16	44	Adult	-15.63	11.22	43.99	13.69	3.21	-
	20	18	59	Adult	-16.49	10.76	45.59	14.28	3.19	-
	17	14	31	Adult	-17.89	13.06	46.57	14.29	3.26	-
	17	14	32	Adult	-17.12	12.66	46.27	14.22	3.25	-
	19	16	44	Adult	-15.45	11.57	42.25	13.13	3.22	-

	18	15	36	Adult	-17.03	12.73	45.60	14.00	3.26	-
	19	16	45	Adult	-18.37	15.35	46.24	14.11	3.28	-
	17	15	36	Adult	-16.30	12.71	46.04	14.10	3.27	-
	19	17	49	Adult	-16.15	12.65	46.62	14.66	3.18	-
	20	18	55	Adult	-18.99	16.35	45.74	14.48	3.16	-
Lepidotrigla alata	16	13	42	Adult	-16.09	12.38	44.61	13.85	3.22	-
	12	10	23	Adult	-16.10	12.44	44.46	13.71	3.24	-
	12	10	20	Adult	-16.21	12.21	43.05	13.54	3.18	-
	8	6	6	Juvenile	-16.47	11.87	42.10	13.26	3.18	-
	9	7	8	Juvenile	-15.91	11.94	42.20	13.23	3.19	-
Platycephalus indicus	34	30	271	Adult	-16.98	12.10	46.17	14.06	3.28	-
	37	33	305	Adult	-20.41	14.60	45.49	14.03	3.24	-
	31	29	184	Adult	-15.67	11.81	46.65	14.48	3.22	-
	34	31	253	Adult	-17.40	14.15	45.53	14.32	3.18	-
	32	29	194	Adult	-18.23	13.59	46.25	14.11	3.28	-
	21	18	45	Adult	-17.41	13.25	43.74	13.76	3.18	-
	29	25	133	Adult	-15.68	12.25	46.14	14.30	3.23	-
	29	26	140	Adult	-15.75	12.09	45.68	14.23	3.21	-
	28	25	154	Adult	-16.25	9.84	44.59	13.97	3.19	-
	23	21	68	Adult	-17.80	10.78	44.60	13.71	3.25	-
Trachicephalus uranoscopus	10	9	25	Adult	-16.31	10.80	43.33	13.55	3.20	-
	12	9	32	Adult	-16.31	10.76	42.93	13.49	3.18	-
	10	8	16	Adult	-17.60	14.49	44.74	13.89	3.22	-
	11	9	21	Adult	-16.07	13.85	44.27	13.90	3.19	-

	11	9	29	Adult	-15.91	12.85	44.13	13.75	3.21	-
	10	8	15	Adult	-15.39	12.06	43.94	13.63	3.22	-
	9	7	12	Adult	-15.93	12.29	42.90	13.23	3.24	-
	9	7	13	Adult	-16.19	11.68	44.18	13.63	3.24	-
	8	7	10	Adult	-16.43	11.03	43.34	13.26	3.27	-
	8	7	9	Adult	-15.97	13.75	43.97	13.50	3.26	-
	9	8	13	Adult	-16.31	12.27	44.72	13.79	3.24	-
	9	7	11	Adult	-16.20	13.45	43.80	13.60	3.22	-
	9	7	11	Adult	-16.88	13.66	43.38	13.50	3.21	-
	9	7	13	Adult	-16.56	13.46	43.60	13.55	3.22	-
	9	7	12	Adult	-16.58	13.56	44.08	13.56	3.25	-
	10	8	14	Adult	-16.42	13.12	44.54	13.69	3.25	-
	10	8	19	Adult	-16.50	12.90	44.26	13.46	3.29	-
	11	9	21	Adult	-16.60	13.30	45.29	13.52	3.35	-
	24	23	79	Adult	-15.72	10.56	43.17	13.63	3.17	-
	30	27	139	Adult	-16.04	12.33	45.38	14.32	3.17	-
	39	37	341	Adult	-15.70	12.79	43.53	13.75	3.17	-
	17	16	24	Juvenile	-17.29	9.46	43.29	13.65	3.17	-
	22	20	59	Adult	-16.36	9.40	45.64	13.97	3.27	-
Pleuronectiformes *Cynoglossus oligolepis*	22	21	61	Adult	-16.18	9.93	44.37	13.56	3.27	-
	17	15	21	Juvenile	-16.79	9.55	44.81	13.79	3.25	-
	12	11	7	Juvenile	-16.40	11.40	44.45	13.48	3.30	-
	12	11	8	Juvenile	-16.50	10.80	44.76	13.67	3.27	-
	21	20	51	Adult	-16.20	11.50	56.01	16.89	3.32	-
	15	13	15	Juvenile	-17.46	12.44	43.76	13.83	3.16	-

	19	18	36	Adult	-16.01	12.24	43.47	13.79	3.15	-
	23	21	61	Adult	-16.10	12.38	43.64	13.97	3.12	-
	24	22	74	Adult	-15.83	10.43	44.69	14.01	3.19	-
	23	22	56	Adult	-17.82	12.63	44.81	13.97	3.21	-
	22	21	59	Adult	-16.03	9.76	43.54	13.69	3.18	-
	20	19	40	Adult	-16.90	11.20	46.11	13.96	3.30	-
	20	19	41	Adult	-16.20	10.10	45.99	13.87	3.32	-
	22	21	56	Adult	-16.90	9.60	45.80	13.87	3.30	-
	21	20	50	Adult	-16.31	11.23	35.34	10.80	3.27	-
Pseudorhombus cinnamoneus	23	19	116	Adult	-21.23	16.66	46.25	14.18	3.26	-
	22	18	97	Adult	-17.72	13.86	44.81	13.66	3.28	-
	23	20	123	Adult	-23.54	17.13	46.03	14.08	3.27	-
	24	20	120	Adult	-16.51	14.03	45.72	13.99	3.27	-
	25	22	135	Adult	-17.86	12.15	44.19	13.75	3.21	-
	10	9	15	Juvenile	-17.18	9.22	43.87	13.07	3.36	-
	12	11	18	Juvenile	-17.21	8.71	42.53	12.86	3.31	-
	10	9	13	Juvenile	-16.70	8.89	44.28	13.52	3.27	-
	12	10	18	Juvenile	-17.17	11.64	45.38	13.82	3.28	-
	21	19	108	Adult	-18.68	14.08	45.33	13.89	3.26	-
	11	9	15	Juvenile	-18.35	11.69	45.31	13.57	3.34	-
	11	9	14	Juvenile	-17.06	11.24	45.31	13.65	3.32	-
	11	9	15	Juvenile	-16.81	10.98	44.83	13.66	3.28	-
	11	10	17	Juvenile	-17.06	11.29	45.46	13.76	3.30	-
	11	9	14	Juvenile	-17.32	11.13	45.70	13.86	3.30	-
	19	20	128	Adult	-16.45	14.06	46.14	14.04	3.29	-

	13	11	19	Juvenile	-16.28	9.78	44.33	13.85	3.20	-
	11	9	13	Juvenile	-16.32	9.69	44.26	13.87	3.19	-
	11	9	13	Juvenile	-15.98	9.97	44.80	13.88	3.23	-
	10	9	11	Juvenile	-16.09	9.98	44.48	14.04	3.17	-
	10	9	12	Juvenile	-15.79	10.37	44.25	13.81	3.21	-
	6	7	4	Juvenile	-16.69	11.74	40.66	12.68	3.21	-
	12	10	16	Juvenile	-16.59	11.74	40.35	12.49	3.23	-
	9	7	7	Juvenile	-17.02	11.67	41.83	13.01	3.21	-
	20	16	62	Adult	-18.42	13.72	35.46	11.01	3.22	-
	10	8	9	Juvenile	-16.76	11.71	41.84	12.81	3.27	-
	10	8	8	Juvenile	-16.71	11.72	42.43	13.00	3.26	-
	9	7	6	Juvenile	-16.64	11.33	39.74	12.19	3.26	-
	8	7	4	Juvenile	-17.40	9.69	44.26	13.24	3.34	-
	8	7	5	Juvenile	-17.20	11.30	44.06	13.16	3.35	-
	28	23	459	Adult	-17.35	12.41	44.66	13.82	3.23	-
	29	25	561	Adult	-17.95	12.40	44.97	13.72	3.28	-
	26	22	342	Adult	-17.33	12.69	44.64	13.94	3.20	-
Tetraodontiformes *Takifugu bimaculatus*	29	25	562	Adult	-17.14	12.60	44.84	13.90	3.23	-
	28	22	474	Adult	-17.32	12.00	45.74	14.06	3.25	-
	14	11	76	Juvenile	-17.86	11.87	44.14	13.76	3.21	-
	25	21	370	Adult	-19.24	13.51	45.18	13.92	3.25	-
