# Supplementary material for: Unveiling the trophic dynamics and ecological roles of demersal fish in Hong Kong: A metabarcoding and isotope analysis approach
Source: PLoS One. 2025 Nov 13;20(11):e0335343. doi: 10.1371/journal.pone.0335343 (PMC12614624; doi:10.1371/journal.pone.0335343)
Supplement: S3 Table — (PDF) [file pone.0335343.s004.pdf]

S3 Table. List of fish and invertebrate species (potential prey items) from the trawling survey.

Major groups	Groups	Family	Species	Number of sample	Number used for isotope analysis
Cnidarian	Coral	Gorgoniidae	*Gorgonia* sp.	15	0
	Sea anemone	-	Unknown sp.	26	0
Crustacean	Barnacle	Balanidae	*Striatobalanus amaryllis*	6	0
		-	Unknown sp.	1	0
	Decapoda	Alpheidae	*Alpheus digitalis*	59	0
		Alpheidae	*Alpheus parvirostris*	1	0
		Alpheidae	*Alpheus splendidus*	1	0
		Alpheidae	*Alpheus* sp.	5	0
		Calappidae	*Calappa bilineata*	1	0
		Diogenidae	*Clibanarius infraspinatus*	3	0
		Diogenidae	*Clibanarius longitarsus*	2	0
		Diogenidae	*Dardanus* sp.	13	0
		Diogenidae	*Diogenes dubius*	18	0
		Diogenidae	*Diogenes rectimanus*	30	0
		Diogenidae	*Diogenes* sp.	25	0
		Dorippidae	*Dorippe quadridens*	12	0
		Dorippidae	*Dorippoides facchino*	214	1
		Dorippidae	*Heikeopsis arachnoides*	5	0
		Dorippidae	*Heikeopsis japonica*	2	0
		Dorippidae	*Paradorippe granulata*	29	0
		Dromiidae	*Lauridromia dehaani*	1	0

Epialtidae	*Doclea ovis*	1	0
Epialtidae	*Doclea* sp.	1	0
Euryplacidae	*Eucrate alcocki*	19	0
Euryplacidae	*Eucrate crenata*	117	0
Euryplacidae	*Eucrate* sp.	1	0
Galenidae	*Galene bispinosa*	2	0
Hippolytidae	*Tozeuma* sp.	3	0
Leucosiidae	*Arcania heptacantha*	25	0
Leucosiidae	*Hiplyra platycheir*	127	0
Leucosiidae	*Lyphira heterograna*	119	0
Leucosiidae	*Myra affinis*	3	0
Leucosiidae	*Myra celeris*	2	0
Leucosiidae	*Nursia plicata*	1	0
Leucosiidae	*Seulocia latirostrata*	24	0
Leucosiidae	*Seulocia* sp.	8	0
Leucosiidae	*Seulocia vittata*	7	0
Lysmatidae	*Lysmata vittata*	2	0
Macrophthalmidae	*Venitus latreillii*	46	0
Majidae	*Leptomithrax* sp.	1	0
Parthenopidae	*Enoplolambrus validus*	40	0
Parthenopidae	*Enoplolambrus* sp.	16	0
Penaeidae	*Alcockpenaeopsis hungerfordii*	55	0
Penaeidae	*Batepenaeopsis tenella*	28	0
Penaeidae	*Metapenaeopsis barbata*	142	1
Penaeidae	*Metapenaeopsis palmensis*	19	0

Penaeidae	*Metapenaeus affinis*	79	1
Penaeidae	*Metapenaeus ensis*	2	0
Penaeidae	*Metapenaeus intermedius*	4	0
Penaeidae	*Metapenaeus joyneri*	42	0
Penaeidae	*Metapenaeus monoceros*	21	2
Penaeidae	*Mierspenaeopsis hardwickii*	56	0
Penaeidae	*Trachysalambria curvirostris*	12	0
Penaeidae	*Trachysalambria longipes*	3	0
Penaeus	*Penaeus canaliculatus*	2	0
Penaeus	*Penaeus merguiensis*	27	2
Penaeus	*Penaeus penicillatus*	1	0
Penaeus	*Penaeus semisulcatus*	4	0
Porcellanidae	*Petrolisthes* sp.	1	0
Porcellanidae	*Pisidia serratifrons*	1	0
Porcellanidae	*Porcellanella triloba*	35	0
Porcellanidae	*Raphidopus ciliatus*	16	0
Portunidae	*Charybdis (Charybdis) affinis*	20	0
Portunidae	*Charybdis (Gonioneptunus) bimaculata*	29	0
Portunidae	*Charybdis (Charybdis) feriata*	13	1
Portunidae	*Charybdis (Charybdis) hellerii*	18	3
Portunidae	*Charybdis (Archias) hongkongensis*	70	0
Portunidae	*Charybdis (Charybdis) japonica*	4	0
Portunidae	*Charybdis (Charybdis) lucifer*	3	0
Portunidae	*Charybdis (Archias) truncata*	22	0
Portunidae	*Charybdis (Charybdis) variegata*	832	0

	Portunidae	*Charybdis* sp.	7	0
	Portunidae	*Eodemus hastatoides*	169	0
	Portunidae	*Eodemus pseudohastatoides*	90	0
	Portunidae	*Eodemus unidens*	4	0
	Portunidae	*Lupocycloporus gracilimanus*	20	2
	Portunidae	*Portunus pelagicus*	43	2
	Portunidae	*Portunus sanguinolentus*	216	0
	Portunidae	*Portunus trituberculatus*	1	0
	Portunidae	*Portunus* sp.	2	0
	Portunidae	*Thalamita admete*	2	0
	Portunidae	*Xiphonectes* sp.	1	0
	Rhynchocinetidae	*Rhynchocinetes brucei*	4	0
	Scalopidiidae	*Scalopidia spinosipes*	39	0
	Scalopidiidae	*Scalopidia* sp.	14	0
	Sergestidae	*Acetes japonicus*	2	0
	Solenoceridae	*Solenocera crassicornis*	4	0
	Xanthidae	*Demania scaberrima*	2	0
	-	Unknown sp.	83	0
	Squillidae	*Anchisquilla* sp.	2	0
	Squillidae	*Carinosquilla multicarinata*	2	0
	Squillidae	*Clorida bombayensis*	4	0
Stomatopoda	Squillidae	*Clorida decorata*	2	0
	Squillidae	*Harpiosquilla harpax*	25	0
	Squillidae	*Miyakella nepa*	144	1
	Squillidae	*Oratosquillina interrupta*	18	0

		Squillidae	*Oratosquilla oratoria*	93	0
Echinoderm	Brittle star	Amphilimnidae	Unknown sp.	1	0
		Ophiotrichidae	Unknown sp.	7	0
		Ophiuridae	Unknown sp.	2	0
		-	Unknown sp.	40	0
	Feather star	-	Unknown sp.	1	0
	Sea cucumber	-	Unknown sp.	82	0
	Sea urchin	Temnopleuridae	*Temnopleurus toreumaticus*	5	0
		-	Unknown sp.	1	0
	Starfish	-	Unknown sp.	1	0
Fish		Apogonidae	*Jaydia truncata*	1	0
		Apogonidae	*Ostorhinchus fasciatus*	21	6
		Apogonidae	*Ostorhinchus novemfasciatus*	1	0
		Apogonidae	*Ostorhinchus* sp.	1	0
		Ariidae	*Arius maculatus*	1	0
		Bothidae	*Arnoglossus tenuis*	80	1
		Bothidae	*Arnoglossus* sp.	3	0
		Bothidae	*Engyprosopon grandisquama*	4	0
		Callionymidae	*Callionymus curvicornis*	17	0
		Callionymidae	*Callionymus hindsii*	6	0
		Callionymidae	*Callionymus* sp.	92	0
		Carangidae	*Trachinotus blochii*	1	0
		Carangidae	*Trachurus japonicus*	9	0
		Centrolophidae	*Psenopsis anomala*	4	2

Cynoglossidae	*Cynoglossus joyneri*	2	0
Cynoglossidae	*Cynoglossus kopsii*	1	0
Cynoglossidae	*Cynoglossus lingua*	5	0
Cynoglossidae	*Cynoglossus macrolepidotus*	16	0
Cynoglossidae	*Cynoglossus oligolepis*	32	0
Cynoglossidae	*Cynoglossus puncticeps*	8	6
Cynoglossidae	*Cynoglossus roulei*	1	0
Cynoglossidae	*Cynoglossus semilaevis*	1	0
Cynoglossidae	*Cynoglossus* sp.	38	0
Cynoglossidae	*Paraplagusia blochii*	1	0
Dactylopteridae	*Dactyloptena orientalis*	1	0
Dasyatidae	*Hemitrygon bennettii*	7	0
Dasyatidae	*Telatrygon zugei*	23	0
Dorosomatidae	*Nematalosa japonica*	4	0
Dorosomatidae	*Sardinella albella*	2	0
Drepaneidae	*Drepane punctata*	13	0
Epinephelidae	*Epinephelus awoara*	1	0
Epinephelidae	*Epinephelus fasciatomaculosus*	1	0
Gerreidae	*Gerres erythrourus*	55	0
Gerreidae	*Gerres filamentosus*	4	0
Gerreidae	*Gerres japonicus*	8	0
Gerreidae	*Gerres limbatus*	2	0
Gerreidae	*Gerres oyena*	1	0
Gerreidae	*Gerres septemfasciatus*	37	1
Gerreidae	*Gerres* sp.	48	0

Gobiidae	*Acentrogobius caninus*	3	0
Gobiidae	*Amblyotrypauchen arctocephalus*	2	0
Gobiidae	*Ctenotrypauchen chinensis*	5	1
Gobiidae	*Myersina filifer*	15	5
Gobiidae	*Oxyurichthys papuensis*	8	1
Gobiidae	*Oxyurichthys* sp.	30	0
Gobiidae	*Parachaeturichthys polynema*	11	1
Gobiidae	*Paratrypauchen microcephalus*	5	0
Gobiidae	*Trypauchen vagina*	15	2
Gobiidae	Unknown sp.	19	0
Gymnuridae	*Gymnura japonica*	15	0
Hapalogenyidae	*Hapalogenys analis*	1	0
Hapalogenyidae	*Hapalogenys nigripinnis*	1	0
Leiognathidae	*Nuchequula nuchalis*	69	0
Leiognathidae	*Photopectoralis bindus*	54	0
Monacanthidae	*Monacanthus chinensis*	4	0
Mugilidae	*Osteomugil cunnesius*	2	0
Muraenesocidae	*Muraenesox cinereus*	1	0
Nemipteridae	*Nemipterus japonicus*	1	0
Ophichthidae	*Pisodonophis cancrivorus*	1	0
Paralichthyidae	*Paralichthys olivaceus*	1	0
Paralichthyidae	*Pseudorhombus arsius*	5	1
Paralichthyidae	*Pseudorhombus cinnamoneus*	156	0
Paralichthyidae	*Pseudorhombus oligodon*	1	0
Paralichthyidae	*Pseudorhombus* sp.	13	2

Pinguipedidae	*Parapercis ommatura*	3	0
Platycephalidae	*Grammoplites scaber*	34	0
Platycephalidae	*Inegocia japonica*	137	0
Platycephalidae	*Platycephalus cultellatus*	3	0
Platycephalidae	*Platycephalus indicus*	11	0
Pleuronectidae	*Pleuronichthys cornutus*	4	0
Sciaenidae	*Dendrophysa russelii*	39	0
Sciaenidae	*Johnius belangerii*	2	0
Sciaenidae	*Johnius borneensis*	4	0
Sciaenidae	*Johnius carouna*	4	0
Sciaenidae	*Johnius taiwanensis*	11	0
Sciaenidae	*Johnius trewavasae*	1	0
Sciaenidae	*Johnius* sp.	49	0
Sciaenidae	*Nibea albiflora*	2	0
Sciaenidae	*Otolithes ruber*	3	0
Sciaenidae	*Pennahia aneus*	1	0
Sciaenidae	*Pennahia argentata*	2	0
Sciaenidae	*Pennahia pawak*	4	0
Sebastidae	*Sebastiscus marmoratus*	8	2
Siganidae	*Siganus fuscescens*	13	0
Sillaginidae	*Sillago asiatica*	2	0
Sillaginidae	*Sillago japonica*	1	0
Soleidae	*Aseraggodes kobensis*	42	1
Soleidae	*Solea ovata*	1072	7
Soleidae	*Zebrias quagga*	1	0

		Soleidae	*Zebrias zebra*	1	0
		Soleidae	*Zebrias zebrinus*	1	0
		Sparidae	*Acanthopagrus pacificus*	1	0
		Sparidae	*Evynnis cardinalis*	145	3
		Synanceiidae	*Minous pusillus*	1	0
		Synanceiidae	*Trachicephalus uranoscopus*	96	0
		Syngnathidae	*Trachyrhamphus serratus*	2	0
		Synodontidae	*Saurida elongata*	10	0
		Synodontidae	*Saurida undosquamis*	7	0
		Terapontidae	*Pelates quadrilineatus*	1	0
		Terapontidae	*Terapon theraps*	1	0
		Tetraodontidae	*Lagocephalus wheeleri*	3	0
		Tetraodontidae	*Takifugu bimaculatus*	10	0
		Tetraodontidae	*Takifugu poecilonotus*	3	0
		Triglidae	*Chelidonichthys spinosus*	4	0
		Triglidae	*Lepidotrigla abyssalis*	1	0
		Triglidae	*Lepidotrigla alata*	10	0
		Triglidae	*Lepidotrigla kishinouyi*	1	0
		Triglidae	*Lepidotrigla* sp.	11	0
		Uranoscopidae	*Xenocephalus elongatus*	1	0
		Arcidae	*Anadara consociata*	1	0
		Arcidae	*Anadara globosa*	6	4
Mollusk	Bivalvia	Arcidae	*Anadara satowi*	3	2
		Arcidae	*Anadara* sp.	21	2
		Cardiidae	*Vepricardium coronatum*	38	1

	Cetoconchidae	*Cetoconcha hyalina*	2	0
	Corbulidae	*Corbula smithiana*	1	0
	Laternulidae	*Exolaternula liautaudi*	2	0
	Ostreidae	*Planostrea pestigris*	1	0
	Tellinidae	*Psammacoma candida*	1	0
	Veneridae	*Placamen lamellatum*	35	0
	Veneridae	*Placamen* sp.	10	0
	-	Unknown sp.	2	0
Cephalopoda	Octopodidae	*Amphioctopus fangsiao*	11	1
	Sepiolidae	*Euprymna berryi*	5	0
	Loliginidae	*Loliolus sumatrensis*	1	0
	Sepiidae	*Acanthosepion aculeatum*	24	0
Gastropoda	Architectonicidae	*Architectonica maculata*	2	0
	Arminidae	*Armina* sp.	1	0
	Bursidae	*Bufonaria* sp.	7	2
	Bursidae	*Marsupina nana*	1	0
	Calyptraeidae	*Desmaulus extinctorium*	31	1
	Chromodorididae	*Chromodoris lineolata*	1	0
	Clavatulidae	*Turricula javana*	7	0
	Clavatulidae	*Turricula nelliae*	133	0
	Epitoniidae	*Cycloscala hyalina*	1	0
	Mitridae	*Domiporta circula*	2	0
	Muricidae	*Murex trapa*	31	1
	Nassariidae	*Nassarius conoidalis*	1	0

	Nassariidae	*Nassarius siquijorensis*	62	0
	Nassariidae	*Tritia reticulata*	1	0
	Naticidae	*Mammilla mammata*	9	0
	Philinidae	*Philine paucipapillata*	2	0
	Philinidae	*Philine* sp.	67	0
	Pseudomelatomidae	*Cheungbeia mindanensis*	2	0
	Pseudomelatomidae	*Funa* sp.	22	0
	Pseudomelatomidae	*Ptychobela suturalis*	8	0
	Terebridae	*Duplicaria duplicata*	9	0
	Turritellidae	*Turritella bacillum*	92	0
	Veretillidae	*Cavernularia obesa*	1	0
Polychaeta	Amphinomidae	*Chloeia* sp.	3	0
	-	Unknown sp.	8	0
Sea pen	Cardiidae	*Vepricardium coronatum*	1	0
	Pennatulidae	*Pteroeides sparmannii*	77	0
	Veretillidae	*Cavernularia obesa*	510	0
	Veretillidae	*Lituaria* sp.	9	0
	Veretillidae	*Veretillum* sp.	2	0
Tunicate	Styelidae	*Polycarpa* sp.	1	0
	-	Unknown sp.	1	0
