# Supplementary material for: Unveiling the trophic dynamics and ecological roles of demersal fish in Hong Kong: A metabarcoding and isotope analysis approach
Source: PLoS One. 2025 Nov 13;20(11):e0335343. doi: 10.1371/journal.pone.0335343 (PMC12614624; doi:10.1371/journal.pone.0335343)
Supplement: S4 Table — (PDF) [file pone.0335343.s005.pdf]

S4 Table. Stable isotope values and elemental composition for small fish and invertebrate species (potential prey items) from the trawling survey.

Major groups	Groups	Family	Species	$\delta^{13}C_{untreated}$	$\delta^{15}N$	Carbon (%)	Nitrogen (%)	C:N ratio	$\delta^{13}C_{normalized}$
Crustacean	Decapoda	Dorippidae	*Dorippoides facchino*	-17.00	7.00	36.09	10.72	3.37	-
		Penaeidae	*Metapenaeopsis barbata*	-16.06	9.88	39.61	12.61	3.14	-
		Penaeidae	*Metapenaeus affinis*	-24.14	14.01	41.59	12.43	3.35	-
		Penaeidae	*Metapenaeus monoceros*	-19.75	13.46	44.94	14.76	3.04	-
		Penaeidae	*Metapenaeus monoceros*	-19.39	9.34	44.50	14.77	3.01	-
		Penaeus	*Penaeus merguiensis*	-16.20	10.70	43.19	13.92	3.10	-
		Penaeus	*Penaeus merguiensis*	-16.42	12.23	44.53	14.85	3.00	-
		Portunidae	*Charybdis (Charybdis) feriata*	-17.17	9.19	42.58	13.03	3.27	-
		Portunidae	*Charybdis (Charybdis) hellerii*	-16.56	10.58	37.84	11.04	3.43	-
		Portunidae	*Charybdis (Charybdis) hellerii*	-15.79	11.39	38.23	11.11	3.44	-
		Portunidae	*Charybdis (Charybdis) hellerii*	-15.90	9.57	35.86	9.81	3.65	-15.60
		Portunidae	*Lupocycloporus gracilimanus*	-15.22	12.86	45.63	14.28	3.19	-
		Portunidae	*Lupocycloporus gracilimanus*	-16.35	12.51	37.55	10.76	3.49	-
		Portunidae	*Portunus pelagicus*	-16.10	7.10	39.86	12.67	3.15	-
		Portunidae	*Portunus pelagicus*	-14.98	9.75	43.15	13.16	3.28	-
	Stomatopoda	Squillidae	*Miyakella nepa*	-16.80	6.80	40.36	11.87	3.40	-
Fish		Apogonidae	*Ostorhinchus fasciatus*	-16.29	13.57	43.18	13.55	3.19	-
		Apogonidae	*Ostorhinchus fasciatus*	-16.89	13.29	41.75	13.09	3.19	-
		Apogonidae	*Ostorhinchus fasciatus*	-16.96	13.48	42.37	13.34	3.18	-
		Apogonidae	*Ostorhinchus fasciatus*	-17.08	12.65	43.32	13.22	3.28	-

Apogonidae	*Ostorhinchus fasciatus*	-16.80	12.80	42.09	13.11	3.21	-
Apogonidae	*Ostorhinchus fasciatus*	-16.40	13.72	46.01	14.26	3.23	-
Bothidae	*Arnoglossus tenuis*	-16.93	11.56	43.35	13.17	3.29	-
Centrolophidae	*Psenopsis anomala*	-16.72	13.58	46.84	14.86	3.15	-
Centrolophidae	*Psenopsis anomala*	-17.16	13.32	47.05	14.79	3.18	-
Cynoglossidae	*Cynoglossus puncticeps*	-17.85	13.20	42.86	13.03	3.29	-
Cynoglossidae	*Cynoglossus puncticeps*	-16.58	12.13	44.30	12.94	3.42	-
Cynoglossidae	*Cynoglossus puncticeps*	-24.84	18.88	42.92	12.90	3.33	-
Cynoglossidae	*Cynoglossus puncticeps*	-24.43	17.97	42.79	12.69	3.37	-
Cynoglossidae	*Cynoglossus puncticeps*	-18.10	14.60	45.29	13.63	3.32	-
Cynoglossidae	*Cynoglossus puncticeps*	-16.50	13.30	43.96	13.32	3.30	-
Gerreidae	*Gerres septemfasciatus*	-17.04	11.92	47.34	11.99	3.95	-16.45
Gobiidae	*Ctenotrypauchen chinensis*	-17.59	10.77	36.35	11.07	3.28	-
Gobiidae	*Myersina filifer*	-15.94	11.70	43.21	13.23	3.27	-
Gobiidae	*Myersina filifer*	-16.26	11.74	42.47	13.22	3.21	-
Gobiidae	*Myersina filifer*	-16.73	12.21	43.12	13.11	3.29	-
Gobiidae	*Myersina filifer*	-16.90	11.70	41.67	13.30	3.13	-
Gobiidae	*Myersina filifer*	-16.70	12.20	41.55	13.07	3.18	-
Gobiidae	*Oxyurichthys papuensis*	-15.75	11.18	45.36	14.69	3.09	-
Gobiidae	*Parachaeturichthys polynema*	-16.76	10.30	42.99	13.80	3.11	-
Gobiidae	*Trypauchen vagina*	-16.31	12.29	45.24	13.24	3.42	-
Gobiidae	*Trypauchen vagina*	-16.97	12.82	43.93	12.68	3.46	-
Paralichthyidae	*Pseudorhombus arsius*	-16.76	13.36	42.37	12.84	3.30	-
Paralichthyidae	*Pseudorhombus* sp.	-17.00	12.10	44.60	13.75	3.24	-
Paralichthyidae	*Pseudorhombus* sp.	-16.30	13.10	45.97	14.01	3.28	-

		Sebastidae	*Sebastiscus marmoratus*	-16.42	12.28	41.37	12.73	3.25	-
		Sebastidae	*Sebastiscus marmoratus*	-16.40	12.51	41.60	12.78	3.25	-
		Soleidae	*Aseraggodes kobensis*	-16.00	12.54	42.38	12.97	3.27	-
		Soleidae	*Solea ovata*	-19.30	5.98	40.96	12.61	3.25	-
		Soleidae	*Solea ovata*	-16.10	12.50	38.64	12.76	3.03	-
		Soleidae	*Solea ovata*	-15.40	10.90	40.49	12.63	3.21	-
		Soleidae	*Solea ovata*	-16.77	12.26	41.66	12.83	3.25	-
		Soleidae	*Solea ovata*	-16.83	12.71	42.04	13.09	3.21	-
		Soleidae	*Solea ovata*	-16.40	12.66	42.91	13.46	3.19	-
		Soleidae	*Solea ovata*	-16.02	13.04	41.97	13.18	3.18	-
		Sparidae	*Evynnis cardinalis*	-17.34	10.81	44.43	13.19	3.37	-
		Sparidae	*Evynnis cardinalis*	-17.37	10.88	43.16	12.85	3.36	-
		Sparidae	*Evynnis cardinalis*	-17.59	9.75	43.55	13.17	3.31	-
Mollusk	Bivalvia	Arcidae	*Anadara globosa*	-16.13	7.14	39.03	9.98	3.91	-15.58
		Arcidae	*Anadara globosa*	-15.24	9.35	42.99	12.70	3.38	-
		Arcidae	*Anadara globosa*	-14.73	9.15	42.82	13.44	3.19	-
		Arcidae	*Anadara globosa*	-15.01	9.07	42.37	12.46	3.40	-
		Arcidae	*Anadara satowi*	-16.46	8.23	39.76	9.60	4.14	-15.68
		Arcidae	*Anadara satowi*	-16.20	8.20	41.12	11.27	3.65	-15.91
		Arcidae	*Anadara* sp.	-16.52	7.84	39.10	10.16	3.85	-16.03
		Arcidae	*Anadara* sp.	-15.62	10.95	42.45	13.19	3.22	-
		Cardiidae	*Vepricardium coronatum*	-17.03	4.21	36.86	7.21	5.11	-15.29
	Cephalopoda	Octopodidae	*Amphioctopus fangsiao*	-15.59	11.55	43.99	13.89	3.17	-
	Gastropoda	Bursidae	*Bufonaria* sp.	-15.60	11.80	42.48	12.63	3.36	-

Bursidae	*Bufonaria* sp.	-14.49	10.96	41.38	12.36	3.35	-
Calyptraeidae	*Desmaulus extinctorium*	-18.18	3.90	38.37	7.26	5.29	-16.26
Muricidae	*Murex trapa*	-16.00	7.98	41.95	11.84	3.54	-15.81
