# Supplementary material for: Unveiling the trophic dynamics and ecological roles of demersal fish in Hong Kong: A metabarcoding and isotope analysis approach
Source: PLoS One. 2025 Nov 13;20(11):e0335343. doi: 10.1371/journal.pone.0335343 (PMC12614624; doi:10.1371/journal.pone.0335343)
Supplement: S5 Table — (PDF) [file pone.0335343.s006.pdf]

S5 Table. Similarity percentage (SIMPER) analysis of food items contributing to diet dissimilarity.

Food items	*C. curvicornis*	*G. japonica*	**Average dissimilarity = 75.84%**			
	Avg. Abund.	Avg. Abund.	Avg. Diss.	Diss./SD	Contrib. %	Cum. %
Fish	510.20	42950.00	71.27	2.30	94.00	94.00
Brittle star	70.80	0.00	2.79	0.37	3.70	97.70
Decapoda	50.40	0.00	1.71	0.74	2.20	99.90
Gastropoda	1.00	0.00	0.05	0.33	0.10	100.00
Copepoda	0.60	0.00	0.02	0.34	0.00	100.00

Food items	*C. curvicornis*	*T. zugei*	**Average dissimilarity = 86.96%**			
	Avg. Abund.	Avg. Abund.	Avg. Diss.	Diss./SD	Contrib. %	Cum. %
Decapoda	50.40	18110.00	49.57	1.85	57.00	57.00
Fish	510.20	26420.00	35.87	1.30	41.30	98.30
Brittle star	70.80	19.00	1.49	0.58	1.70	100.00
Gastropoda	1.00	0.00	0.02	0.34	0.00	100.00
Copepoda	0.60	0.00	0.01	0.35	0.00	100.00

Food items	*C. curvicornis*	*G. scaber*	**Average dissimilarity = 83.78%**			
	Avg. Abund.	Avg. Abund.	Avg. Diss.	Diss./SD	Contrib. %	Cum. %
Decapoda	50.40	27000.00	57.65	1.96	68.80	68.80
Fish	510.20	10530.00	22.69	1.12	27.10	95.90
Brittle star	70.80	0.00	3.30	0.37	3.90	99.80
Gastropoda	1.00	0.00	0.06	0.33	0.10	99.90
Stomatpoda	0.00	19.00	0.06	0.56	0.10	100.00
Copepoda	0.60	0.00	0.02	0.33	0.00	100.00

Food items	*C. curvicornis*	*I. japonica*	**Average dissimilarity = 86.57%**			
	Avg. Abund.	Avg. Abund.	Avg. Diss.	Diss./SD	Contrib. %	Cum. %
Decapoda	50.40	9388.00	66.45	1.87	76.70	76.70
Fish	510.20	171.00	14.15	0.69	16.40	93.10
Brittle star	70.80	0.00	3.25	0.36	3.80	96.90
Stomatpoda	0.00	56.00	2.64	0.76	3.00	99.90
Gastropoda	1.00	0.00	0.06	0.31	0.10	100.00
Copepoda	0.60	0.00	0.02	0.33	0.00	100.00

Food items	*C. curvicornis*	*L. alata*	**Average dissimilarity = 99.31%**			
	Avg. Abund.	Avg. Abund.	Avg. Diss.	Diss./SD	Contrib. %	Cum. %
Decapoda	50.40	173900.00	98.50	61.24	99.20	99.20
Fish	510.20	144.00	0.69	0.63	0.70	99.90
Brittle star	70.80	0.00	0.12	0.44	0.10	100.00
Gastropoda	1.00	0.00	0.00	0.38	0.00	100.00

Copepoda	0.60	0.00	0.00	0.38	0.00	100.00

Food items	*C. curvicornis*	*P. indicus*	**Average dissimilarity = 92.77%**			
	Avg. Abund.	Avg. Abund.	Avg. Diss.	Diss./SD	Contrib. %	Cum. %
Fish	510.20	11070.00	54.97	1.29	59.30	59.30
Stomatpoda	0.00	9863.00	19.98	0.50	21.50	80.80
Decapoda	50.40	763.00	17.03	0.54	18.40	99.20
Brittle star	70.80	0.00	0.77	0.39	0.80	100.00
Gastropoda	1.00	0.00	0.01	0.34	0.00	100.00
Copepoda	0.60	0.00	0.01	0.35	0.00	100.00

Food items	*C. curvicornis*	*T. uranoscopus*	**Average dissimilarity = 99.49%**			
	Avg. Abund.	Avg. Abund.	Avg. Diss.	Diss./SD	Contrib. %	Cum. %
Fish	510.20	215400.00	99.43	179.20	99.90	99.90
Brittle star	70.80	0.00	0.04	0.54	0.10	100.00
Decapoda	50.40	1.00	0.02	0.87	0.00	100.00
Gastropoda	1.00	0.00	0.00	0.47	0.00	100.00
Copepoda	0.60	0.00	0.00	0.47	0.00	100.00

Food items	*C. curvicornis*	*C. oligolepis*	**Average dissimilarity = 83.28%**			
	Avg. Abund.	Avg. Abund.	Avg. Diss.	Diss./SD	Contrib. %	Cum. %
Decapoda	50.40	19090.00	66.41	1.67	79.70	79.70
Fish	510.20	227.00	13.69	0.76	16.50	96.20
Brittle star	70.80	1.00	2.34	0.35	2.80	99.00
Stomatpoda	0.00	24.00	0.78	0.66	0.90	99.90
Gastropoda	1.00	0.00	0.04	0.30	0.10	100.00
Copepoda	0.60	0.00	0.02	0.32	0.00	100.00
Bivalvia	0.00	1.00	0.00	0.68	0.00	100.00

Food items	*C. curvicornis*	*P. cinnamoneus*	**Average dissimilarity = 81.48%**			
	Avg. Abund.	Avg. Abund.	Avg. Diss.	Diss./SD	Contrib. %	Cum. %
Fish	510.20	17830.00	42.99	1.46	52.80	52.80
Decapoda	50.40	3740.00	22.58	0.72	27.70	80.50
Cephalopoda	0.00	344.00	9.31	0.90	11.40	91.90
Isopoda	0.00	2760.00	3.65	0.64	4.50	96.40
Brittle star	70.80	3.00	2.88	0.36	3.50	99.90
Gastropoda	1.00	0.00	0.05	0.30	0.10	100.00
Copepoda	0.60	0.00	0.02	0.32	0.00	100.00

Food items	*C. curvicornis*	*D. punctata*	**Average dissimilarity = 83.53%**			
	Avg. Abund.	Avg. Abund.	Avg. Diss.	Diss./SD	Contrib. %	Cum. %
Fish	510.20	29.20	33.93	1.27	40.60	40.60

Brittle star	70.80	335.20	22.84	1.46	27.40	68.00
Bivalvia	0.00	156.60	12.61	0.79	15.10	83.10
Sea pen	0.00	95.20	4.71	0.57	5.60	88.70
Decapoda	50.40	43.00	4.20	0.82	5.00	93.70
Worm	0.00	32.60	2.39	0.69	2.90	96.60
Gastropoda	1.00	10.80	1.03	0.96	1.20	97.80
Polychaeta	0.00	6.80	0.92	0.46	1.10	98.90
Sea anemone	0.00	6.40	0.85	0.43	1.10	100.00
Copepoda	0.60	0.00	0.05	0.46	0.00	100.00

Food items	*C. curvicornis*	*P. bindus*	**Average dissimilarity = 68.08%**			
	Avg. Abund.	Avg. Abund.	Avg. Diss.	Diss./SD	Contrib. %	Cum. %
Decapoda	50.40	867.60	34.54	1.45	50.70	50.70
Fish	510.20	474.20	23.13	1.52	34.00	84.70
Brittle star	70.80	0.80	4.21	0.52	6.20	90.90
Cephalopoda	0.00	29.40	3.28	0.44	4.80	95.70
Ctenopoda	0.00	29.40	1.76	0.47	2.60	98.30
Worm	0.00	10.00	0.59	0.50	0.90	99.20
Isopoda	0.00	2.80	0.31	0.44	0.40	99.60
Gastropoda	1.00	3.00	0.20	0.86	0.30	99.90
Copepoda	0.60	0.00	0.03	0.46	0.10	100.00
Bivalvia	0.00	0.40	0.03	0.47	0.00	100.00

Food items	*C. curvicornis*	*S. fuscescens*	**Average dissimilarity = 69.30%**			
	Avg. Abund.	Avg. Abund.	Avg. Diss.	Diss./SD	Contrib. %	Cum. %
Fish	510.20	93.00	45.41	1.73	65.50	65.50
Brittle star	70.80	13.20	11.21	0.63	16.20	81.70
Decapoda	50.40	52.00	10.80	0.95	15.60	97.30
Gastropoda	1.00	2.20	0.47	0.77	0.70	98.00
Bivalvia	0.00	2.20	0.44	0.39	0.60	98.60
Amphipoda	0.00	1.80	0.43	0.37	0.60	99.20
Coral	0.00	2.20	0.34	0.58	0.50	99.70
Copepoda	0.60	0.80	0.20	0.49	0.30	100.00

Food items	*C. curvicornis*	*D. russelii*	**Average dissimilarity = 66.30%**			
	Avg. Abund.	Avg. Abund.	Avg. Diss.	Diss./SD	Contrib. %	Cum. %
Fish	510.20	228.80	33.67	1.27	50.80	50.80
Bivalvia	0.00	647.80	15.71	0.52	23.70	74.50
Decapoda	50.40	118.40	8.72	0.83	13.10	87.60
Brittle star	70.80	0.40	7.14	0.49	10.80	98.40
Mollusca	0.00	25.00	0.58	0.49	0.90	99.30

Worm	0.00	11.80	0.27	0.49	0.40	99.70
Gastropoda	1.00	0.00	0.16	0.41	0.20	99.90
Copepoda	0.60	0.00	0.05	0.45	0.10	100.00

Food items	*C. curvicornis*	*G. japonicus*	**Average dissimilarity = 82.23%**			
	Avg. Abund.	Avg. Abund.	Avg. Diss.	Diss./SD	Contrib. %	Cum. %
Fish	510.20	379.20	57.70	2.10	70.20	70.20
Brittle star	70.80	0.00	9.52	0.48	11.60	81.80
Decapoda	50.40	1.00	9.39	0.74	11.40	93.20
Bivalvia	0.00	50.60	5.29	0.80	6.40	99.60
Gastropoda	1.00	0.00	0.27	0.41	0.30	99.90
Copepoda	0.60	0.00	0.06	0.46	0.10	100.00

Food items	*C. curvicornis*	*T. bimaculatus*	**Average dissimilarity = 70.60%**			
	Avg. Abund.	Avg. Abund.	Avg. Diss.	Diss./SD	Contrib. %	Cum. %
Decapoda	50.40	598.00	31.50	1.15	44.60	44.60
Fish	510.20	187.00	31.16	1.30	44.20	88.80
Brittle star	70.80	0.00	6.77	0.50	9.50	98.30
Gastropoda	1.00	9.40	1.13	0.75	1.60	99.90
Copepoda	0.60	0.00	0.04	0.46	0.10	100.00

Food items	*G. japonica*	*T. zugei*	**Average dissimilarity = 83.20%**			
	Avg. Abund.	Avg. Abund.	Avg. Diss.	Diss./SD	Contrib. %	Cum. %
Fish	26420.00	42950.00	50.13	1.77	60.30	60.30
Decapoda	18110.00	0.00	32.70	1.28	39.30	99.60
Brittle star	19.00	0.00	0.37	0.37	0.40	100.00

Food items	*G. japonica*	*G. scaber*	**Average dissimilarity = 90.23%**			
	Avg. Abund.	Avg. Abund.	Avg. Diss.	Diss./SD	Contrib. %	Cum. %
Fish	42950.00	10530.00	50.81	1.51	56.30	56.30
Decapoda	0.00	27000.00	39.38	1.19	43.70	100.00
Stomatpoda	0.00	19.00	0.04	0.50	0.00	100.00

Food items	*G. japonica*	*I. japonica*	**Average dissimilarity = 95.01%**			
	Avg. Abund.	Avg. Abund.	Avg. Diss.	Diss./SD	Contrib. %	Cum. %
Fish	42950.00	171.00	51.81	1.40	54.50	54.50
Decapoda	0.00	9388.00	42.16	1.12	44.40	98.90
Stomatpoda	0.00	56.00	1.04	0.62	1.10	100.00

Food items	*G. japonica*	*L. alata*	**Average dissimilarity = 99.53%**			
	Avg. Abund.	Avg. Abund.	Avg. Diss.	Diss./SD	Contrib. %	Cum. %
Decapoda	0.00	173900.00	81.21	2.89	81.60	81.60
Fish	42950.00	144.00	18.32	0.65	18.40	100.00

Food items	*G. japonica*	*P. indicus*	**Average dissimilarity = 80.41%**			
	Avg. Abund.	Avg. Abund.	Avg. Diss.	Diss./SD	Contrib. %	Cum. %
Fish	42950.00	11070.00	55.24	1.57	68.70	68.70
Stomatpoda	0.00	9863.00	15.61	0.46	19.40	88.10
Decapoda	0.00	763.00	9.56	0.44	11.90	100.00

Food items	*G. japonica*	*T. uranoscopus*	**Average dissimilarity =78.40%**			
	Avg. Abund.	Avg. Abund.	Avg. Diss.	Diss./SD	Contrib. %	Cum. %
Fish	42950.00	215400.00	78.40	2.23	100.00	100.00
Decapoda	0.00	1.00	0.00	0.76	0.00	100.00

Food items	*G. japonica*	*C. oligolepis*	**Average dissimilarity = 87.81%**			
	Avg. Abund.	Avg. Abund.	Avg. Diss.	Diss./SD	Contrib. %	Cum. %
Decapoda	0.00	19090.00	45.70	1.13	52.00	52.00
Fish	42950.00	227.00	41.78	1.07	47.60	99.60
Stomatpoda	0.00	24.00	0.33	0.50	0.40	100.00
Bivalvia	0.00	1.00	0.00	0.62	0.00	100.00
Brittle star	0.00	1.00	0.00	0.62	0.00	100.00

Food items	*G. japonica*	*P. cinnamoneus*	**Average dissimilarity = 79.66%**			
	Avg. Abund.	Avg. Abund.	Avg. Diss.	Diss./SD	Contrib. %	Cum. %
Fish	42950.00	17830.00	57.45	1.77	72.10	72.10
Decapoda	0.00	3740.00	15.11	0.58	19.00	91.10
Cephalopoda	0.00	344.00	4.33	0.68	5.40	96.50
Isopoda	0.00	2760.00	2.71	0.53	3.40	99.90
Brittle star	0.00	3.00	0.06	0.44	0.10	100.00

Food items	*G. japonica*	*D. punctata*	**Average dissimilarity = 98.40%**			
	Avg. Abund.	Avg. Abund.	Avg. Diss.	Diss./SD	Contrib. %	Cum. %
Fish	42950.00	29.20	78.09	3.12	79.40	79.40
Brittle star	0.00	335.20	9.16	0.74	9.30	88.70
Bivalvia	0.00	156.60	5.06	0.52	5.10	93.80
Sea pen	0.00	95.20	2.20	0.41	2.30	96.10
Decapoda	0.00	43.00	1.93	0.62	1.90	98.00
Worm	0.00	32.60	0.95	0.52	1.00	99.00
Gastropoda	0.00	10.80	0.39	0.71	0.40	99.40
Polychaeta	0.00	6.80	0.32	0.35	0.30	99.70
Sea anemone	0.00	6.40	0.30	0.33	0.30	100.00

Food items	*G. japonica*	*P. bindus*	**Average dissimilarity = 76.65%**			
	Avg. Abund.	Avg. Abund.	Avg. Diss.	Diss./SD	Contrib. %	Cum. %
Fish	42950.00	474.20	56.27	1.47	73.40	73.40

Decapoda	0.00	867.60	17.84	0.86	23.30	96.70
Cephalopoda	0.00	29.40	1.22	0.33	1.60	98.30
Ctenopoda	0.00	29.40	0.80	0.35	1.00	99.30
Worm	0.00	10.00	0.27	0.37	0.40	99.70
Isopoda	0.00	2.80	0.12	0.33	0.10	99.80
Gastropoda	0.00	3.00	0.09	0.61	0.10	99.90
Brittle star	0.00	0.80	0.03	0.33	0.10	100.00
Bivalvia	0.00	0.40	0.01	0.35	0.00	100.00

Food items	*G. japonica*	*S. fuscescens*	**Average dissimilarity = 90.26%**			
	Avg. Abund.	Avg. Abund.	Avg. Diss.	Diss./SD	Contrib. %	Cum. %
Fish	42950.00	93.00	86.19	4.94	95.50	95.50
Decapoda	0.00	52.00	2.91	0.54	3.20	98.70
Brittle star	0.00	13.20	0.66	0.49	0.80	99.50
Bivalvia	0.00	2.20	0.12	0.32	0.10	99.60
Gastropoda	0.00	2.20	0.12	0.60	0.10	99.70
Coral	0.00	2.20	0.11	0.42	0.10	99.80
Amphipoda	0.00	1.80	0.11	0.32	0.20	100.00
Copepoda	0.00	0.80	0.04	0.32	0.00	100.00

Food items	*G. japonica*	*D. russelii*	**Average dissimilarity = 83.95%**			
	Avg. Abund.	Avg. Abund.	Avg. Diss.	Diss./SD	Contrib. %	Cum. %
Fish	42950.00	228.80	70.72	2.15	84.20	84.20
Bivalvia	0.00	647.80	8.61	0.41	10.30	94.50
Decapoda	0.00	118.40	4.13	0.79	4.90	99.40
Mollusca	0.00	25.00	0.32	0.39	0.40	99.80
Worm	0.00	11.80	0.15	0.39	0.20	100.00
Brittle star	0.00	0.40	0.02	0.33	0.00	100.00

Food items	*G. japonica*	*G. japonicus*	**Average dissimilarity = 83.77%**			
	Avg. Abund.	Avg. Abund.	Avg. Diss.	Diss./SD	Contrib. %	Cum. %
Fish	42950.00	379.20	82.03	2.66	97.90	97.90
Bivalvia	0.00	50.60	1.67	0.55	2.00	99.90
Decapoda	0.00	1.00	0.07	0.46	0.10	100.00

Food items	*G. japonica*	*T. bimaculatus*	**Average dissimilarity = 87.07%**			
	Avg. Abund.	Avg. Abund.	Avg. Diss.	Diss./SD	Contrib. %	Cum. %
Fish	42950.00	379.20	82.03	2.66	97.90	97.90
Bivalvia	0.00	50.60	1.67	0.55	2.00	99.90
Decapoda	0.00	1.00	0.07	0.46	0.10	100.00

Food items	*T. zugei*	*G. scaber*	**Average dissimilarity = 75.75%**			

	Avg. Abund.	Avg. Abund.	Avg. Diss.	Diss./SD	Contrib. %	Cum. %
Decapoda	18110.00	27000.00	45.83	1.58	60.50	60.50
Fish	26420.00	10530.00	29.49	1.20	38.90	99.40
Brittle star	19.00	0.00	0.39	0.34	0.60	100.00
Stomatpoda	0.00	19.00	0.04	0.48	0.00	100.00
Food items	*T. zugei*	*I. japonica*	**Average dissimilarity = 75.47%**			
	Avg. Abund.	Avg. Abund.	Avg. Diss.	Diss./SD	Contrib. %	Cum. %
Decapoda	18110.00	9388.00	44.67	1.61	59.20	59.20
Fish	26420.00	171.00	29.92	1.10	39.60	98.80
Stomatpoda	0.00	56.00	0.49	0.80	0.70	99.50
Brittle star	19.00	0.00	0.39	0.37	0.50	100.00
Food items	*T. zugei*	*L. alata*	**Average dissimilarity = 79.34%**			
	Avg. Abund.	Avg. Abund.	Avg. Diss.	Diss./SD	Contrib. %	Cum. %
Decapoda	18110.00	173900.00	65.34	1.76	82.40	82.40
Fish	26420.00	144.00	13.97	0.71	17.60	100.00
Brittle star	19.00	0.00	0.03	0.38	0.00	100.00
Food items	*T. zugei*	*P. indicus*	**Average dissimilarity = 80.00%**			
	Avg. Abund.	Avg. Abund.	Avg. Diss.	Diss./SD	Contrib. %	Cum. %
Fish	26420.00	11070.00	44.24	1.53	55.30	55.30
Decapoda	18110.00	763.00	21.84	1.40	27.30	82.60
Stomatpoda	0.00	9863.00	13.76	0.45	17.20	99.80
Brittle star	19.00	0.00	0.16	0.37	0.20	100.00
Food items	*T. zugei*	*T. uranoscopus*	**Average dissimilarity = 82.86%**			
	Avg. Abund.	Avg. Abund.	Avg. Diss.	Diss./SD	Contrib. %	Cum. %
Fish	26420.00	215400.00	76.75	2.83	92.60	92.60
Decapoda	18110.00	1.00	6.10	0.93	7.40	100.00
Brittle star	19.00	0.00	0.01	0.47	0.00	100.00
Food items	*T. zugei*	*C. oligolepis*	**Average dissimilarity = 71.58%**			
	Avg. Abund.	Avg. Abund.	Avg. Diss.	Diss./SD	Contrib. %	Cum. %
Decapoda	18110.00	19090.00	44.78	1.37	62.60	62.60
Fish	26420.00	227.00	26.31	1.02	36.70	99.30
Brittle star	19.00	1.00	0.33	0.35	0.50	99.80
Stomatpoda	0.00	24.00	0.16	0.55	0.20	100.00
Bivalvia	0.00	1.00	0.00	0.60	0.00	100.00
Food items	*T. zugei*	*P. cinnamoneus*	**Average dissimilarity = 79.03%**			
	Avg. Abund.	Avg. Abund.	Avg. Diss.	Diss./SD	Contrib. %	Cum. %
Fish	26420.00	17830.00	38.83	1.41	49.10	49.10

Decapoda	18110.00	3740.00	34.96	1.33	44.30	93.40
Cephalopoda	0.00	344.00	2.52	0.60	3.20	96.60
Isopoda	0.00	2760.00	2.36	0.49	3.00	99.60
Brittle star	19.00	3.00	0.36	0.36	0.40	100.00
Food items	*T. zugei*	*D. punctata*	**Average dissimilarity = 96.53%**			
	Avg. Abund.	Avg. Abund.	Avg. Diss.	Diss./SD	Contrib. %	Cum. %
Decapoda	18110.00	43.00	49.08	1.86	50.80	50.80
Fish	26420.00	29.20	38.99	1.45	40.40	91.20
Brittle star	19.00	335.20	4.08	0.62	4.30	95.50
Bivalvia	0.00	156.60	2.33	0.49	2.40	97.90
Sea pen	0.00	95.20	1.20	0.39	1.20	99.10
Worm	0.00	32.60	0.45	0.50	0.50	99.60
Gastropoda	0.00	10.80	0.17	0.80	0.20	99.80
Polychaeta	0.00	6.80	0.12	0.37	0.10	99.90
Sea anemone	0.00	6.40	0.11	0.34	0.10	100.00
Food items	*T. zugei*	*P. bindus*	**Average dissimilarity = 73.68%**			
	Avg. Abund.	Avg. Abund.	Avg. Diss.	Diss./SD	Contrib. %	Cum. %
Decapoda	18110.00	867.60	39.88	1.81	54.10	54.10
Fish	26420.00	474.20	32.07	1.20	43.60	97.70
Brittle star	19.00	0.80	0.58	0.49	0.80	98.50
Cephalopoda	0.00	29.40	0.50	0.34	0.60	99.10
Ctenopoda	0.00	29.40	0.41	0.35	0.60	99.70
Worm	0.00	10.00	0.14	0.38	0.20	99.90
Isopoda	0.00	2.80	0.05	0.34	0.00	99.90
Gastropoda	0.00	3.00	0.04	0.64	0.10	100.00
Bivalvia	0.00	0.40	0.01	0.35	0.00	100.00
Food items	*T. zugei*	*S. fuscescens*	**Average dissimilarity = 94.42%**			
	Avg. Abund.	Avg. Abund.	Avg. Diss.	Diss./SD	Contrib. %	Cum. %
Decapoda	18110.00	52.00	52.93	1.84	56.00	56.00
Fish	26420.00	93.00	40.46	1.43	42.90	98.90
Brittle star	19.00	13.20	0.87	0.56	0.90	99.80
Bivalvia	0.00	2.20	0.04	0.34	0.10	99.90
Gastropoda	0.00	2.20	0.04	0.64	0.00	99.90
Coral	0.00	2.20	0.04	0.42	0.10	100.00
Amphipoda	0.00	1.80	0.03	0.34	0.00	100.00
Copepoda	0.00	0.80	0.01	0.34	0.00	100.00
Food items	*T. zugei*	*D. russelii*	**Average dissimilarity = 90.33%**			
	Avg. Abund.	Avg. Abund.	Avg. Diss.	Diss./SD	Contrib. %	Cum. %

Decapoda	18110.00	118.40	47.28	1.75	52.30	52.30
Fish	26420.00	228.80	36.27	1.31	40.20	92.50
Bivalvia	0.00	647.80	5.75	0.38	6.40	98.90
Brittle star	19.00	0.40	0.71	0.47	0.70	99.60
Mollusca	0.00	25.00	0.22	0.37	0.30	99.90
Worm	0.00	11.80	0.10	0.37	0.10	100.00

Food items	*T. zugei*	*G. japonicus*	**Average dissimilarity = 92.01%**			
	Avg. Abund.	Avg. Abund.	Avg. Diss.	Diss./SD	Contrib. %	Cum. %
Decapoda	18110.00	1.00	52.01	1.86	56.50	56.50
Fish	26420.00	379.20	38.44	1.36	41.80	98.30
Brittle star	19.00	0.00	0.81	0.48	0.90	99.20
Bivalvia	0.00	50.60	0.75	0.50	0.80	100.00

Food items	*T. zugei*	*T. bimaculatus*	**Average dissimilarity = 82.77%**			
	Avg. Abund.	Avg. Abund.	Avg. Diss.	Diss./SD	Contrib. %	Cum. %
Decapoda	18110.00	598.00	45.06	1.72	54.40	54.40
Fish	26420.00	187.00	36.83	1.35	44.50	98.90
Brittle star	19.00	0.00	0.72	0.48	0.90	99.80
Gastropoda	0.00	9.40	0.16	0.62	0.20	100.00

Food items	*G. scaber*	*I. japonica*	**Average dissimilarity = 71.47%**			
	Avg. Abund.	Avg. Abund.	Avg. Diss.	Diss./SD	Contrib. %	Cum. %
Decapoda	27000.00	9388.00	59.95	1.88	83.90	83.90
Fish	10530.00	171.00	10.35	0.74	14.50	98.40
Stomatpoda	19.00	56.00	1.17	0.56	1.60	100.00

Food items	*G. scaber*	*L. alata*	**Average dissimilarity = 72.64%**			
	Avg. Abund.	Avg. Abund.	Avg. Diss.	Diss./SD	Contrib. %	Cum. %
Decapoda	27000.00	173900.00	67.53	1.75	93.00	93.00
Fish	10530.00	144.00	5.09	0.50	7.00	100.00
Stomatpoda	19.00	0.00	0.02	0.46	0.00	100.00

Food items	*G. scaber*	*P. indicus*	**Average dissimilarity = 89.29%**			
	Avg. Abund.	Avg. Abund.	Avg. Diss.	Diss./SD	Contrib. %	Cum. %
Fish	10530.00	11070.00	37.89	0.99	42.40	42.40
Decapoda	27000.00	763.00	36.91	1.16	41.40	83.80
Stomatpoda	19.00	9863.00	14.49	0.45	16.20	100.00

Food items	*G. scaber*	*T. uranoscopus*	**Average dissimilarity = 93.41%**			
	Avg. Abund.	Avg. Abund.	Avg. Diss.	Diss./SD	Contrib. %	Cum. %
Fish	10530.00	215400.00	84.07	4.03	90.00	90.00
Decapoda	27000.00	1.00	9.33	0.90	10.00	100.00

Stomatpoda	19.00	0.00	0.01	0.54	0.00	100.00

Food items	*G. scaber*	*C. oligolepis*	**Average dissimilarity = 72.28%**			
	Avg. Abund.	Avg. Abund.	Avg. Diss.	Diss./SD	Contrib. %	Cum. %
Decapoda	27000.00	19090.00	59.66	1.74	82.50	82.50
Fish	10530.00	227.00	12.22	0.82	16.90	99.40
Stomatpoda	19.00	24.00	0.40	0.50	0.60	100.00
Bivalvia	0.00	1.00	0.00	0.61	0.00	100.00
Brittle star	0.00	1.00	0.00	0.61	0.00	100.00

Food items	*G. scaber*	*P. cinnamoneus*	**Average dissimilarity = 79.98%**			
	Avg. Abund.	Avg. Abund.	Avg. Diss.	Diss./SD	Contrib. %	Cum. %
Decapoda	27000.00	3740.00	42.62	1.29	53.30	53.30
Fish	10530.00	17830.00	30.02	1.09	37.50	90.80
Cephalopoda	0.00	344.00	4.57	0.64	5.70	96.50
Isopoda	0.00	2760.00	2.66	0.54	3.40	99.90
Brittle star	0.00	3.00	0.07	0.41	0.10	100.00
Stomatpoda	19.00	0.00	0.04	0.52	0.00	100.00

Food items	*G. scaber*	*D. punctata*	**Average dissimilarity = 93.48%**			
	Avg. Abund.	Avg. Abund.	Avg. Diss.	Diss./SD	Contrib. %	Cum. %
Decapoda	27000.00	43.00	56.43	1.91	60.40	60.40
Fish	10530.00	29.20	16.30	1.10	17.40	77.80
Brittle star	0.00	335.20	10.24	0.72	11.00	88.80
Bivalvia	0.00	156.60	5.80	0.51	6.20	95.00
Sea pen	0.00	95.20	2.36	0.39	2.50	97.50
Worm	0.00	32.60	1.08	0.50	1.10	98.60
Gastropoda	0.00	10.80	0.46	0.69	0.50	99.10
Polychaeta	0.00	6.80	0.39	0.35	0.50	99.60
Sea anemone	0.00	6.40	0.36	0.33	0.30	99.90
Stomatpoda	19.00	0.00	0.06	0.56	0.10	100.00

Food items	*G. scaber*	*P. bindus*	**Average dissimilarity = 71.86%**			
	Avg. Abund.	Avg. Abund.	Avg. Diss.	Diss./SD	Contrib. %	Cum. %
Decapoda	27000.00	867.60	51.96	1.63	72.30	72.30
Fish	10530.00	474.20	16.90	1.18	23.50	95.80
Cephalopoda	0.00	29.40	1.46	0.33	2.10	97.90
Ctenopoda	0.00	29.40	0.88	0.34	1.20	99.10
Worm	0.00	10.00	0.30	0.35	0.40	99.50
Isopoda	0.00	2.80	0.14	0.33	0.20	99.70
Gastropoda	0.00	3.00	0.11	0.58	0.20	99.90
Stomatpoda	19.00	0.00	0.06	0.56	0.00	99.90

Brittle star	0.00	0.80	0.04	0.33	0.10	100.00
Bivalvia	0.00	0.40	0.01	0.34	0.00	100.00
Food items	*G. scaber*	*S. fuscescens*	**Average dissimilarity = 82.42%**			
	Avg. Abund.	Avg. Abund.	Avg. Diss.	Diss./SD	Contrib. %	Cum. %
Decapoda	27000.00	52.00	63.93	2.61	77.60	77.60
Fish	10530.00	93.00	16.97	1.12	20.60	98.20
Brittle star	0.00	13.20	0.82	0.49	1.00	99.20
Bivalvia	0.00	2.20	0.16	0.33	0.20	99.40
Gastropoda	0.00	2.20	0.15	0.61	0.10	99.50
Amphipoda	0.00	1.80	0.14	0.33	0.20	99.70
Coral	0.00	2.20	0.13	0.42	0.20	99.90
Stomatpoda	19.00	0.00	0.06	0.56	0.00	99.90
Copepoda	0.00	0.80	0.06	0.33	0.10	100.00
Food items	*G. scaber*	*D. russelii*	**Average dissimilarity = 79.80%**			
	Avg. Abund.	Avg. Abund.	Avg. Diss.	Diss./SD	Contrib. %	Cum. %
Decapoda	27000.00	118.40	53.99	1.70	67.60	67.60
Fish	10530.00	228.80	16.83	1.06	21.10	88.70
Bivalvia	0.00	647.80	8.44	0.37	10.60	99.30
Mollusca	0.00	25.00	0.31	0.35	0.40	99.70
Worm	0.00	11.80	0.15	0.35	0.20	99.90
Stomatpoda	19.00	0.00	0.06	0.56	0.10	100.00
Brittle star	0.00	0.40	0.02	0.33	0.00	100.00
Food items	*G. scaber*	*G. japonicus*	**Average dissimilarity = 95.34%**			
	Avg. Abund.	Avg. Abund.	Avg. Diss.	Diss./SD	Contrib. %	Cum. %
Decapoda	27000.00	1.00	65.46	2.46	68.70	68.70
Fish	10530.00	379.20	27.88	1.47	29.20	97.90
Bivalvia	0.00	50.60	1.94	0.55	2.00	99.90
Stomatpoda	19.00	0.00	0.06	0.56	0.10	100.00
Food items	*G. scaber*	*T. bimaculatus*	**Average dissimilarity = 70.41%**			
	Avg. Abund.	Avg. Abund.	Avg. Diss.	Diss./SD	Contrib. %	Cum. %
Decapoda	27000.00	598.00	54.80	1.74	77.80	77.80
Fish	10530.00	187.00	15.04	1.02	21.40	99.20
Gastropoda	0.00	9.40	0.51	0.57	0.70	99.90
Stomatpoda	19.00	0.00	0.06	0.56	0.10	100.00
Food items	*I. japonica*	*L. alata*	**Average dissimilarity = 77.43%**			
	Avg. Abund.	Avg. Abund.	Avg. Diss.	Diss./SD	Contrib. %	Cum. %
Decapoda	9388.00	173900.00	77.21	2.57	99.70	99.70

Fish	171.00	144.00	0.14	1.03	0.20	99.90
Stomatpoda	56.00	0.00	0.08	1.31	0.10	100.00
Food items	*I. japonica*	*P. indicus*	**Average dissimilarity = 90.10%**			
	Avg. Abund.	Avg. Abund.	Avg. Diss.	Diss./SD	Contrib. %	Cum. %
Fish	171.00	11070.00	40.55	1.03	45.00	45.00
Decapoda	9388.00	763.00	32.33	1.13	35.90	80.90
Stomatpoda	56.00	9863.00	17.22	0.49	19.10	100.00
Food items	*I. japonica*	*T. uranoscopus*	**Average dissimilarity = 99.85%**			
	Avg. Abund.	Avg. Abund.	Avg. Diss.	Diss./SD	Contrib. %	Cum. %
Fish	171.00	215400.00	95.62	18.86	95.80	95.80
Decapoda	9388.00	1.00	4.20	0.85	4.20	100.00
Stomatpoda	56.00	0.00	0.03	2.81	0.00	100.00
Food items	*I. japonica*	*C. oligolepis*	**Average dissimilarity = 68.34%**			
	Avg. Abund.	Avg. Abund.	Avg. Diss.	Diss./SD	Contrib. %	Cum. %
Decapoda	9388.00	19090.00	62.81	1.83	91.90	91.90
Fish	171.00	227.00	5.02	0.46	7.30	99.20
Stomatpoda	56.00	24.00	0.51	0.65	0.80	100.00
Bivalvia	0.00	1.00	0.00	0.67	0.00	100.00
Brittle star	0.00	1.00	0.00	0.67	0.00	100.00
Food items	*I. japonica*	*P. cinnamoneus*	**Average dissimilarity = 82.13%**			
	Avg. Abund.	Avg. Abund.	Avg. Diss.	Diss./SD	Contrib. %	Cum. %
Decapoda	9388.00	3740.00	44.27	1.23	53.90	53.90
Fish	171.00	17830.00	28.88	0.94	35.20	89.10
Cephalopoda	0.00	344.00	4.84	0.68	5.90	95.00
Isopoda	0.00	2760.00	3.02	0.56	3.60	98.60
Stomatpoda	56.00	0.00	1.05	0.56	1.30	99.90
Brittle star	0.00	3.00	0.07	0.41	0.10	100.00
Food items	*I. japonica*	*D. punctata*	**Average dissimilarity = 93.61%**			
	Avg. Abund.	Avg. Abund.	Avg. Diss.	Diss./SD	Contrib. %	Cum. %
Decapoda	9388.00	43.00	65.10	1.80	69.50	69.50
Brittle star	0.00	335.20	10.26	0.76	11.00	80.50
Bivalvia	0.00	156.60	5.76	0.52	6.10	86.60
Fish	171.00	29.20	5.33	0.62	5.70	92.30
Stomatpoda	56.00	0.00	2.51	0.77	2.70	95.00
Sea pen	0.00	95.20	2.37	0.41	2.60	97.60
Worm	0.00	32.60	1.08	0.51	1.10	98.70
Gastropoda	0.00	10.80	0.46	0.66	0.50	99.20

Polychaeta	0.00	6.80	0.38	0.34	0.40	99.60
Sea anemone	0.00	6.40	0.36	0.32	0.40	100.00

Food items	*I. japonica*	*P. bindus*	**Average dissimilarity = 74.54%**			
	Avg. Abund.	Avg. Abund.	Avg. Diss.	Diss./SD	Contrib. %	Cum. %
Decapoda	9388.00	867.60	60.42	1.93	81.00	81.00
Fish	171.00	474.20	9.69	0.88	13.10	94.10
Stomatpoda	56.00	0.00	1.53	0.91	2.00	96.10
Cephalopoda	0.00	29.40	1.44	0.32	1.90	98.00
Ctenopoda	0.00	29.40	0.87	0.35	1.20	99.20
Worm	0.00	10.00	0.29	0.36	0.40	99.60
Isopoda	0.00	2.80	0.14	0.32	0.20	99.80
Gastropoda	0.00	3.00	0.11	0.58	0.10	99.90
Brittle star	0.00	0.80	0.04	0.32	0.10	100.00
Bivalvia	0.00	0.40	0.01	0.34	0.00	100.00

Food items	*I. japonica*	*S. fuscescens*	**Average dissimilarity = 84.25%**			
	Avg. Abund.	Avg. Abund.	Avg. Diss.	Diss./SD	Contrib. %	Cum. %
Decapoda	9388.00	52.00	72.18	2.16	85.70	85.70
Fish	171.00	93.00	6.91	0.71	8.20	93.90
Stomatpoda	56.00	0.00	3.70	0.83	4.40	98.30
Brittle star	0.00	13.20	0.82	0.48	0.90	99.20
Bivalvia	0.00	2.20	0.16	0.31	0.20	99.40
Gastropoda	0.00	2.20	0.15	0.56	0.20	99.60
Amphipoda	0.00	1.80	0.14	0.31	0.20	99.80
Coral	0.00	2.20	0.13	0.41	0.10	99.90
Copepoda	0.00	0.80	0.06	0.31	0.10	100.00

Food items	*I. japonica*	*D. russelii*	**Average dissimilarity = 82.34%**			
	Avg. Abund.	Avg. Abund.	Avg. Diss.	Diss./SD	Contrib. %	Cum. %
Decapoda	9388.00	118.40	62.42	1.66	75.80	75.80
Bivalvia	0.00	647.80	9.00	0.42	10.90	86.70
Fish	171.00	228.80	7.81	0.61	9.50	96.20
Stomatpoda	56.00	0.00	2.59	0.74	3.20	99.40
Mollusca	0.00	25.00	0.34	0.40	0.40	99.80
Worm	0.00	11.80	0.16	0.40	0.20	100.00
Brittle star	0.00	0.40	0.02	0.32	0.00	100.00

Food items	*I. japonica*	*G. japonicus*	**Average dissimilarity = 96.75%**			
	Avg. Abund.	Avg. Abund.	Avg. Diss.	Diss./SD	Contrib. %	Cum. %
Decapoda	9388.00	1.00	73.61	2.32	76.10	76.10
Fish	171.00	379.20	17.61	0.80	18.20	94.30

Stomatpoda	56.00	0.00	3.57	0.70	3.70	98.00
Bivalvia	0.00	50.60	1.96	0.56	2.00	100.00

Food items	*I. japonica*	*T. bimaculatus*	**Average dissimilarity = 73.60%**			
	Avg. Abund.	Avg. Abund.	Avg. Diss.	Diss./SD	Contrib. %	Cum. %
Decapoda	9388.00	598.00	64.86	1.94	88.10	88.10
Fish	171.00	187.00	5.79	0.63	7.90	96.00
Stomatpoda	56.00	0.00	2.44	0.79	3.30	99.30
Gastropoda	0.00	9.40	0.51	0.55	0.70	100.00

Food items	*L. alata*	*P. indicus*	**Average dissimilarity = 97.21%**			
	Avg. Abund.	Avg. Abund.	Avg. Diss.	Diss./SD	Contrib. %	Cum. %
Decapoda	763.00	173900.00	77.34	3.85	79.60	79.60
Fish	11070.00	144.00	11.90	0.71	12.20	91.80
Stomatpoda	9863.00	0.00	7.97	0.42	8.20	100.00

Food items	*L. alata*	*T. uranoscopus*	**Average dissimilarity = 99.89%**			
	Avg. Abund.	Avg. Abund.	Avg. Diss.	Diss./SD	Contrib. %	Cum. %
Fish	215400.00	144.00	66.11	2.62	66.20	66.20
Decapoda	1.00	173900.00	33.78	1.33	33.80	100.00

Food items	*L. alata*	*C. oligolepis*	**Average dissimilarity = 72.20%**			
	Avg. Abund.	Avg. Abund.	Avg. Diss.	Diss./SD	Contrib. %	Cum. %
Decapoda	173900.00	19090.00	71.94	2.05	99.70	99.70
Fish	144.00	227.00	0.23	0.69	0.30	100.00
Stomatpoda	0.00	24.00	0.03	0.89	0.00	100.00
Bivalvia	0.00	1.00	0.00	0.56	0.00	100.00
Brittle star	0.00	1.00	0.00	0.56	0.00	100.00

Food items	*L. alata*	*P. cinnamoneus*	**Average dissimilarity = 90.62%**			
	Avg. Abund.	Avg. Abund.	Avg. Diss.	Diss./SD	Contrib. %	Cum. %
Decapoda	173900.00	3740.00	77.30	2.70	85.30	85.30
Fish	144.00	17830.00	11.25	0.56	12.40	97.70
Isopoda	0.00	2760.00	1.55	0.43	1.70	99.40
Cephalopoda	0.00	344.00	0.52	0.50	0.60	100.00
Brittle star	0.00	3.00	0.00	0.58	0.00	100.00

Food items	*L. alata*	*D. punctata*	**Average dissimilarity = 99.75%**			
	Avg. Abund.	Avg. Abund.	Avg. Diss.	Diss./SD	Contrib. %	Cum. %
Decapoda	173900.00	43.00	98.38	57.81	98.60	98.60
Brittle star	0.00	335.20	0.58	0.66	0.60	99.20
Bivalvia	0.00	156.60	0.27	0.51	0.30	99.50
Fish	144.00	29.20	0.26	1.27	0.20	99.70

Sea pen	0.00	95.20	0.16	0.41	0.20	99.90
Worm	0.00	32.60	0.06	0.52	0.10	100.00
Gastropoda	0.00	10.80	0.02	0.94	0.00	100.00
Polychaeta	0.00	6.80	0.01	0.41	0.00	100.00
Sea anemone	0.00	6.40	0.01	0.38	0.00	100.00

Food items	*L. alata*	*P. bindus*	**Average dissimilarity = 96.45%**			
	Avg. Abund.	Avg. Abund.	Avg. Diss.	Diss./SD	Contrib. %	Cum. %
Decapoda	173900.00	867.60	95.81	22.06	99.30	99.30
Fish	144.00	474.20	0.51	0.91	0.60	99.90
Cephalopoda	0.00	29.40	0.05	0.38	0.00	99.90
Ctenopoda	0.00	29.40	0.05	0.38	0.10	100.00
Worm	0.00	10.00	0.02	0.41	0.00	100.00
Gastropoda	0.00	3.00	0.01	0.73	0.00	100.00
Isopoda	0.00	2.80	0.00	0.38	0.00	100.00
Brittle star	0.00	0.80	0.00	0.38	0.00	100.00
Bivalvia	0.00	0.40	0.00	0.38	0.00	100.00

Food items	*L. alata*	*S. fuscescens*	**Average dissimilarity = 99.52%**			
	Avg. Abund.	Avg. Abund.	Avg. Diss.	Diss./SD	Contrib. %	Cum. %
Decapoda	173900.00	52.00	99.30	181.70	99.80	99.80
Fish	144.00	93.00	0.20	1.10	0.20	100.00
Brittle star	0.00	13.20	0.02	0.54	0.00	100.00
Bivalvia	0.00	2.20	0.00	0.38	0.00	100.00
Gastropoda	0.00	2.20	0.00	0.75	0.00	100.00
Coral	0.00	2.20	0.00	0.47	0.00	100.00
Amphipoda	0.00	1.80	0.00	0.38	0.00	100.00
Copepoda	0.00	0.80	0.00	0.38	0.00	100.00

Food items	*L. alata*	*D. russelii*	**Average dissimilarity = 99.18%**			
	Avg. Abund.	Avg. Abund.	Avg. Diss.	Diss./SD	Contrib. %	Cum. %
Decapoda	173900.00	118.40	97.77	30.06	98.60	98.60
Bivalvia	0.00	647.80	1.06	0.39	1.00	99.60
Fish	144.00	228.80	0.29	0.72	0.30	99.90
Mollusca	0.00	25.00	0.04	0.39	0.10	100.00
Worm	0.00	11.80	0.02	0.39	0.00	100.00
Brittle star	0.00	0.40	0.00	0.38	0.00	100.00

Food items	*L. alata*	*G. japonicus*	**Average dissimilarity = 99.76%**			
	Avg. Abund.	Avg. Abund.	Avg. Diss.	Diss./SD	Contrib. %	Cum. %
Decapoda	173900.00	1.00	98.94	73.47	99.20	99.20
Fish	144.00	379.20	0.73	0.88	0.70	99.90

Bivalvia	0.00	50.60	0.09	0.52	0.10	100.00
Food items	*L. alata*	*T. bimaculatus*	**Average dissimilarity = 97.57%**			
	Avg. Abund.	Avg. Abund.	Avg. Diss.	Diss./SD	Contrib. %	Cum. %
Decapoda	173900.00	598.00	97.31	25.69	99.70	99.70
Fish	144.00	187.00	0.24	0.91	0.30	100.00
Gastropoda	0.00	9.40	0.02	0.73	0.00	100.00
Food items	*P. indicus*	*T. uranoscopus*	**Average dissimilarity = 90.36%**			
	Avg. Abund.	Avg. Abund.	Avg. Diss.	Diss./SD	Contrib. %	Cum. %
Fish	11070.00	215400.00	86.09	7.65	95.30	95.30
Stomatpoda	9863.00	0.00	3.90	0.48	4.30	99.60
Decapoda	763.00	1.00	0.37	0.66	0.40	100.00
Food items	*P. indicus*	*C. oligolepis*	**Average dissimilarity = 89.89%**			
	Avg. Abund.	Avg. Abund.	Avg. Diss.	Diss./SD	Contrib. %	Cum. %
Decapoda	763.00	19090.00	37.83	1.14	42.10	42.10
Fish	11070.00	227.00	36.25	0.96	40.30	82.40
Stomatpoda	9863.00	24.00	15.81	0.47	17.60	100.00
Bivalvia	0.00	1.00	0.00	0.66	0.00	100.00
Brittle star	0.00	1.00	0.00	0.66	0.00	100.00
Food items	*P. indicus*	*P. cinnamoneus*	**Average dissimilarity = 82.58%**			
	Avg. Abund.	Avg. Abund.	Avg. Diss.	Diss./SD	Contrib. %	Cum. %
Fish	11070.00	17830.00	45.39	1.26	55.00	55.00
Decapoda	763.00	3740.00	16.95	0.65	20.50	75.50
Stomatpoda	9863.00	0.00	15.66	0.47	19.00	94.50
Isopoda	0.00	2760.00	2.52	0.50	3.00	97.50
Cephalopoda	0.00	344.00	2.04	0.60	2.50	100.00
Brittle star	0.00	3.00	0.02	0.56	0.00	100.00
Food items	*P. indicus*	*D. punctata*	**Average dissimilarity = 98.48%**			
	Avg. Abund.	Avg. Abund.	Avg. Diss.	Diss./SD	Contrib. %	Cum. %
Fish	11070.00	29.20	55.56	1.23	56.40	56.40
Stomatpoda	9863.00	0.00	19.94	0.50	20.30	76.70
Decapoda	763.00	43.00	16.75	0.54	17.00	93.70
Brittle star	0.00	335.20	3.20	0.65	3.20	96.90
Bivalvia	0.00	156.60	1.59	0.49	1.60	98.50
Sea pen	0.00	95.20	0.87	0.39	0.90	99.40
Worm	0.00	32.60	0.31	0.50	0.30	99.70
Gastropoda	0.00	10.80	0.11	0.82	0.20	99.90
Polychaeta	0.00	6.80	0.08	0.37	0.00	99.90

Sea anemone	0.00	6.40	0.07	0.34	0.10	100.00
Food items	*P. indicus*	*P. bindus*	**Average dissimilarity = 84.64%**			
	Avg. Abund.	Avg. Abund.	Avg. Diss.	Diss./SD	Contrib. %	Cum. %
Fish	11070.00	474.20	51.60	1.26	61.00	61.00
Stomatpoda	9863.00	0.00	19.63	0.50	23.20	84.20
Decapoda	763.00	867.60	12.62	0.59	14.90	99.10
Cephalopoda	0.00	29.40	0.33	0.35	0.40	99.50
Ctenopoda	0.00	29.40	0.29	0.36	0.30	99.80
Worm	0.00	10.00	0.10	0.38	0.10	99.90
Isopoda	0.00	2.80	0.03	0.35	0.10	100.00
Gastropoda	0.00	3.00	0.03	0.66	0.00	100.00
Brittle star	0.00	0.80	0.01	0.35	0.00	100.00
Bivalvia	0.00	0.40	0.00	0.36	0.00	100.00
Food items	*P. indicus*	*S. fuscescens*	**Average dissimilarity = 96.86%**			
	Avg. Abund.	Avg. Abund.	Avg. Diss.	Diss./SD	Contrib. %	Cum. %
Fish	11070.00	93.00	57.40	1.23	59.30	59.30
Stomatpoda	9863.00	0.00	20.19	0.50	20.80	80.10
Decapoda	763.00	52.00	19.00	0.54	19.60	99.70
Brittle star	0.00	13.20	0.15	0.48	0.20	99.90
Bivalvia	0.00	2.20	0.03	0.34	0.00	99.90
Gastropoda	0.00	2.20	0.03	0.65	0.00	99.90
Coral	0.00	2.20	0.03	0.42	0.10	100.00
Amphipoda	0.00	1.80	0.02	0.34	0.00	100.00
Copepoda	0.00	0.80	0.01	0.34	0.00	100.00
Food items	*P. indicus*	*D. russelii*	**Average dissimilarity = 94.40%**			
	Avg. Abund.	Avg. Abund.	Avg. Diss.	Diss./SD	Contrib. %	Cum. %
Fish	11070.00	228.80	53.96	1.23	57.20	57.20
Stomatpoda	9863.00	0.00	19.82	0.50	21.00	78.20
Decapoda	763.00	118.40	15.78	0.52	16.70	94.90
Bivalvia	0.00	647.80	4.58	0.40	4.80	99.70
Mollusca	0.00	25.00	0.18	0.39	0.20	99.90
Worm	0.00	11.80	0.08	0.39	0.10	100.00
Brittle star	0.00	0.40	0.00	0.34	0.00	100.00
Food items	*P. indicus*	*G. japonicus*	**Average dissimilarity = 95.78%**			
	Avg. Abund.	Avg. Abund.	Avg. Diss.	Diss./SD	Contrib. %	Cum. %
Fish	11070.00	379.20	56.69	1.31	59.20	59.20
Stomatpoda	9863.00	0.00	20.07	0.50	20.90	80.10
Decapoda	763.00	1.00	18.51	0.55	19.40	99.50

Bivalvia	0.00	50.60	0.51	0.50	0.50	100.00

Food items	*P. indicus*	*T. bimaculatus*	**Average dissimilarity = 89.85%**			
	Avg. Abund.	Avg. Abund.	Avg. Diss.	Diss./SD	Contrib. %	Cum. %
Fish	11070.00	187.00	54.54	1.23	60.70	60.70
Stomatpoda	9863.00	0.00	19.91	0.50	22.20	82.90
Decapoda	763.00	598.00	15.29	0.55	17.00	99.90
Gastropoda	0.00	9.40	0.11	0.63	0.10	100.00

Food items	*T. uranoscopus*	*C. oligolepis*	**Average dissimilarity = 99.78%**			
	Avg. Abund.	Avg. Abund.	Avg. Diss.	Diss./SD	Contrib. %	Cum. %
Fish	215400.00	227.00	92.11	9.74	92.30	92.30
Decapoda	1.00	19090.00	7.66	0.80	7.70	100.00
Stomatpoda	0.00	24.00	0.01	1.22	0.00	100.00
Bivalvia	0.00	1.00	0.00	0.66	0.00	100.00
Brittle star	0.00	1.00	0.00	0.66	0.00	100.00

Food items	*T. uranoscopus*	*P. cinnamoneus*	**Average dissimilarity = 87.58%**			
	Avg. Abund.	Avg. Abund.	Avg. Diss.	Diss./SD	Contrib. %	Cum. %
Fish	215400.00	17830.00	84.80	3.72	96.80	96.80
Decapoda	1.00	3740.00	1.69	0.51	2.00	98.80
Isopoda	0.00	2760.00	0.92	0.48	1.00	99.80
Cephalopoda	0.00	344.00	0.17	0.60	0.20	100.00
Brittle star	0.00	3.00	0.00	0.72	0.00	100.00

Food items	*T. uranoscopus*	*D. punctata*	**Average dissimilarity = 99.98%**			
	Avg. Abund.	Avg. Abund.	Avg. Diss.	Diss./SD	Contrib. %	Cum. %
Fish	215400.00	29.20	99.63	287.50	99.70	99.70
Brittle star	0.00	335.20	0.17	0.86	0.10	99.80
Bivalvia	0.00	156.60	0.08	0.64	0.10	99.90
Sea pen	0.00	95.20	0.05	0.50	0.10	100.00
Decapoda	1.00	43.00	0.02	1.21	0.00	100.00
Worm	0.00	32.60	0.02	0.65	0.00	100.00
Gastropoda	0.00	10.80	0.01	1.40	0.00	100.00
Polychaeta	0.00	6.80	0.00	0.50	0.00	100.00
Sea anemone	0.00	6.40	0.00	0.47	0.00	100.00

Food items	*T. uranoscopus*	*P. bindus*	**Average dissimilarity = 99.52%**			
	Avg. Abund.	Avg. Abund.	Avg. Diss.	Diss./SD	Contrib. %	Cum. %
Fish	215400.00	474.20	99.07	203.30	99.50	99.50
Decapoda	1.00	867.60	0.43	0.99	0.50	100.00
Cephalopoda	0.00	29.40	0.01	0.47	0.00	100.00

Ctenopoda	0.00	29.40	0.01	0.47	0.00	100.00
Worm	0.00	10.00	0.00	0.50	0.00	100.00
Gastropoda	0.00	3.00	0.00	0.99	0.00	100.00
Isopoda	0.00	2.80	0.00	0.47	0.00	100.00
Brittle star	0.00	0.80	0.00	0.47	0.00	100.00
Bivalvia	0.00	0.40	0.00	0.47	0.00	100.00
Food items	*T. uranoscopus*	*S. fuscescens*	**Average dissimilarity = 99.91%**			
	Avg. Abund.	Avg. Abund.	Avg. Diss.	Diss./SD	Contrib. %	Cum. %
Fish	215400.00	93.00	99.87	1048.00	100.00	100.00
Decapoda	1.00	52.00	0.03	0.90	0.00	100.00
Brittle star	0.00	13.20	0.01	0.70	0.00	100.00
Bivalvia	0.00	2.20	0.00	0.50	0.00	100.00
Gastropoda	0.00	2.20	0.00	1.00	0.00	100.00
Coral	0.00	2.20	0.00	0.60	0.00	100.00
Amphipoda	0.00	1.80	0.00	0.50	0.00	100.00
Copepoda	0.00	0.80	0.00	0.50	0.00	100.00
Food items	*T. uranoscopus*	*D. russelii*	**Average dissimilarity = 99.78%**			
	Avg. Abund.	Avg. Abund.	Avg. Diss.	Diss./SD	Contrib. %	Cum. %
Fish	215400.00	228.80	99.38	136.10	99.60	99.60
Bivalvia	0.00	647.80	0.32	0.48	0.30	99.90
Decapoda	1.00	118.40	0.06	1.57	0.10	100.00
Mollusca	0.00	25.00	0.01	0.47	0.00	100.00
Worm	0.00	11.80	0.01	0.47	0.00	100.00
Brittle star	0.00	0.40	0.00	0.47	0.00	100.00
Food items	*T. uranoscopus*	*G. japonicus*	**Average dissimilarity = 99.63%**			
	Avg. Abund.	Avg. Abund.	Avg. Diss.	Diss./SD	Contrib. %	Cum. %
Fish	215400.00	379.20	99.60	190.00	100.00	100.00
Bivalvia	0.00	50.60	0.03	0.66	0.00	100.00
Decapoda	1.00	1.00	0.00	0.92	0.00	100.00
Food items	*T. uranoscopus*	*T. bimaculatus*	**Average dissimilarity = 99.80%**			
	Avg. Abund.	Avg. Abund.	Avg. Diss.	Diss./SD	Contrib. %	Cum. %
Fish	215400.00	187.00	99.51	211.80	99.70	99.70
Decapoda	1.00	598.00	0.29	0.72	0.30	100.00
Gastropoda	0.00	9.40	0.00	0.98	0.00	100.00
Food items	*C. oligolepis*	*P. cinnamoneus*	**Average dissimilarity = 82.16%**			
	Avg. Abund.	Avg. Abund.	Avg. Diss.	Diss./SD	Contrib. %	Cum. %
Decapoda	19090.00	3740.00	48.82	1.31	59.40	59.40

Fish	227.00	17830.00	26.37	0.88	32.10	91.50
Cephalopoda	0.00	344.00	3.83	0.64	4.70	96.20
Isopoda	0.00	2760.00	2.76	0.53	3.30	99.50
Stomatpoda	24.00	0.00	0.33	0.46	0.40	99.90
Brittle star	1.00	3.00	0.05	0.42	0.10	100.00
Bivalvia	1.00	0.00	0.00	0.64	0.00	100.00

Food items	*C. oligolepis*	*D. punctata*	**Average dissimilarity = 95.44%**			
	Avg. Abund.	Avg. Abund.	Avg. Diss.	Diss./SD	Contrib. %	Cum. %
Decapoda	19090.00	43.00	65.67	1.64	68.80	68.80
Fish	227.00	29.20	13.35	0.66	14.00	82.80
Brittle star	1.00	335.20	7.83	0.68	8.20	91.00
Bivalvia	1.00	156.60	4.28	0.48	4.50	95.50
Sea pen	0.00	95.20	1.91	0.38	2.00	97.50
Worm	0.00	32.60	0.81	0.49	0.80	98.30
Stomatpoda	24.00	0.00	0.76	0.66	0.80	99.10
Gastropoda	0.00	10.80	0.33	0.66	0.40	99.50
Polychaeta	0.00	6.80	0.26	0.33	0.20	99.70
Sea anemone	0.00	6.40	0.24	0.31	0.30	100.00

Food items	*C. oligolepis*	*P. bindus*	**Average dissimilarity = 71.04%**			
	Avg. Abund.	Avg. Abund.	Avg. Diss.	Diss./SD	Contrib. %	Cum. %
Decapoda	19090.00	867.60	63.68	1.94	89.60	89.60
Fish	227.00	474.20	4.71	0.79	6.70	96.30
Cephalopoda	0.00	29.40	1.02	0.31	1.40	97.70
Ctenopoda	0.00	29.40	0.69	0.33	1.00	98.70
Stomatpoda	24.00	0.00	0.49	0.69	0.70	99.40
Worm	0.00	10.00	0.23	0.35	0.30	99.70
Isopoda	0.00	2.80	0.10	0.31	0.10	99.80
Gastropoda	0.00	3.00	0.08	0.57	0.10	99.90
Brittle star	1.00	0.80	0.03	0.33	0.10	100.00
Bivalvia	1.00	0.40	0.01	0.41	0.00	100.00

Food items	*C. oligolepis*	*S. fuscescens*	**Average dissimilarity = 87.60%**			
	Avg. Abund.	Avg. Abund.	Avg. Diss.	Diss./SD	Contrib. %	Cum. %
Decapoda	19090.00	52.00	69.83	1.77	79.70	79.70
Fish	227.00	93.00	15.76	0.68	18.00	97.70
Stomatpoda	24.00	0.00	1.05	0.71	1.20	98.90
Brittle star	1.00	13.20	0.54	0.44	0.60	99.50
Bivalvia	1.00	2.20	0.10	0.31	0.20	99.70
Gastropoda	0.00	2.20	0.10	0.55	0.10	99.80

Coral	0.00	2.20	0.09	0.39	0.10	99.90
Amphipoda	0.00	1.80	0.09	0.30	0.10	100.00
Copepoda	0.00	0.80	0.04	0.30	0.00	100.00

Food items	*C. oligolepis*	*D. russelii*	**Average dissimilarity = 82.44%**			
	Avg. Abund.	Avg. Abund.	Avg. Diss.	Diss./SD	Contrib. %	Cum. %
Decapoda	19090.00	118.40	63.14	1.51	76.60	76.60
Fish	227.00	228.80	10.33	0.60	12.50	89.10
Bivalvia	1.00	647.80	7.75	0.39	9.40	98.50
Stomatpoda	24.00	0.00	0.77	0.64	1.00	99.50
Mollusca	0.00	25.00	0.29	0.37	0.30	99.80
Worm	0.00	11.80	0.14	0.37	0.20	100.00
Brittle star	1.00	0.40	0.02	0.34	0.00	100.00

Food items	*C. oligolepis*	*G. japonicus*	**Average dissimilarity = 92.06%**			
	Avg. Abund.	Avg. Abund.	Avg. Diss.	Diss./SD	Contrib. %	Cum. %
Decapoda	19090.00	1.00	70.24	1.89	76.30	76.30
Fish	227.00	379.20	19.44	0.74	21.10	97.40
Bivalvia	1.00	50.60	1.41	0.51	1.60	99.00
Stomatpoda	24.00	0.00	0.97	0.65	1.00	100.00
Brittle star	1.00	0.00	0.00	0.68	0.00	100.00

Food items	*C. oligolepis*	*T. bimaculatus*	**Average dissimilarity = 75.99%**			
	Avg. Abund.	Avg. Abund.	Avg. Diss.	Diss./SD	Contrib. %	Cum. %
Decapoda	19090.00	598.00	65.58	1.73	86.30	86.30
Fish	227.00	187.00	9.32	0.57	12.30	98.60
Stomatpoda	24.00	0.00	0.74	0.67	0.90	99.50
Gastropoda	0.00	9.40	0.35	0.53	0.50	100.00
Bivalvia	1.00	0.00	0.00	0.68	0.00	100.00
Brittle star	1.00	0.00	0.00	0.68	0.00	100.00

Food items	*P. cinnamoneus*	*D. punctata*	**Average dissimilarity = 94.97%**			
	Avg. Abund.	Avg. Abund.	Avg. Diss.	Diss./SD	Contrib. %	Cum. %
Fish	17830.00	29.20	42.01	1.42	44.20	44.20
Decapoda	3740.00	43.00	22.31	0.71	23.50	67.70
Cephalopoda	344.00	0.00	9.00	0.91	9.50	77.20
Brittle star	3.00	335.20	8.89	0.69	9.40	86.60
Bivalvia	0.00	156.60	5.05	0.49	5.30	91.90
Isopoda	2760.00	0.00	3.61	0.64	3.80	95.70
Sea pen	0.00	95.20	2.11	0.39	2.20	97.90
Worm	0.00	32.60	0.95	0.48	1.00	98.90
Gastropoda	0.00	10.80	0.40	0.63	0.40	99.30

Polychaeta	0.00	6.80	0.33	0.33	0.40	99.70
Sea anemone	0.00	6.40	0.31	0.31	0.30	100.00
Food items	*P. cinnamoneus*	*P. bindus*	**Average dissimilarity = 74.46%**			
	Avg. Abund.	Avg. Abund.	Avg. Diss.	Diss./SD	Contrib. %	Cum. %
Fish	17830.00	474.20	35.57	1.17	47.80	47.80
Decapoda	3740.00	867.60	28.87	0.94	38.80	86.60
Cephalopoda	344.00	29.40	5.58	0.93	7.40	94.00
Isopoda	2760.00	2.80	3.22	0.56	4.40	98.40
Ctenopoda	0.00	29.40	0.78	0.33	1.00	99.40
Worm	0.00	10.00	0.26	0.35	0.40	99.80
Gastropoda	0.00	3.00	0.09	0.56	0.10	99.90
Brittle star	3.00	0.80	0.08	0.70	0.10	100.00
Bivalvia	0.00	0.40	0.01	0.33	0.00	100.00
Food items	*P. cinnamoneus*	*S. fuscescens*	**Average dissimilarity = 86.21%**			
	Avg. Abund.	Avg. Abund.	Avg. Diss.	Diss./SD	Contrib. %	Cum. %
Fish	17830.00	93.00	43.85	1.49	50.90	50.90
Decapoda	3740.00	52.00	25.25	0.81	29.20	80.10
Cephalopoda	344.00	0.00	12.00	0.96	14.00	94.10
Isopoda	2760.00	0.00	3.97	0.70	4.60	98.70
Brittle star	3.00	13.20	0.60	0.43	0.70	99.40
Bivalvia	0.00	2.20	0.13	0.30	0.10	99.50
Gastropoda	0.00	2.20	0.13	0.54	0.20	99.70
Amphipoda	0.00	1.80	0.12	0.30	0.10	99.80
Coral	0.00	2.20	0.11	0.39	0.10	99.90
Copepoda	0.00	0.80	0.05	0.30	0.10	100.00
Food items	*P. cinnamoneus*	*D. russelii*	**Average dissimilarity = 83.22%**			
	Avg. Abund.	Avg. Abund.	Avg. Diss.	Diss./SD	Contrib. %	Cum. %
Fish	17830.00	228.80	39.77	1.32	47.80	47.80
Decapoda	3740.00	118.40	22.09	0.72	26.50	74.30
Cephalopoda	344.00	0.00	9.10	0.88	11.00	85.30
Bivalvia	0.00	647.80	8.05	0.39	9.70	95.00
Isopoda	2760.00	0.00	3.63	0.64	4.30	99.30
Mollusca	0.00	25.00	0.30	0.37	0.40	99.70
Worm	0.00	11.80	0.14	0.37	0.10	99.80
Brittle star	3.00	0.40	0.14	0.53	0.20	100.00
Food items	*P. cinnamoneus*	*G. japonicus*	**Average dissimilarity = 92.08%**			
	Avg. Abund.	Avg. Abund.	Avg. Diss.	Diss./SD	Contrib. %	Cum. %
Fish	17830.00	379.20	49.64	1.96	53.90	53.90

Decapoda	3740.00	1.00	25.15	0.80	27.30	81.20
Cephalopoda	344.00	0.00	11.49	0.83	12.50	93.70
Isopoda	2760.00	0.00	3.91	0.69	4.20	97.90
Bivalvia	0.00	50.60	1.70	0.53	1.90	99.80
Brittle star	3.00	0.00	0.19	0.49	0.20	100.00
Food items	*P. cinnamoneus*	*T. bimaculatus*	**Average dissimilarity = 80.14%**			
	Avg. Abund.	Avg. Abund.	Avg. Diss.	Diss./SD	Contrib. %	Cum. %
Fish	17830.00	187.00	38.92	1.26	48.60	48.60
Decapoda	3740.00	598.00	28.25	0.88	35.20	83.80
Cephalopoda	344.00	0.00	8.81	0.93	11.00	94.80
Isopoda	2760.00	0.00	3.59	0.63	4.50	99.30
Gastropoda	0.00	9.40	0.44	0.53	0.50	99.80
Brittle star	3.00	0.00	0.13	0.54	0.20	100.00
Food items	*D. punctata*	*P. bindus*	**Average dissimilarity = 91.24%**			
	Avg. Abund.	Avg. Abund.	Avg. Diss.	Diss./SD	Contrib. %	Cum. %
Decapoda	43.00	867.60	33.34	1.42	36.50	36.50
Fish	29.20	474.20	24.85	1.95	27.30	63.80
Brittle star	335.20	0.80	13.87	1.11	15.20	79.00
Bivalvia	156.60	0.40	7.64	0.72	8.40	87.40
Sea pen	95.20	0.00	3.39	0.54	3.70	91.10
Cephalopoda	0.00	29.40	3.13	0.44	3.40	94.50
Ctenopoda	0.00	29.40	1.71	0.47	1.90	96.40
Worm	32.60	10.00	1.62	0.86	1.80	98.20
Gastropoda	10.80	3.00	0.49	1.37	0.50	98.70
Polychaeta	6.80	0.00	0.46	0.47	0.50	99.20
Sea anemone	6.40	0.00	0.44	0.44	0.50	99.70
Isopoda	0.00	2.80	0.30	0.44	0.30	100.00
Food items	*D. punctata*	*S. fuscescens*	**Average dissimilarity = 82.17%**			
	Avg. Abund.	Avg. Abund.	Avg. Diss.	Diss./SD	Contrib. %	Cum. %
Brittle star	335.20	13.20	27.45	1.68	33.40	33.40
Bivalvia	156.60	2.20	16.89	0.90	20.60	54.00
Fish	29.20	93.00	14.18	0.89	17.20	71.20
Decapoda	43.00	52.00	9.62	0.94	11.70	82.90
Sea pen	95.20	0.00	5.83	0.61	7.10	90.00
Worm	32.60	0.00	3.29	0.76	4.00	94.00
Gastropoda	10.80	2.20	1.45	1.18	1.80	95.80
Polychaeta	6.80	0.00	1.36	0.53	1.70	97.50
Sea anemone	6.40	0.00	1.27	0.48	1.50	99.00

Amphipoda	0.00	1.80	0.37	0.40	0.40	99.40
Coral	0.00	2.20	0.31	0.58	0.40	99.80
Copepoda	0.00	0.80	0.14	0.41	0.20	100.00
Food items	*D. punctata*	*D. russelii*	**Average dissimilarity = 84.10%**			
	Avg. Abund.	Avg. Abund.	Avg. Diss.	Diss./SD	Contrib. %	Cum. %
Bivalvia	156.60	647.80	25.49	0.93	30.30	30.30
Brittle star	335.20	0.40	21.26	1.33	25.30	55.60
Fish	29.20	228.80	19.63	0.93	23.30	78.90
Decapoda	43.00	118.40	7.39	0.86	8.80	87.70
Sea pen	95.20	0.00	4.54	0.56	5.40	93.10
Worm	32.60	11.80	2.42	0.72	2.90	96.00
Gastropoda	10.80	0.00	1.05	0.91	1.30	97.30
Polychaeta	6.80	0.00	0.90	0.46	1.00	98.30
Sea anemone	6.40	0.00	0.84	0.42	1.00	99.30
Mollusca	0.00	25.00	0.57	0.49	0.70	100.00
Food items	*D. punctata*	*G. japonicus*	**Average dissimilarity = 93.32%**			
	Avg. Abund.	Avg. Abund.	Avg. Diss.	Diss./SD	Contrib. %	Cum. %
Brittle star	335.20	0.00	29.50	1.57	31.60	31.60
Fish	29.20	379.20	25.55	0.86	27.40	59.00
Bivalvia	156.60	50.60	16.16	0.88	17.30	76.30
Decapoda	43.00	1.00	9.29	0.80	10.00	86.30
Sea pen	95.20	0.00	5.47	0.60	5.80	92.10
Worm	32.60	0.00	3.24	0.64	3.50	95.60
Gastropoda	10.80	0.00	1.53	0.83	1.60	97.20
Polychaeta	6.80	0.00	1.34	0.46	1.50	98.70
Sea anemone	6.40	0.00	1.24	0.42	1.30	100.00
Food items	*D. punctata*	*T. bimaculatus*	**Average dissimilarity = 86.17%**			
	Avg. Abund.	Avg. Abund.	Avg. Diss.	Diss./SD	Contrib. %	Cum. %
Decapoda	43.00	598.00	29.83	1.15	34.60	34.60
Brittle star	335.20	0.00	20.48	1.34	23.80	58.40
Fish	29.20	187.00	14.79	0.92	17.10	75.50
Bivalvia	156.60	0.00	11.72	0.78	13.70	89.20
Sea pen	95.20	0.00	4.51	0.56	5.20	94.40
Worm	32.60	0.00	2.21	0.71	2.60	97.00
Gastropoda	10.80	9.40	1.03	0.84	1.10	98.10
Polychaeta	6.80	0.00	0.83	0.48	1.00	99.10
Sea anemone	6.40	0.00	0.78	0.44	0.90	100.00
Food items	*P. bindus*	*S. fuscescens*	**Average dissimilarity = 78.15%**			

	Avg. Abund.	Avg. Abund.	Avg. Diss.	Diss./SD	Contrib. %	Cum. %
Decapoda	867.60	52.00	41.15	1.67	52.70	52.70
Fish	474.20	93.00	27.22	1.98	34.80	87.50
Cephalopoda	29.40	0.00	4.68	0.49	6.00	93.50
Ctenopoda	29.40	0.00	2.21	0.49	2.80	96.30
Brittle star	0.80	13.20	0.89	0.65	1.20	97.50
Worm	10.00	0.00	0.74	0.52	0.90	98.40
Isopoda	2.80	0.00	0.45	0.49	0.60	99.00
Gastropoda	3.00	2.20	0.24	0.93	0.30	99.30
Bivalvia	0.40	2.20	0.20	0.49	0.20	99.50
Coral	0.00	2.20	0.16	0.57	0.20	99.70
Amphipoda	0.00	1.80	0.15	0.42	0.20	99.90
Copepoda	0.00	0.80	0.06	0.42	0.10	100.00
Food items	*P. bindus*	*D. russelii*	**Average dissimilarity = 69.21%**			
	Avg. Abund.	Avg. Abund.	Avg. Diss.	Diss./SD	Contrib. %	Cum. %
Decapoda	867.60	118.40	30.74	1.27	44.40	44.40
Fish	474.20	228.80	18.49	1.33	26.70	71.10
Bivalvia	0.40	647.80	13.22	0.51	19.10	90.20
Cephalopoda	29.40	0.00	3.22	0.43	4.70	94.90
Ctenopoda	29.40	0.00	1.70	0.45	2.40	97.30
Worm	10.00	11.80	0.72	0.61	1.10	98.40
Mollusca	0.00	25.00	0.50	0.48	0.70	99.10
Isopoda	2.80	0.00	0.31	0.43	0.40	99.50
Gastropoda	3.00	0.00	0.22	0.82	0.40	99.90
Brittle star	0.80	0.40	0.10	0.51	0.10	100.00
Food items	*P. bindus*	*G. japonicus*	**Average dissimilarity = 83.82%**			
	Avg. Abund.	Avg. Abund.	Avg. Diss.	Diss./SD	Contrib. %	Cum. %
Decapoda	867.60	1.00	41.10	1.68	49.00	49.00
Fish	474.20	379.20	32.28	1.96	38.50	87.50
Cephalopoda	29.40	0.00	4.42	0.44	5.30	92.80
Bivalvia	0.40	50.60	2.50	0.76	3.00	95.80
Ctenopoda	29.40	0.00	2.02	0.47	2.40	98.20
Worm	10.00	0.00	0.68	0.49	0.80	99.00
Isopoda	2.80	0.00	0.42	0.44	0.50	99.50
Gastropoda	3.00	0.00	0.28	0.81	0.40	99.90
Brittle star	0.80	0.00	0.12	0.44	0.10	100.00
Food items	*P. bindus*	*T. bimaculatus*	**Average dissimilarity = 57.84%**			
	Avg. Abund.	Avg. Abund.	Avg. Diss.	Diss./SD	Contrib. %	Cum. %

Decapoda	867.60	598.00	33.54	1.46	58.00	58.00
Fish	474.20	187.00	18.12	1.42	31.30	89.30
Cephalopoda	29.40	0.00	3.04	0.45	5.30	94.60
Ctenopoda	29.40	0.00	1.69	0.47	2.90	97.50
Worm	10.00	0.00	0.57	0.50	1.00	98.50
Gastropoda	3.00	9.40	0.49	0.81	0.80	99.30
Isopoda	2.80	0.00	0.29	0.45	0.50	99.80
Brittle star	0.80	0.00	0.08	0.45	0.20	100.00
Bivalvia	0.40	0.00	0.02	0.47	0.00	100.00

Food items	*S. fuscescens*	*D. russelii*	**Average dissimilarity = 64.35%**			
	Avg. Abund.	Avg. Abund.	Avg. Diss.	Diss./SD	Contrib. %	Cum. %
Fish	93.00	228.80	27.95	1.22	43.40	43.40
Bivalvia	2.20	647.80	17.89	0.55	27.80	71.20
Decapoda	52.00	118.40	14.27	1.02	22.20	93.40
Brittle star	13.20	0.40	2.01	0.69	3.10	96.50
Mollusca	0.00	25.00	0.64	0.49	1.00	97.50
Gastropoda	2.20	0.00	0.41	0.78	0.60	98.10
Amphipoda	1.80	0.00	0.41	0.39	0.70	98.80
Coral	2.20	0.00	0.33	0.57	0.50	99.30
Worm	0.00	11.80	0.30	0.49	0.50	99.80
Copepoda	0.80	0.00	0.16	0.40	0.20	100.00

Food items	*S. fuscescens*	*G. japonicus*	**Average dissimilarity = 91.90%**			
	Avg. Abund.	Avg. Abund.	Avg. Diss.	Diss./SD	Contrib. %	Cum. %
Fish	93.00	379.20	57.64	1.76	62.70	62.70
Decapoda	52.00	1.00	20.69	0.66	22.50	85.20
Bivalvia	2.20	50.60	7.27	1.14	7.90	93.10
Brittle star	13.20	0.00	3.72	0.83	4.10	97.20
Amphipoda	1.80	0.00	0.93	0.39	1.00	98.20
Gastropoda	2.20	0.00	0.78	0.74	0.90	99.10
Coral	2.20	0.00	0.57	0.61	0.60	99.70
Copepoda	0.80	0.00	0.30	0.40	0.30	100.00

Food items	*S. fuscescens*	*T. bimaculatus*	**Average dissimilarity = 66.62%**			
	Avg. Abund.	Avg. Abund.	Avg. Diss.	Diss./SD	Contrib. %	Cum. %
Decapoda	52.00	598.00	41.87	1.55	62.80	62.80
Fish	93.00	187.00	20.22	1.14	30.40	93.20
Brittle star	13.20	0.00	1.85	0.72	2.80	96.00
Gastropoda	2.20	9.40	1.53	0.81	2.30	98.30
Bivalvia	2.20	0.00	0.37	0.42	0.50	98.80

Amphipoda	1.80	0.00	0.34	0.41	0.60	99.40
Coral	2.20	0.00	0.30	0.59	0.40	99.80
Copepoda	0.80	0.00	0.14	0.42	0.20	100.00
Food items	*D. russelii*	*G. japonicus*	**Average dissimilarity = 87.80%**			
	Avg. Abund.	Avg. Abund.	Avg. Diss.	Diss./SD	Contrib. %	Cum. %
Fish	379.20	228.80	46.19	1.57	52.60	52.60
Decapoda	1.00	118.40	20.32	0.88	23.20	75.80
Bivalvia	50.60	647.80	20.31	0.69	23.10	98.90
Mollusca	0.00	25.00	0.61	0.49	0.70	99.60
Worm	0.00	11.80	0.29	0.49	0.30	99.90
Brittle star	0.00	0.40	0.08	0.42	0.10	100.00
Food items	*D. russelii*	*T. bimaculatus*	**Average dissimilarity = 63.52%**			
	Avg. Abund.	Avg. Abund.	Avg. Diss.	Diss./SD	Contrib. %	Cum. %
Decapoda	118.40	598.00	27.46	1.04	43.20	43.20
Fish	228.80	187.00	18.67	0.97	29.40	72.60
Bivalvia	647.80	0.00	15.32	0.52	24.10	96.70
Gastropoda	0.00	9.40	1.19	0.77	1.90	98.60
Mollusca	25.00	0.00	0.57	0.48	0.90	99.50
Worm	11.80	0.00	0.27	0.48	0.40	99.90
Brittle star	0.40	0.00	0.05	0.44	0.10	100.00
Food items	*G. japonicus*	*T. bimaculatus*	**Average dissimilarity = 91.30%**			
	Avg. Abund.	Avg. Abund.	Avg. Diss.	Diss./SD	Contrib. %	Cum. %
Decapoda	1.00	598.00	45.36	1.47	49.70	49.70
Fish	379.20	187.00	39.86	1.55	43.60	93.30
Bivalvia	50.60	0.00	4.27	0.88	4.70	98.00
Gastropoda	0.00	9.40	1.81	0.76	2.00	100.00

Avg. Abund. = average abundance, Avg. Diss. = average dissimilarity, Diss./SD = average contribution divided by standard deviation, Contrib. % = contribution to the dissimilarities, Cum. % = cumulative contribution to the dissimilarities.
